# Pincer‐Supported Gallium Complexes for the Catalytic Hydroboration of Aldehydes, Ketones and Carbon Dioxide

**DOI:** 10.1002/chem.202103009

**Published:** 2021-10-27

**Authors:** Lingyu Liu, Siu‐Kwan Lo, Cory Smith, Jose M. Goicoechea

**Affiliations:** ^1^ Department of Chemistry University of Oxford Chemistry Research Laboratory 12 Mansfield Rd. Oxford OX1 3TA UK

**Keywords:** alkoxides, carbonyls, catalysis, gallium, hydrides

## Abstract

Gallium hydrides stabilised by primary and secondary amines are scarce due to their propensity to eliminate dihydrogen. Consequently, their reactivity has received limited attention. The synthesis of two novel gallium hydride complexes HGa(THF)[ON(H)O] and H_2_Ga[μ^2^‐ON(H)O]Ga[ON(H)O] ([ON(H)O]^2−^=*N*,*N*‐bis(3,5‐di‐*tert*‐butyl‐2‐phenoxy)amine) is described and their reactivity towards aldehydes and ketones is explored. These reactions afford alkoxide‐bridged dimers through 1,2‐hydrogallation reactions. The gallium hydrides can be regenerated through Ga−O/B−H metathesis from the reaction of such dimers with pinacol borane (HBpin) or 9‐borabicyclo[3.3.1]nonane (9‐BBN). These observations allowed us to target the catalytic reduction of carbonyl substrates (aldehydes, ketones and carbon dioxide) with low catalyst loadings at room temperature.

## Graphical abstract

**The synthesis of two novel gallium hydride complexes** supported by an *N*,*N*‐bis(3,5‐di‐*tert*‐butyl‐2‐phenoxy)amine pincer ligand is described and their activity as catalysts for the catalytic reduction of aldehydes, ketones and carbon dioxide is explored.

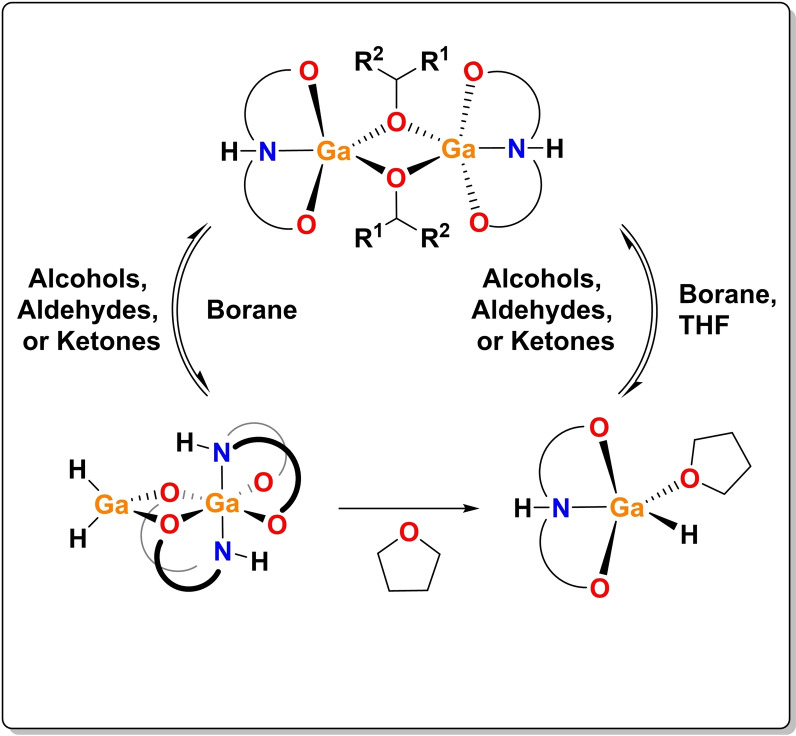

## Introduction

**Figure 1:**
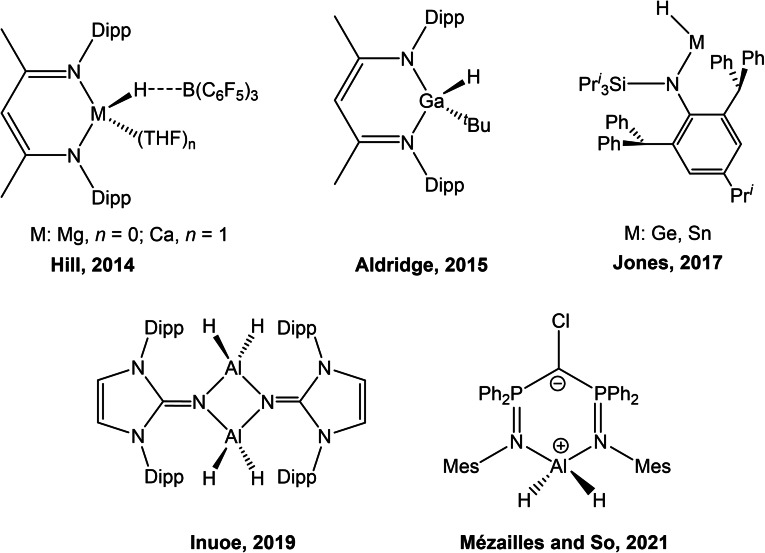
Examples of single component metal(loid) main group hydrides that catalyse the reduction of carbon dioxide in the presence of boranes.

The catalytic conversion of CO_2_ into value‐added chemicals is a pressing challenge given the detrimental environmental impact of rising greenhouse gas emissions.^[1]^ By and large, efforts into the chemical utilisation of carbon dioxide have focused on its use as a C_1_ feedstock for the synthesis of, for example, methane, formic acid, formaldehyde, and methanol.^[2]^ The majority of research carried out in this field has been in the fields of heterogeneous catalysis and electro‐catalysis.^[3]^ In the field of homogenous catalysis, a number of transition metal based compounds have also been successfully employed for the transformation of CO_2_ using a hydroboration strategy.[^4^, ^5^] Dual component catalysts based on the main group elements, so‐called frustrated Lewis pairs (FLPs), have also been shown to be active for the conversion of carbon dioxide to a number of products, including methanol‐equivalents (CH_3_OBR_2_).[^4^, ^6^] By contrast, single component main group compounds for the catalytic conversion of CO_2_ to methanol equivalents in the presence of boranes remain rare. To the best of our knowledge, there are only five such compounds reported to date. These include beta‐diketiminato supported main group hydride complexes such as Hill's (^Dipp^Nacnac)M(THF)_*n*_(μ‐H)B(C_6_F_5_)_3_ (M=Mg, and *n*=0; M=Ca, and *n*=1) and Aldridge's (^Dipp^Nacnac)Ga(H)(^*t*^Bu),[^7^, ^8^] and two‐coordinate germanium(II) and tin(II) hydrides supported by sterically demanding bulky amide ligands reported by Jones and co‐workers (Figure 1).^[9]^ The most recent examples of such compounds are Inoue's dimeric N‐heterocyclic imine supported aluminium dihydride,^[10]^ as well as Mézailles and So's bis(phosphoranyl)methanido aluminium hydride (also Figure 1).^[11]^

Main group hydrides are attractive candidates for the catalytic activation of carbon dioxide on account of their weak and highly polarized ^δ+^M‐H^δ−^ bonds, which readily undergo insertion (1,2‐hydrometallation) reactions. The use of such compounds for hydride transfer reactions such as hydroboration, hydrosilylation and hydrogenation has been extensively explored over the last ten years.[^4^, ^12^, ^13^, ^14^] There are a number of reports in the chemical literature describing the hydroboration of unsaturated C=E bonds (E=C, N, O) using magnesium,[^7^, ^15^, ^16^, ^17^, ^18^, ^19^, ^20^, ^21^] calcium,[^7^, ^22^, ^23^] aluminium,[^10^, ^24^, ^25^, ^26^, ^27^, ^28^, ^29^, ^30^, ^31^, ^32^, ^33^] germanium and tin catalysts.[^9^, ^34^, ^35^, ^36^, ^37^] By contrast, the employment of gallium compounds in catalytic hydroboration reactions remains rare, and only a handful of examples have been reported. In addition to Aldridge's (^Dipp^Nacnac)Ga(H)(^*t*^Bu) complex, which is catalytically active in the reduction of carbon dioxide,^[8]^ Woodward and co‐workers reported that LiGaH_4_ in combination with a monothiobinaphthol (MTB) ligand or S, O‐chelate 2‐hydroxy‐2′‐mercapto‐1,1′‐binaphthyl (MTBH_2_) ligand could be active catalysts for the asymmetric hydroboration of ketones.[^38^, ^39^] More recently, a further report by Aldridge exploring the activity of beta‐diketiminato gallium complexes showed that a three‐coordinate gallium cation accompanied by a hydroborate counter‐ion could be used for the catalytic hydrosilylation of carbon dioxide.^[40]^

Evidently, examples of gallium hydrides for C=E (E=C, O, N) bond activation at room temperature remain very limited, therefore there is a wide scope in this area to be explored. Herein we report the efficient catalytic hydroboration of carbonyl substrates, such as aldehydes, ketones and carbon dioxide, using a gallium hydride catalyst stabilised by [ON(H)O]^2−^ (*N*,*N*‐bis(3,5‐di‐*tert*‐butyl‐2‐phenoxy)amine ligand).

## Results and Discussion

**Scheme 1:**
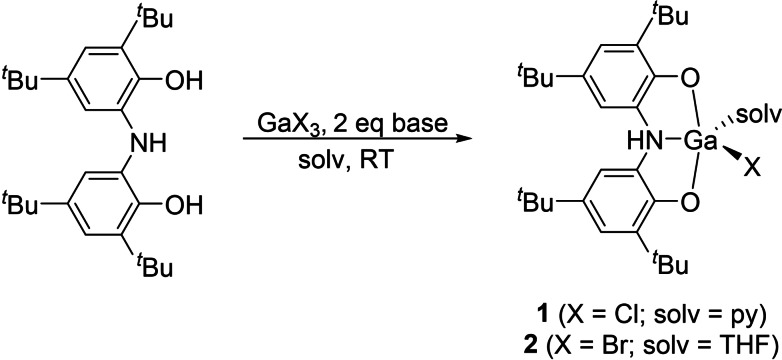
Synthesis of **1** and **2**.

The reaction of H_3_(ONO), GaX_3_ (where X=Cl, Br) and two equivalents of triethylamine (for the synthesis of **1**) or KH (for the synthesis of **2**) in the presence of a donor solvent (pyridine or THF) quantitatively affords a single product (Scheme 1) as evidenced by a single new ligand environment in the ^1^H NMR spectrum in C_6_D_6_.^[41]^ These compounds exhibit two resonances for the aromatic protons of the ligand backbone (**1**: 7.41, 7.22 ppm; **2**: 7.38, 7.13 ppm), two signals for the inequivalent *tert*‐butyl groups (**1**: 1.53, 1.38 ppm; **2**: 1.55, 1.35 ppm) and a broad resonance arising from the proton associated with the secondary amine of the ligand backbone (**1**: 4.61; **2**: 4.56 ppm). Analogous base‐stabilised aluminium complexes of this ligand have previously been reported by Heyduk and the NMR spectroscopic data reported found to be comparable to that of **1** and **2**.^[42]^ The resulting gallium(III) compounds **1** and **2** were crystallographically characterized (see Supporting Information), revealing very similar geometries with relatively short Ga−O bonds (**1**: 1.846(1) Å; **2**: 1.833(2) and 1.837(2) Å) and longer dative Ga−N interactions (**1**: 2.142(1) Å; **2**: 2.120(3) Å).

**Scheme 2:**
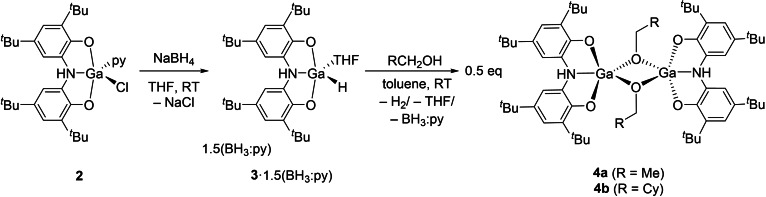
Synthesis of **3** ⋅ 1.5(BH_3_:py) and subsequent alcoholysis to afford **4** **a** and **4** **b**.

Reaction of **1** with NaBH_4_ (Scheme 2) affords a novel gallium‐hydride, **3** ⋅ 1.5(BH_3_:py), in which the ligand backbone remains protonated at the nitrogen atom. As with **1** and **2**, this species is characterized by two sets of two resonances for the aromatic and *tert*‐butyl protons. In addition, resonances corresponding to the hydride and the amine backbone and are observed at 5.67 and 4.01 ppm in C_6_D_6_, respectively. The ^1^H NMR spectrum of **3** ⋅ 1.5(BH_3_:py) indicated the presence of the borane‐pyridine adduct in the form of a 1 : 1 : 1 : 1 quartet at 3.49 ppm (with a ^1^ *J*_B‐H_ coupling constant of 100.4 Hz). This adduct is also present in the crystalline structure of **3** ⋅ 1.5(BH_3_:py) (see Supporting Information) which reveals 1.5 equivalents of BH_3_:py in the asymmetric unit. One of the crystallographically unique BH_3_:py units interacts with **3** via a hydrogen bond. A hydridic hydrogen atom on BH_3_ is interacting with the protic hydrogen atom on N−H of the ligand backbone of **3** with a distance of 2.00(4) Å (*d*_B1 ⋅ ⋅ ⋅ N1_=3.527(6) Å). Compound **3** is an interesting species insomuch as it contains a hydridic Ga−H bond in close proximity to an acidic N−H bond in the ligand backbone. Adducts of gallium hydrides with primary and secondary amines such as L ⋅ GaH_3_ (L=NHMe_2_, NH^*t*^Bu_2_, NH_2_Me, NH_2_^*t*^Bu, NH_2_^*s*^Bu),[^43^, ^44^, ^45^] and cationic systems such as [H_2_Ga(NH_2_R)_2_]Cl (R=Me, ^*i*^Pr, ^*t*^Bu, ^*s*^Bu),[^46^, ^47^] and {[2,6‐(Me_2_N(H)CH_2_)_2_C_6_H_3_]Ga(H)(OTf)_2_}(OTf),^[48]^ are relatively rare and can, in principle, eliminate dihydrogen. The thermolysis of gallium hydride complexes has been well‐documented.[^49^, ^50^, ^51^]

**Figure 2:**
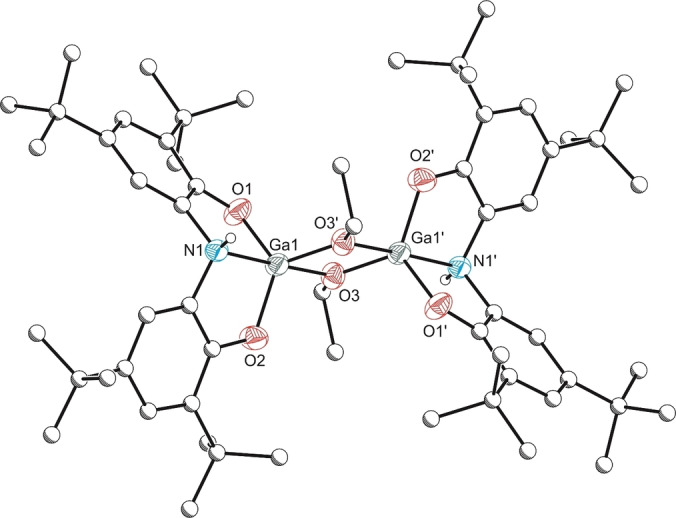
Molecular structure of **4** **a**. Anisotropic displacement ellipsoids set at 50 % probability. Hydrogen atoms (with the exception of H1) have been omitted for clarity. All carbon atoms are pictured as spheres of arbitrary radius. Selected interatomic distances [Å] and angles [°]: Ga1‐O1 1.838(2), Ga1‐O2 1.836(2), Ga1‐N1 2.149(2), Ga1‐O3 1.960(2), Ga1‐O3′ 1.878(2); O1‐Ga1‐O2 121.24(10), O1‐Ga1‐N1 85.05(8), O1‐Ga1‐O3 94.43(8), O1‐Ga1‐O3′ 117.99(9), O2‐Ga1‐N1 85.20(8), O2‐Ga1‐O3 96.15(8), O2‐Ga1‐O3′ 120.76(9), O3‐Ga1‐N1 178.63(7), O3′‐Ga1‐N1 100.89(8), O3‐Ga1‐O3′ 78.22(8). Symmetry operation ′: 1‐x, 2‐y ‐z.

**3** ⋅ 1.5(BH_3_:py) reacts with alcohols such as ethanol and cyclohexylmethanol to afford bimetallic bis‐alkoxide compounds **4** **a** and **4** **b**, respectively (Scheme 2). The ^1^H NMR spectra of these compounds do not differ greatly from **1**–**3** and reveal the characteristic broad resonance corresponding to the amine proton of the *N*,*N*‐bis(3,5‐di‐*tert*‐butyl‐2‐phenoxy)amine ligand at 5.05 and 4.97 ppm for **4** **a** and **4** **b** in CDCl_3_, respectively. Both samples were characterized by single crystal X‐ray crystallography confirming a bimetallic structure bridged by two μ^2^‐OR ligands. The crystal structure of **4** **a** is shown in Figure 2. The gallium(III) centres in **4** **a** are five‐coordinate with distorted pentagonal bipyramidal geometries (τ_5_=0.96) with N1 and O3 occupying the apical positions.

**Scheme 3:**
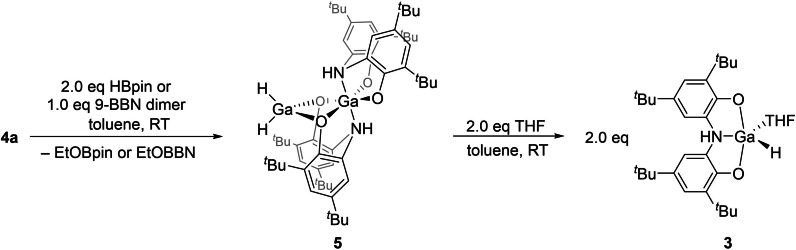
Synthesis of **5** and formation of a base‐stabilised monometallic hydride by addition of THF to afford **3**.

**Figure 3:**
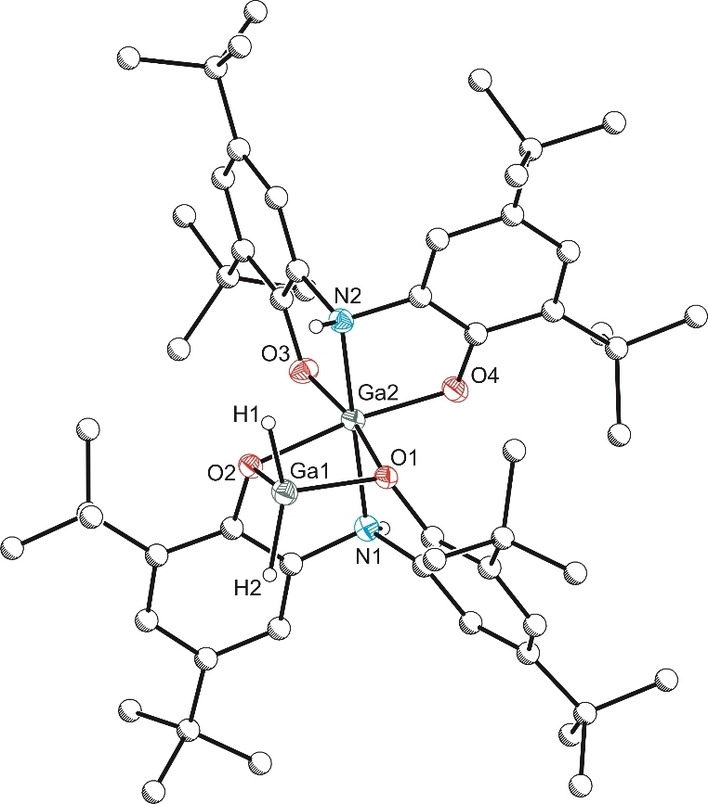
Molecular structure of **5**. Anisotropic displacement ellipsoids set at 50 % probability. Hydrogen atoms (with the exception of H1, H2 and amine ligand backbone) have been omitted for clarity. All carbon atoms are pictured as spheres of arbitrary radius. Selected interatomic distances [Å] and angles [°]: Ga1‐O1 1.928(2), Ga1‐O2 1.951(2), Ga2‐O3 1.872(2), Ga2‐O4 1.883(2), Ga2‐N1 2.045(2), Ga2‐N2 2.053(2), Ga2‐O2 2.085(2), Ga2‐O1 2.131(2); O1‐Ga1‐O2 84.88(8), O3‐Ga2‐O4 101.22(9), O3‐Ga2‐N1 102.18(9), O4‐Ga2‐N1 95.63(9), O3‐Ga2‐N2 87.97(9), O4‐Ga2‐N2 86.35(9), N1‐Ga2‐N2 169.02(10), O3‐Ga2‐O2 92.02(8), O4‐Ga2‐O2 166.76(8), N1‐Ga2‐O2 81.71(9), N2‐Ga2‐O2 93.89(9), O3‐Ga2‐O1 168.18(8), O4‐Ga2‐O1 90.04(8), N1‐Ga2‐O1 80.06(9), N2‐Ga2‐O1 89.16(8), O2‐Ga2‐O1 76.73(7).

Subsequent reaction of **4** **a** with two equivalents of HBpin or one equivalent of 9‐borabicyclo[3.3.1]nonane (9‐BBN) in toluene afforded a novel gallium dihydride complex **5** (Scheme 3). Crystallisation of **5** from hexane at −30 °C provided crystals suitable for single crystal X‐ray diffraction analysis. The analysis confirms that, in the solid state, compound **5** consists of two gallium atoms linked by two μ^2^‐O bridging atoms, with one tetrahedral gallium dihydride and a distorted octahedral gallium centre (Figure 3).

The solution behaviour of compound **5** is intriguing, as compositionally pure crystalline samples of **5** were found to give rise to four distinct isomers in solution as determined by ^1^H NMR spectroscopy. Based on the crystallographically determined structure, the ^1^H NMR spectrum of **5** is expected to exhibit two gallium hydride resonances and two resonances for the magnetically inequivalent amine backbone protons. In the expected region for these resonances (3.8 to 6.8 ppm), there are three species (**5_II_**, **5_III_**, **5_IV_**) which exhibit the requisite number of resonances, as well as a more abundant isomer (**5_I_**) with only one resonance for the N−H and Ga−H hydrogen atoms (4.02 and 5.55 ppm, respectively). We have tentatively assigned this compound as a monomeric species. The remaining three isomers, were assigned as follows: **5_II_** (NH: 5.11, 4.82 ppm; GaH: 5.94, 5.86 ppm), **5_III_** (NH: 4.67, 4.56 ppm; GaH: 5.80, 5.90 ppm), and **5_IV_** (NH: 4.70, 4.67 ppm; GaH: 6.62, 6.50 ppm). At room temperature, these species integrate in a ratio of 13 : 3 : 1.5 : 1. On heating a *d*_8_‐toluene solution of this isomeric mixture to 333 K all isomers convert to **5_I_**. One‐dimensional ^1^H NOESY and two‐dimensional ^1^H‐^1^H NOESY experiments were carried to establish that all four isomers are exchanging in solution. Several attempts were made to crystallize other isomers from solutions of **5**, however, only the structure shown in Figure 3 could be obtained.

In order to further probe the solution‐phase structure of **5**, diffusion‐ordered ^1^H NMR spectra were recorded for C_6_D_6_ solutions of **5** and **4** **a** (see Supporting Information for full details). The two major isomers of **5** in solution, **5_I_** and **5_II_**, have markedly different diffusion coefficients: 7.8 ⋅ 10^−10^ and 5.8 ⋅ 10^−10^ m^2^ ⋅ s^−1^, respectively (the latter being identical to the value determined for solutions of **4** **a**). According to the Stokes‐Einstein Gierer‐Wirtz Estimation (SEGWE), these values correspond to molecular weights of 445 and 846 g ⋅ mol^−1^ for **5_I_** and **5_II_**,[^52^, ^53^] supporting the hypothesis that **5_I_** is a monomeric hydride (i. e. HGa[ON(H)O]) while **5_II_** has a structure consistent with a bimetallic compound elucidated by single crystal X‐ray diffraction. The calculated molecular weights are in relatively good agreement with the molecular weights for monomeric and dimeric gallium hydrides: 494.3 and 988.7 g ⋅ mol^−1^. With these data in hand, we conclude that, at room temperature, compound **5** exists in equilibrium between a monomeric form (**5_I_**) and multiple dimeric isomers (**5_II_**, **5_III_** and **5_IV_**). This is supported by the fact that on heating solutions of **5** above 333 K the monomer, **5_I_**, is favoured. It seems reasonable that one of the dimeric forms of **5** should have a structure similar to that determined by single‐crystal X‐ray diffraction, i. e. H_2_Ga[μ^2^‐ON(H)O]Ga[ON(H)O], however the nature of the other isomers is unclear at this stage, and computational studies were inclusive.

**Figure 4:**
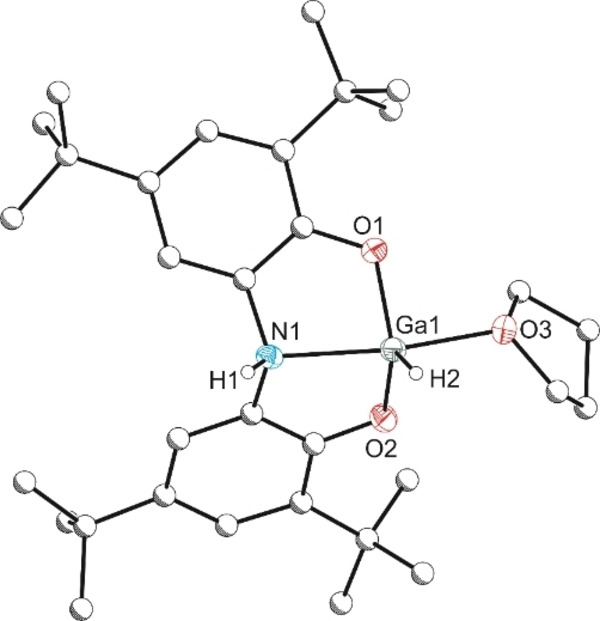
Molecular structure of **3**. Anisotropic displacement ellipsoids set at 50 % probability. Hydrogen atoms (with the exception of H1 and H2) have been omitted for clarity. All carbon atoms are pictured as spheres of arbitrary radius. Selected interatomic distances [Å] and angles [°]: Ga1‐O1 1.857(2), Ga1‐O2 1.865(2), Ga1‐O3 2.111(2), Ga1‐N1 2.191(2); O1‐Ga1‐O2 113.54(9), O1‐Ga1‐O3 85.54(9), O2‐Ga1‐O3 87.35(9), O1‐Ga1‐N1 83.10(9), O2‐Ga1‐N1 83.35(9), O3‐Ga1‐N1 161.03(9).

Further evidence to support that the solution NMR spectrum of **5** arises due to the presence of multiple isomers of the same compound was obtained by addition of a stoichiometric amount (1 equiv. per gallium centre) of THF to a solution of **5**. This gave rise to a single product which exhibits the presence of a single ligand environment (7.15 and 7.35 ppm), a broad gallium hydride resonance at 5.65 ppm, and an amine backbone resonance at 3.92 ppm. This is broadly similar to what was observed previously for **3** ⋅ 1.5(BH_3_:py), and therefore can be assigned to complex **3** (Scheme 3). The structure of this compound was confirmed by single crystal X‐ray diffraction analysis (Figure 4).

Having identified two novel gallium hydrides, we sought to investigate their reactivity towards carbonyl‐containing substrates. Thus, a 1 : 1 stoichiometric reaction of benzaldehyde and **3** at room temperature in C_6_D_6_ showed complete consumption of **3** over 12 h as evidenced by the disappearance of the Ga−H resonance in the ^1^H NMR spectrum at 5.65 ppm. Single crystals were obtained from the reaction mixture and XRD analysis identified the formation of gallium alkoxide complex **6** **a**. The ^1^H NMR spectrum of **6** **a** reveals the amine resonance at 3.93 ppm and two sets of resonances for the *tert*‐butyl protons at 1.27 ppm and 1.39 ppm in CDCl_3_.

**Scheme 4:**
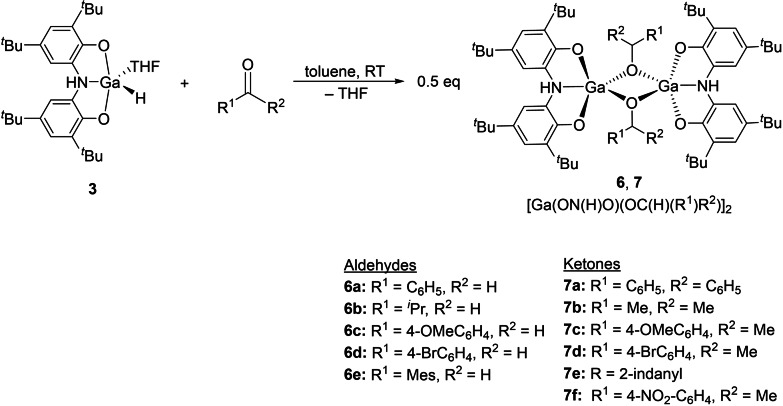
Synthetic route to compounds **6** and **7**.

**Scheme 5:**
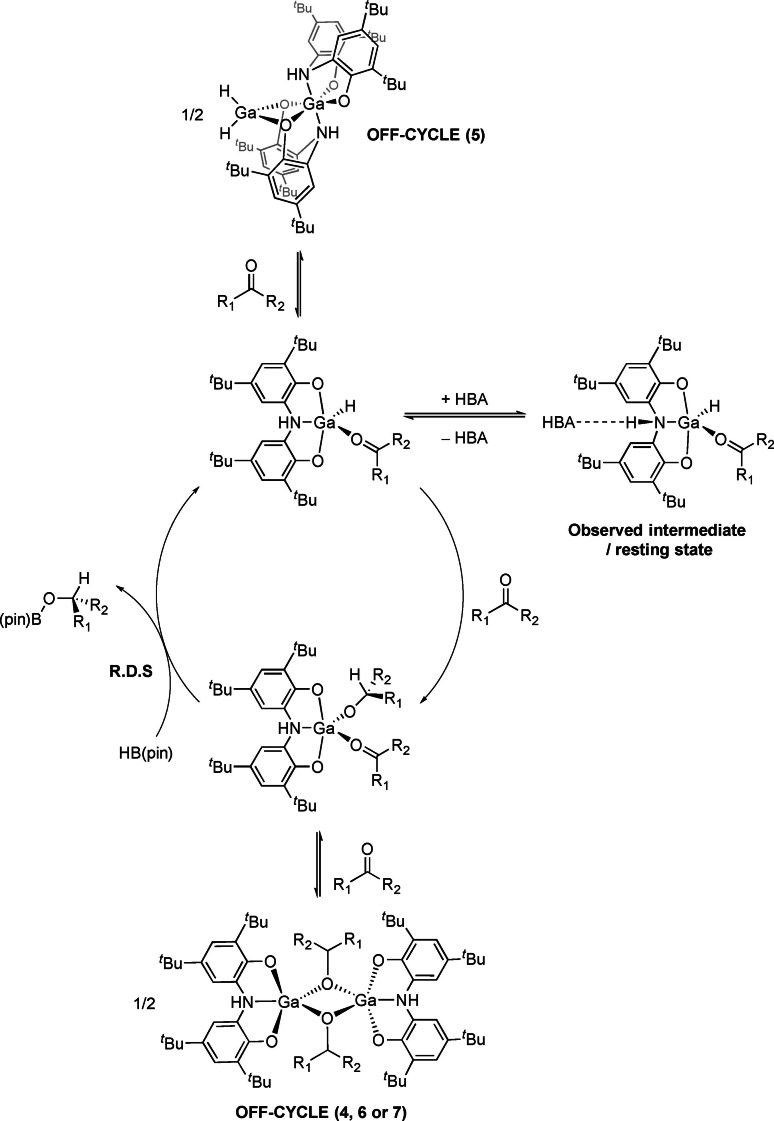
Proposed hydroboration mechanism for aldehyde and ketone substrates.

**Scheme 6:**
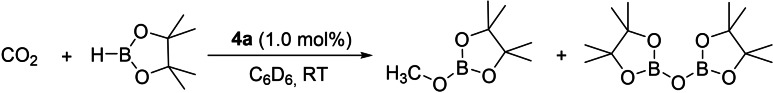
Catalytic hydroboration of carbon dioxide to MeOBpin and O(Bpin)_2_ (when conducted in the absence of **4** **a**, no conversion was observed).

These results are consistent with hydrogallation of the C=O bond of benzaldehyde. The same result was observed on reaction of **5** with two equivalents of benzaldehyde. This reaction is broadly applicable to a wide range of aldehydes (Scheme 4,5,6; see Supporting Information for full details) including species with non‐aromatic R‐substituents and compounds bearing halide and methoxy‐functional groups.

The reactivity of complexes **3** and **5** was also explored towards ketones (Scheme 4), which typically proceed much slower than the reactions with aldehydes. A series of stochiometric reactions (per gallium centre) between **3** or **5** and six different ketones were undertaken, all of which afforded dimeric gallium alkoxide complexes, similar to the aforementioned reactions with aldehydes. The gallium alkoxide species **7** **a**–**7** **f** were isolated and fully characterized including by single crystal X‐ray diffraction (see the Supporting Information).

Encouraged by these results, we looked at extending this reactivity to develop a catalytic protocol for the reduction of carbonyls. In a typical reaction 0.05 mol% of pre‐catalyst of **4** **a** was added to a 1 : 1.1 mixture of a ketone and pinacol borane in C_6_D_6_ solution at room temperature (Table 1). The reactions were monitored by ^1^H NMR spectroscopy and referenced to an internal standard (hexamethylcyclotrisiloxane; HMCTS). Full conversion of the ketones to the corresponding borate esters was observed over 7–10 h (see Supporting Information).

**Table 1:** Scope of gallium‐catalysed hydroboration of ketones.^[a]^

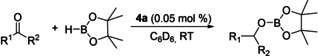				
Entry	R^1^	R^2^	Time [h]	Conv. [%]^[b]^
1	4‐NO_2_−C_6_H_5_	Me	7	99
2	C_6_H_5_	Me	10	99
3	4‐OMe−C_6_H_5_	Me	10	92
4	4‐Br−C_6_H_5_	Me	10	99
5	4‐Cl−C_6_H_5_	Me	10	99
6	4‐I−C_6_H_5_	Me	10	98

[a] Catalyst loading (0.05 mol%). Ketone (0.5 mmol) and HBpin (0.55 mmol, 80 μL) in C_6_D_6_ (0.5 mL), HMCTS (0.05 mmol) as internal standard, reactions were performed at room temperature. [b] Conversion is calculated by integration relative to internal standard HMCTS. Note: when conducted in the absence of **4** **a**, less than 1 % conversion was observed for these transformations after 24 h.

Our catalyst exhibits an overall turnover frequency of ∼200 h^−1^ towards functionalised acetophenones, and is thus faster than Hill's magnesium catalyst (acetophenone, TOF=∼23.5 h^−1^)^[17]^ and Jones’ germanium catalyst (4‐methoxy acetophenone, TOF=∼30 h^−1^),^[37]^ but not as efficient as Jones’ tin catalyst (4‐methoxy acetophenone, TOF=∼800 h^−1^).^[37]^ This shows that the gallium complexes discussed previously are effective catalysts for the hydroboration of ketones. While monitoring the catalysis by ^1^H NMR spectroscopy, we observed a gallium hydride complex intermediate (with Ga−H resonance at 5.76 ppm) which is presumably involved in the catalytic cycle (see the Supporting Information). More accessible aldehyde substrates can be hydroborated to borylated products within 1 to 12 h using 0.05 mol% of pre‐catalyst **6** **a** (note: in the catalytic reduction of aldehydes, **6** **a** was used as opposed to **4** **a**).

Thomas and co‐workers have previously argued that nucleophiles can induce the decomposition of pinacol borane to BH_3_, and that it is the latter species that acts as a “hidden” catalyst in many transformations typically attributed to other compounds.^[54]^ In order to rule out the presence of BH_3_ in our reaction mixtures, we explored the hydroboration 4′‐nitroacetophenone in the presence of an excess of tetramethylethylenediamine (TMEDA). TMEDA is known to coordinate to BH_3_ and can thus be used as a qualitative probe of whether BH_3_ is actively involved in a catalytic reaction. From our studies (see Supporting Information) we see a moderate reduction of catalytic activity in the first hour, which rules out the presence of BH_3_ as the sole active catalyst. The reduction in conversion may be attributed to the coordination of TMEDA to the gallium‐hydride catalyst present in our reaction mixtures.

A simplified mechanistic model for these gallium catalysed hydroboration reactions can be proposed involving the following key steps: (i) an alkoxide pre‐catalyst reacts with pinacol borane to generate a gallium hydride, (ii) insertion of the C=O bond into the Ga−H bond affords a gallium alkoxide complex, (iii) σ‐metathesis of Ga−O bond with pinacol borane regenerates the hydride and liberates the borate ester. The question that arises at this stage is speciation during the catalytic process (i. e. is the active catalyst monomeric or dimeric?). Based on the DOSY NMR experiments described earlier (see above), the presence of a monometallic catalyst seems entirely viable. In an effort to address this question, we carried out a series of experiments using 4′‐nitroacetophenone as a model substrate. In the first instance we compared three different pre‐catalysts: the monomeric species **3** (at 4 mol% catalyst loading), and the bimetallic compounds **4** **a** and **5** (both at 2 mol% loading). The concentration plots (recorded by integration relative to an HMCTS internal standard) of these three catalytic runs are largely identical. This supports the hypothesis that under catalytic conditions (i. e. in the presence of a vast excess of ketone) a monometallic catalyst may be operating (Scheme 5). It is worth noting that in all of these runs only one intermediate is observed by ^1^H NMR spectroscopy, regardless of the pre‐catalyst employed. NMR data suggest that this intermediate (a possible off‐cycle species), is a gallium hydride in which the amine ligand backbone is interacting with a hydrogen bond acceptor (HBA; i. e. HBpin, ketone or the borate ester). This is evident by the fact that the observed resting state exhibits an NMR fingerprint that is comparable to that of the monomeric compound **3** (i. e. Ga[ON(H)O]H(THF)), but with a significant shift for the N−H resonance. Such hydrogen‐bonding effects may also play an important role in catalysis, for example by allowing for the carbonyl substrate to associate with the catalyst organising an insertion transition state.^[55]^

A variable time normalization analysis was applied to determine the order of the reaction using **4** **a** as a catalyst.^[56]^ The fitting supports a rate equation that is pseudo zero order in the concentration of ketones and first order in the concentration of catalyst and pinacol borane. This suggests that the reaction of a gallium alkoxide with pinacol borane is the rate limiting step in the catalytic cycle (i. e. that carbonyl insertion is faster).

We next turned our attention to the catalytic hydroboration of CO_2_ (Scheme 6). The carbon‐oxygen double bond of carbon dioxide is considered among one of the most inert C=O bonds and examples of main group catalysed reduction of CO_2_ are scarce.[^7^, ^8^, ^9^, ^10^, ^11^] The hydroboration of CO_2_ was attempted with **4** **a** (1.0 mol%), involving one equivalent of pinacolborane and 2 bar CO_2_ at room temperature in C_6_D_6_. In accordance with literature reported chemical shifts,^[8]^ the formation of MeOBpin (with ^1^H NMR resonances at 1.04 ppm and 3.50 ppm, a ^11^B NMR resonances at 22.67 ppm and ^13^C NMR resonances at 24.75, 52.41, 82.52 ppm) and O(Bpin)_2_ (^1^H NMR singlet resonance at 1.01 ppm, ^11^B NMR resonance at 21.76 ppm and ^13^C NMR at 24.67, 82.94 ppm) were observed. The reaction reached full conversion within 38 h, which gives a turnover frequency of 2.6 h^−1^. Of the known main‐group metal hydrides used for the catalytic hydroboration of carbon dioxide, our system is only the second example that allows for this transformation to be carried out at room temperature.[^7^, ^8^, ^9^, ^10^, ^11^] Aldridge's gallium hydride shows a comparable turnover frequency of 2.4 h^−1^, albeit at 60 °C, while our catalyst's performance is comparable to Jones’ germanium catalyst (TOF=2.1 h^−1^), yet somewhat slower than the analogous tin system (TOF=14.5 h^−1^) at room temperature.[^8^, ^9^]

## Conclusion

In summary, we have reported two types of novel gallium‐hydride complexes including a unique bimetallic system which exhibits fluxional behaviour in solution. Both systems can be employed as precursors to alkoxide bridged bimetallic compounds which are competent catalysts for the catalytic hydroboration of aldehydes, ketones and carbon dioxide under mild conditions.

Deposition Numbers 2103522 (for **1**), 2103523 (for **2**), 2103524 (for **3**), 2103525 (for **3** ⋅ 1.5(BH_3_:py)), 2103526 (for **4** **a** ⋅ 4 C_6_D_6_), 2103527 (for **4** **b** ⋅ 4tol), 2103528 (for **5** ⋅ 1.5tol), 2103529 (for **6** **a** ⋅ 3tol), 2103530 (for **6** **b** ⋅ 3tol), 2103531 (for **6** **c** ⋅ 2 C_6_D_6_), 2103532 (for **6** **d** ⋅ 2 C_6_D_6_), 2103533 (**6** **e** ⋅ 2CDCl_3_), 2103533 (for **7** **a** ⋅ 4CDCl_3_), 2103535 (**7** **b** ⋅ 3 C_6_D_6_), 2103536 (for **7** **c** ⋅ 2 C_6_D_6_), 2103537 (for **7** **d** ⋅ 2 C_6_D_6_) and 2103537 (for **7** **e** ⋅ 2CDCl_3_) contain the supplementary crystallographic data for this paper. These data are provided free of charge by the joint Cambridge Crystallographic Data Centre and Fachinformationszentrum Karlsruhe Access Structures service.

## Conflict of interest

The authors declare no conflict of interest.

## Supporting information

As a service to our authors and readers, this journal provides supporting information supplied by the authors. Such materials are peer reviewed and may be re‐organized for online delivery, but are not copy‐edited or typeset. Technical support issues arising from supporting information (other than missing files) should be addressed to the authors.

Supporting Information
