# Supplementary material for: Pincer‐Supported Gallium Complexes for the Catalytic Hydroboration of Aldehydes, Ketones and Carbon Dioxide
Source: Chemistry. 2021 Oct 27;27(69):17379–85. doi: 10.1002/chem.202103009 (PMC9297891; doi:10.1002/chem.202103009)
Supplement: Supplementary file 1 — Supporting Information [file CHEM-27-17379-s001.pdf]

Chemistry–A European Journal

Supporting Information

Pincer-Supported Gallium Complexes for the Catalytic Hydroboration of Aldehydes, Ketones and Carbon Dioxide

Lingyu Liu, Siu-Kwan Lo, Cory Smith, and Jose M. Goicoechea*

Contents

1. General considerations and starting materials 3

2. Experimental details 4

2.1. Preparation of *N*,*N*-bis-(3,5-di-*tert*-butyl-2-phenol)amine (H_3(ONO)) 4

2.2. Preparation of Ga[ON(H)O]Cl(py) (**1**) 5

2.3. Preparation of Ga[ON(H)O]Br(THF) (**2**) 7

2.4. Preparation of HGa(THF)[ON(H)O]·BH_3:py (**3**·BH_3:py) 9

2.5. Preparation of {Ga[ON(H)O](OCH_2CH_3)}$_2$ (**4a**) 12

2.6. Preparation of {Ga[ON(H)O](OCH_2Cy)}$_2$ (**4b**) 15

2.7. Preparation of H_2Ga[μ^2-ON(H)O]Ga[ON(H)O] (**5**) 17

2.8. Preparation of Ga[ON(H)O]H(THF) (**3**) 33

2.9. General synthesis and characterisation data for {Ga[ON(H)O](OC(H)R^1R^2)}$_2$ (**6**, **7**) 39

2.9.1. Preparation of {Ga[ON(H)O](OCH_2Ph)}$_2$ (**6a**) 39

2.9.2. Preparation of {Ga[ON(H)O](OCH_2CHMe_2)}$_2$ (**6b**) 42

2.9.3. Preparation of {Ga[ON(H)O]($OCH_2C_6H_4$OMe)}$_2$ (**6c**) 44

2.9.4. Preparation of {Ga[ON(H)O]($OCH_2C_6H_4$-4-Br)}$_2$ (**6d**) 45

2.9.5. Preparation of {Ga[ON(H)O]($OCH_2C_6H_2Me_3$)}$_2$ (**6e**) 47

2.9.6. Preparation of {Ga[ON(H)O](OCH($Ph)_2$)}$_2$ (**7a**) 48

2.9.7. Preparation of {Ga[ON(H)O](OCH($Me)_2$)}$_2$ (**7b**) 50

2.9.8. Preparation of {Ga[ON(H)O][OCH(CH_3)(C_6H_4-4-OMe)]} (**7c**) 51

2.9.9. Preparation of {Ga[ON(H)O][OCH(CH_3)(C_6H_4-4-Br)]}$_2$ (**7d**) 53

2.9.10. Preparation of {Ga[ON(H)O]($OCHC_8H_8$)}$_2$ (**7e**) 55

2.9.11. Preparation of {Ga[ON(H)O][OCH(CH_3)(C_6H_4-4-NO_2)]}$_2$ (**7f**) 57

3. Catalytic hydroboration of aldehydes and ketones 60

3.1. Catalytic hydroboration of aldehydes 60

3.2. Catalytic hydroboration of ketones 63

3.2.1. Control experiment − hydroboration of ketones without catalyst 64

3.2.2. Selected NMR spectra for the catalytic hydroboration of 4′-nitroacetophenone 65

3.2.3. Concentration monitoring of hydroboration of 4′-nitroacetophenone with catalyst **4a** 66

3.2.4. Concentration monitoring of hydroboration of 4′-nitroacetophenone with three different catalysts 66

3.2.5. Inhibition of ketone hydroboration by tetramethylethylenediamine (TMEDA) 69

3.3. Selected data for aldehyde and ketone hydroboration products 70

3.3.1. $PhCH_2$OBpin: product from hydroboration of benzaldehyde. 70

3.3.2. 4-Br-$C_6H_4CH_2$OBpin: product from hydroboration of 4-bromobenzaldehyde . 70
3.3.3. 4-MeO-$C_6H_4CH_2$OBpin: product from hydroboration of 4-methoxybenzaldehyde 70
3.3.4. 2,4,6-$Me_3C_6H_2CH_2$OBpin: product from hydroboration of mesitaldehyde....... 70
3.3.5. 4-FC_6H_4-CH_2OBpin: product from hydroboration of 4-fluorobenzaldehyde.... 70
3.3.6. 4-Me-$C_6H_4CH_2$OBpin: product from hydroboration of 4-methylbenzaldehyde 71
3.3.7. (4-Br-C_6H_4)(CH_3)C(H)OBpin: product from hydroboration of 4'-bromoacetophenone 71
3.3.8. (4-MeO-C_6H_4)(CH_3)C(H)OBpin: product from hydroboration of 4'-methoxy-acetophenone 71
3.3.9. $(CH_3)_2$C(H)OBpin: product from hydroboration of acetone 71
3.3.10. $(CH_3)_2$C(H)CH_2OBpin: product from hydroboration of isobutyraldehyde 72
3.3.11. (4-NO_2-C_6H_4)C(H)(CH_3)OBpin: product from hydroboration of 4'-nitroacetophenone 72
3.3.12. (4-I-C_6H_4)C(H)(CH_3)OBpin: product from hydroboration of 4'-iodoacetophenone 72
3.3.13. (4-Cl-C_6H_4)C(H)(CH_3)OBpin: product from hydroboration of 4'-chloroacetophenone 72
3.4. Kinetic studies of hydroboration of aldehydes and ketones 73
3.5. A simplified proposed mechanism for hydroboration of aldehydes and ketones 76
3.6. Simulation studies of hydroboration of ketones 76
4. Catalytic hydroboration of carbon dioxide 78
4.1. NMR spectra for catalytic hydroboration of carbon dioxide 79
5. X-ray crystallographic studies 81
6. References 105

1. General considerations and starting materials

General synthetic methods. All reactions were performed under an inert atmosphere of argon using standard Schlenk-line or glovebox techniques (MBraun UNIlab glovebox, maintained at < 0.1 ppm H_2O and < 0.1 ppm O_2). *n*-Heptane (Fluka Analytical) was degassed with nitrogen before use. Hexane (Sigma Aldrich, HPLC grade), pentane (Sigma Aldrich, HPLC grade), and toluene (Sigma Aldrich, HPLC grade), benzene (Sigma Aldrich, HPLC grade) were purified using an MBraun SPS-800 solvent system. Tetrahydrofuran (THF; Sigma Aldrich, ≥99.9%) was distilled over a sodium metal/benzophenone mixture and degassed via freeze-pump-thaw method. Pyridine (Sigma Aldrich, HPLC grade) was distilled over CaH_2. Chloroform (Sigma Aldrich, HPLC grade), C_6D_6 (Sigma Aldrich, 99.6%), toluene-d_8 (Sigma Aldrich, 99.6%) and $CDCl_3$ (Fluorochem Ltd, 99.8%) were freeze-pump-thaw degassed three times, and stored over activated 3 Å molecular sieves. THF-d_8 (Euriostop, 99.5%) and triethylamine (Alfa Aesar, 99%) were dried over CaH_2 and vacuum distilled before use. All dry solvents were stored over activated 3 Å molecular sieves under argon in gas-tight ampoules. Potassium hydride (Sigma Aldrich, 30% wt dispersion in mineral oil) was washed with hexane and then dried *in vacuo* before use. 3,5-Di-*tert*-butylcatechol (Alfa Aesar, 99%) was used as received without further purification. *N,N*-bis-(3,5-di-*tert*-butyl-2-phenol)amine were prepared via a previously reported synthesis with modification.[1] Liquid aldehyde, ketone and alcohol substrates were dried over activated 3 Å molecular sieves and freeze-pump-thaw degassed prior to use. All other reagents were used as received.

Additional characterisation techniques. ^1H, ^{13}C, ^{11}B NMR spectra and two-dimensional experiments (NOESY, ROSY, HSQC, HMBC) were conducted in J. Young's NMR tubes on either a Bruker Avance III HD nanobay NMR equipped with a 9.4T magnet (^1H 400.2 MHz, ^{11}B 128.4 MHz, ^{13}C 100.6 MHz, ^{19}F 376.5 MHz), a Bruker Avance III NMR equipped with a 11.75 T, magnet (^1H 499.9 MHz, ^{11}B 160.4 MHz, ^{13}C 125.7 MHz, ^{19}F 470.4 MHz), or a Bruker

Avance NMR equipped with a 11.75 T magnet and a ^{13}C detect cryoprobe (^{1}H 500.3 MHz, ^{13}C 125.8 MHz) at 298 K unless otherwise stated. Chemical shifts (δ) were referenced to internal solvent resonances. Data were processed using MestReNova or TopSpin software.[2] Coupling constants (*J*) are reported in Hertz (Hz). The following abbreviations are used to define multiplets: s (singlet), d (doublet), t (triplet), q (quadruplet), sept. (septet), dd (doublet of doublets), m (multiplet), br s (broad signal).

Elemental analyses were carried out by Elemental Microanalyses Ltd. (Devon, U.K.). Samples (approx. 10 mg) were submitted in sealed Pyrex ampoules.

2. Experimental details

2.1. Preparation of *N*,*N*-bis-(3,5-di-*tert*-butyl-2-phenol)amine (H_3(ONO))

N,*N*-bis-(3,5-di-*tert*-butyl-2-phenol)amine was prepared via a modified literature procedure.[1] 3,5-Di-*tert*-butylcatechol (25 g, 112.5 mmol) and an ammonia solution (25%, 15 ml, 201 mmol) were stirred in a heptane (30 ml) at room temperature for 10 days, forming a white precipitate. The filtrate was removed with a filter cannula and the remaining solid was washed with pentane (3×15 mL) yielding *N*,*N*-bis-(3,5-di-*tert*-butyl-2-phenol)amine as a white solid (16g, 37.6 mmol; 66.9% yield). ^{1}H NMR (400 MHz, C_6D_6): δ (ppm) 7.22 (d, $^{4}J_{H-H}$ = 2.3 Hz, 2H; Ar-*H*), 6.77 (d, $^{4}J_{H-H}$ = 2.2 Hz, 2H; Ar-*H*), 5.04 (s, 2H; O*H*), 4.59 (s, 1H; N*H*), 1.53 (s, 18H; $C(CH_3)_3$), 1.21 (s, 18H; $C(CH_3)_3$).

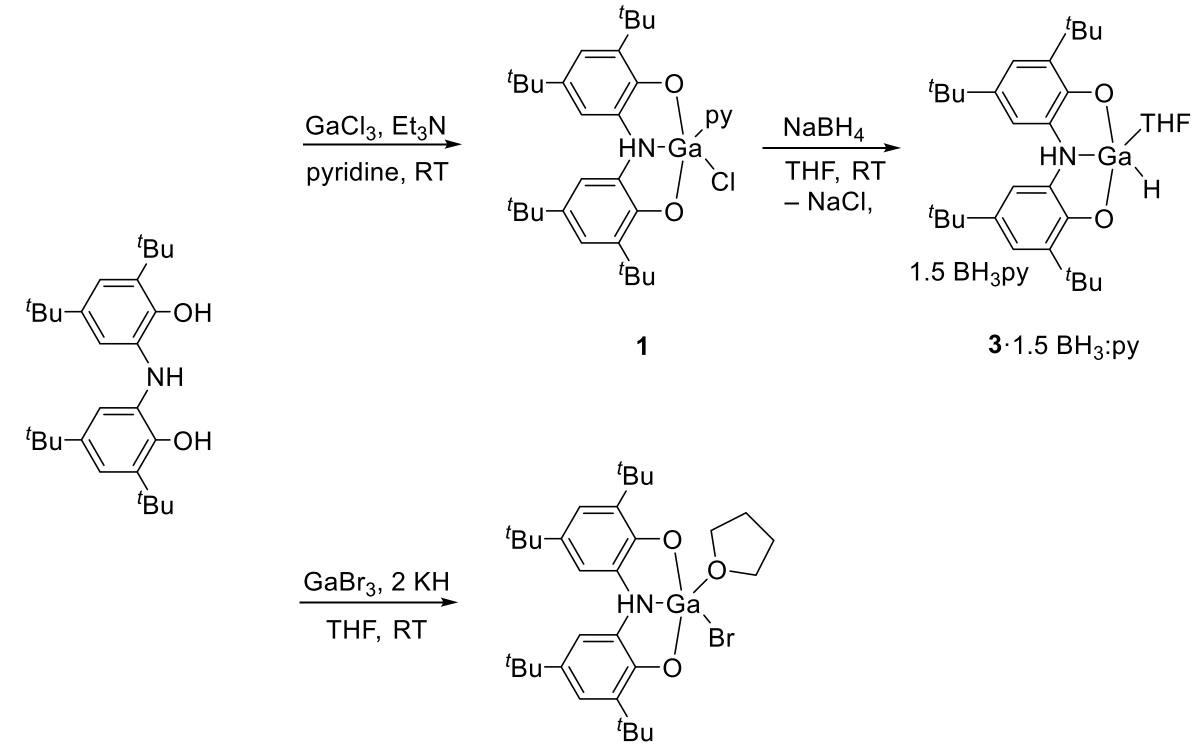

Scheme S1. Synthesis of compounds **1**, **2**and **3**·1.5 BH_3:py.

2.2. Preparation of Ga[ON(H)O]Cl(py) (**1**)

H_3(ONO) (850mg, 2.0 mmol) and $GaCl_3$ (350 mg, 2.0 mmol) were dissolved in pyridine (25 mL). The solution turned yellow-orange. Triethylamine (0.5 mL, 3.1 mmol) was then added to the solution, producing a viscous orange-brown solution. The solution was stirred for two hours at room temperature. All volatiles were removed under vacuum and the product was extracted into hot hexane, followed by filtration. The solution was then reduced to dryness under vacuum and product was purified by recrystallization from a hot hexane solution, affording **1** as a compositionally pure white solid (1050 mg, 1.73 mmol, 86.5% yield). Elemental analysis calculated for $C_{33}H_{46}ClGaN_2O_2$: C, 65.20%; H, 7.63%; N, 4.61%; found: C, 65.12%; H, 7.73%; N, 4.78%. ^{1}H NMR (400 MHz, C_6D_6): δ (ppm) 9.16–9.10 (m, 2H; py-*H*), 7.41 (d, $^4J_{H\text{–}H}$ = 2.3 Hz, 2H; Ar-*H*), 7.22 (d, $^4J_{H\text{–}H}$ = 2.3 Hz, 2H; Ar-*H*), 6.75 (t, $^3J_{H\text{–}H}$ = 7.7 Hz, 1H; py-*H*), 6.52 (t, $^3J_{H\text{–}H}$ = 6.7 Hz, 2H; py-*H*), 4.61 (s, 1H; N*H*), 1.53 (s, 18H; C(CH_3)$_3$), 1.38 (s, 18H; C(CH_3)$_3$).

$^{13}C\{^{1}H\}$ NMR (101 MHz, C_6D_6): δ (ppm) 154.76 (Ar-*C*), 147.67 (py-*C*), 139.95 (py-*C*), 138.73 (Ar-*C*), 137.26 (Ar-*C*), 130.87 (Ar-*C*), 124.90 (py-*C*), 122.90 (Ar-*C*), 120.76 (Ar-*C*), 35.56 (*C*(CH₃)₃), 34.48 (*C*(CH₃)₃), 31.98 (C(*C*H₃)₃), 29.79 (C(*C*H₃)₃).

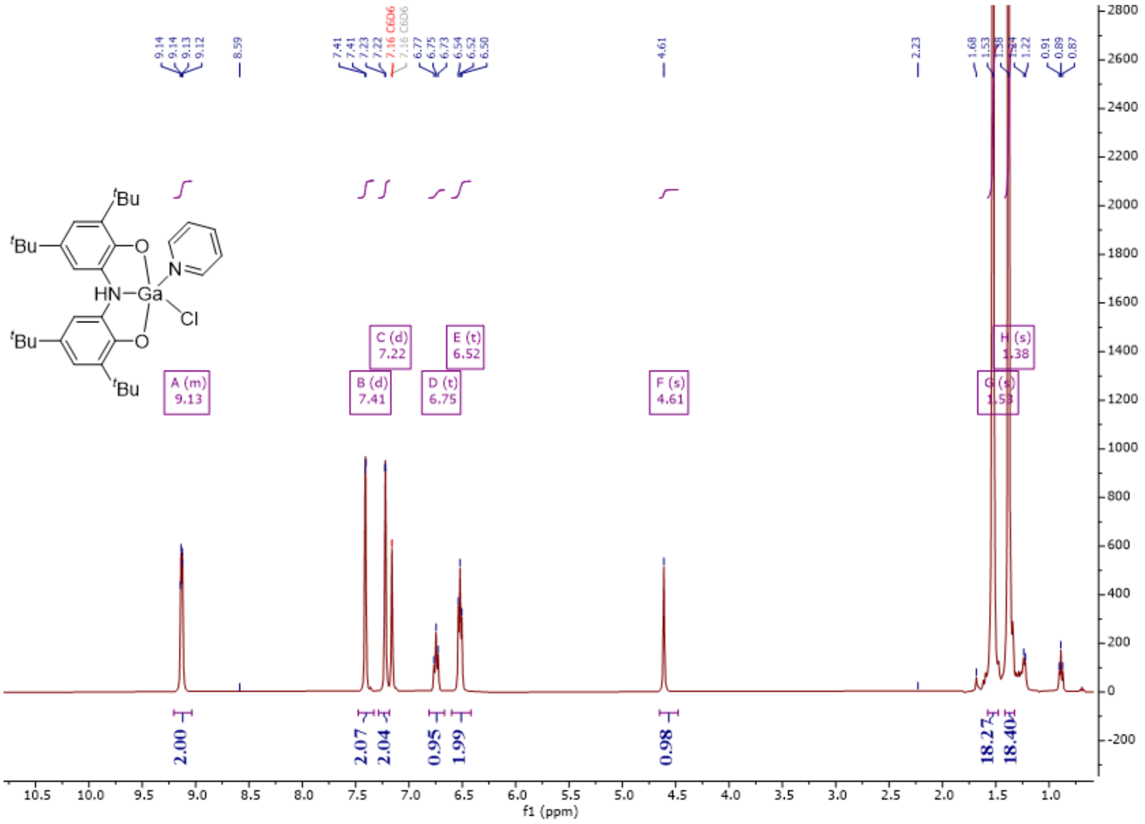

Figure S1. ^{1}H NMR spectrum for compound **1** in C_6D_6.

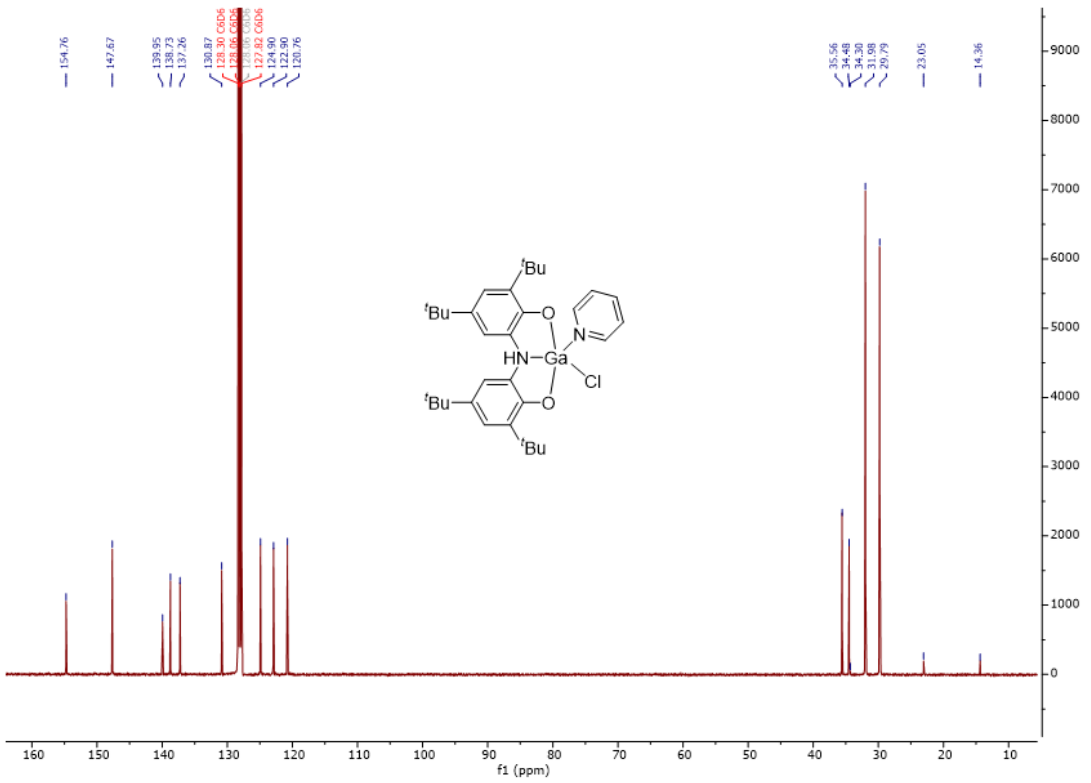

Figure S2. ^{13}C NMR spectrum for compound **1** in C_6D_6.

2.3. Preparation of Ga[ON(H)O]Br(THF) (**2**)

H_3(ONO) (260 mg, 0.61 mmol), $GaBr_3$ (189 mg, 0.61 mmol) and potassium hydride (61 mg, 1.53 mmol) were dissolved in THF (20 mL), affording a grey solution and a white precipitate. This solution was sonicated for three hours and then filtered, resulting in a pale orange solution. All volatiles were removed *in vacuo*, affording a pale orange solid (292 mg, 0.45 mmol, 74% yield). Crystals suitable for single crystal X-ray crystallography were grown from a hexane solution at 50 °C. Elemental analysis calculated for $C_{32}H_{49}NO_3GaBr$: C, 59.55%; H, 7.65%; N, 2.17%; found: C, 59.00%; H, 7.73%; N, 1.98%. ^{1}H NMR (400 MHz, C_6D_6): δ (ppm) 7.38 (d, $^4J_{H\text{–}H}$ = 2.4 Hz, 2H; Ar-*H*), 7.13 (d, $^4J_{H\text{–}H}$ = 2.4 Hz, 2H; Ar-*H*), 4.56 (br, 1H; N*H*), 3.96 (br, 4H; OCH_2), 1.55 (s, 18H; C(CH_3)$_3$), 1.38 (br, 4H; OCH$_2$CH_2), 1.35 (s, 18H; C(CH_3)$_3$). ^{13}C{^{1}H} NMR (101 MHz, C_6D_6): δ (ppm) 154.40 (Ar-*C*), 138.96 (Ar-*C*), 137.43 (Ar-*C*), 130.51 (Ar-*C*),

123.16 (Ar-*C*), 120.45 (Ar-*C*), 69.86 (O*C*H_2), 35.60 (*C*(CH_3)$_3$), 34.46 (*C*(CH_3)$_3$), 31.92 (C(*C*H_3)$_3$), 29.71 (C(*C*H_3)$_3$), 25.46 (OCH_2*C*H_2).

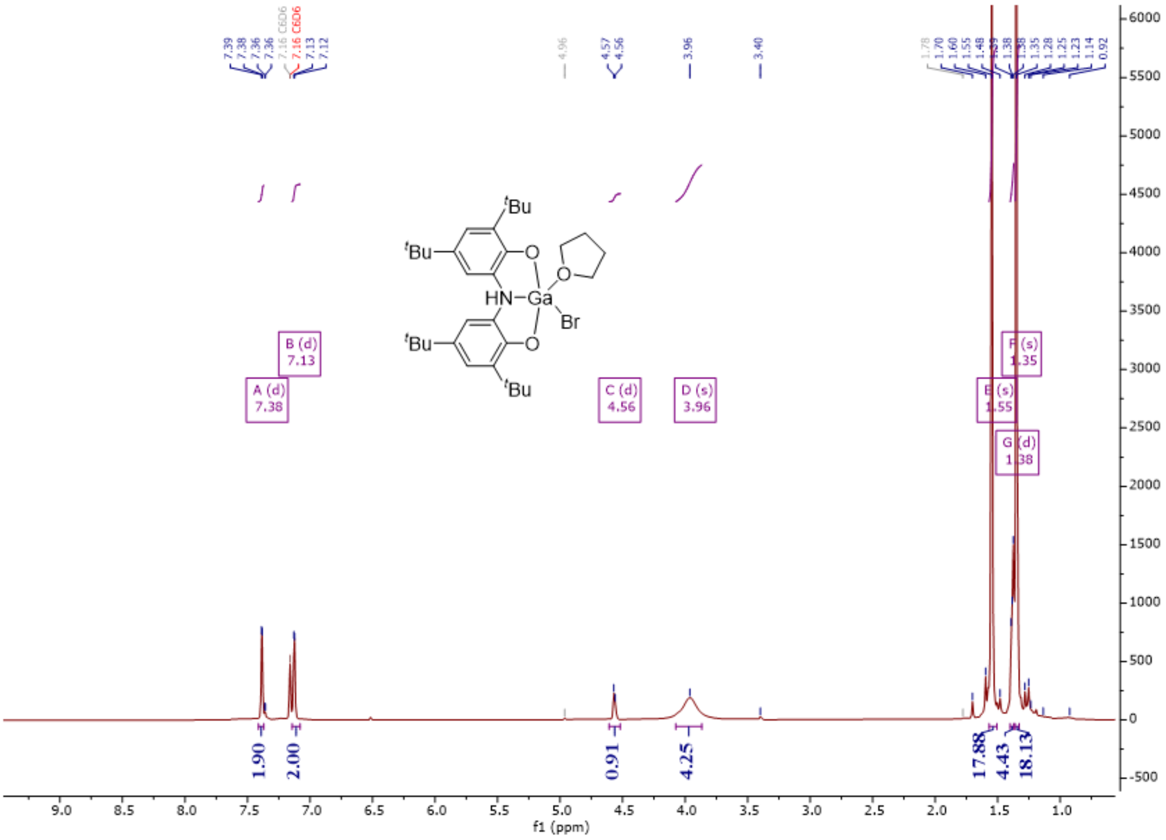

Figure S3. ^{1}H NMR spectrum for compound **2** in C_6D_6.

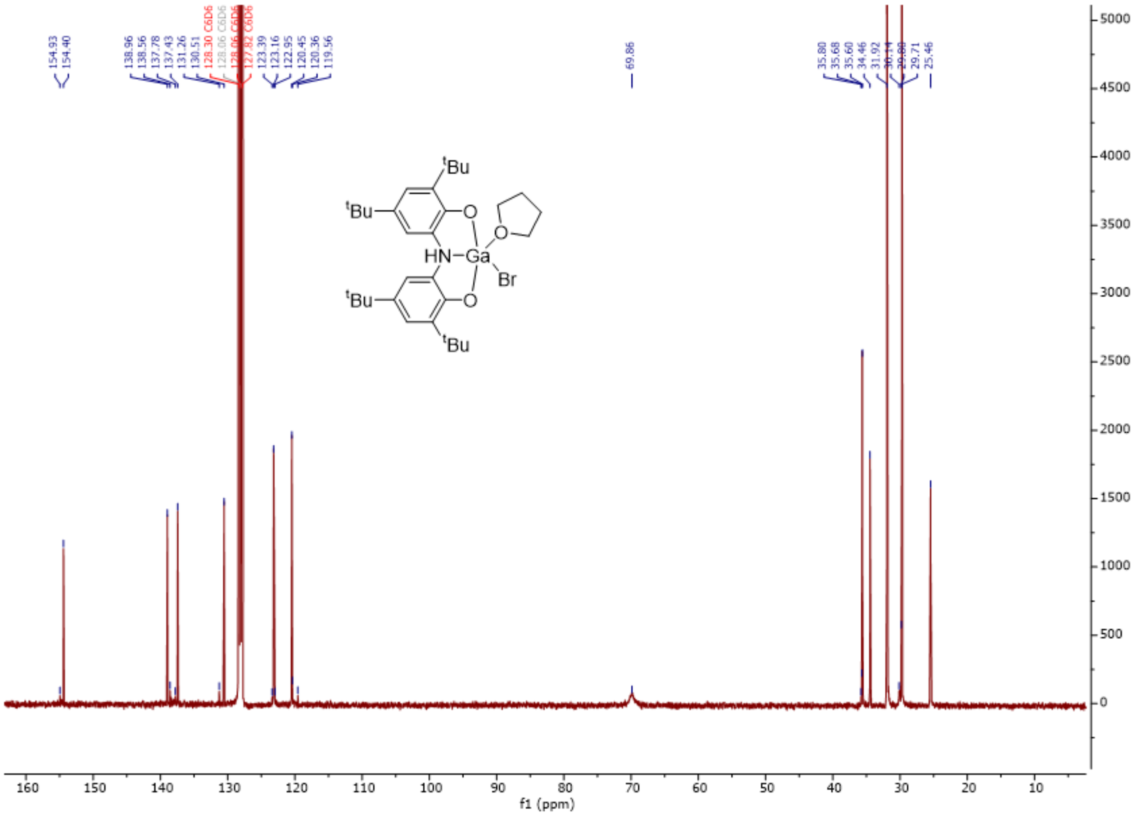

Figure S4. ^{13}C NMR spectrum for compound **2** in C_6D_6.

2.4. Preparation of HGa(THF)[ON(H)O]·BH_3:py (**3**·BH_3:py)

1 (525mg, 0.87mmol) and sodium borohydride (5.2 eq, 170 mg, 4.5 mmol) were dissolved in THF (20 mL) affording a colourless solution. The solution was stirred overnight resulting in the formation of a white precipitate of NaCl. All volatiles were removed *in vacuo*, and the product was extracted into toluene and filtered. The solvent was subsequently removed under a dynamic vacuum, yielding the impure product. The product was purified by recrystallization from concentrated toluene and pentane, yielding the product as a white solid. (360mg, 0.55 mmol, 73.6% yield). Colourless crystals suitable for X-ray diffraction analysis were obtained by layering a concentrated toluene with pentane at 4 °C. Note that this method results in borane-pyridine being present as an impurity, which cannot be removed by washing. Elemental analysis calculated for $C_{39.5}H_{62}B_{1.5}GaN_{2.5}O_3$: C, 67.21%; H, 8.85%; N, 4.96%; found: C, 67.56%; H,

8.89%; N; 4.55%. ^{1}H NMR (400 MHz, C_6D_6): δ (ppm) 8.13 (d, $^{3}J_{H\text{–}H}$ = 5.6 Hz, 2H; py-*H*), 7.35 (d, $^{4}J_{H\text{–}H}$ = 2.4 Hz, 2H; Ar-*H*), 7.20 (d, $^{4}J_{H\text{–}H}$ = 2.4 Hz, 2H; Ar-*H*), 6.52 (t, $^{3}J_{H\text{–}H}$ = 7.7 Hz, 1H; py-*H*), 6.15 (t, $^{3}J_{H\text{–}H}$ = 6.9 Hz, 2H; py-*H*), 5.67 (br, 1H; Ga*H*), 4.01 (s, 1H; N*H*), 3.91 (m, 4H; OCH_2), 3.76–3.11 (m, 3H; BH_3), 1.59 (s, 18H; C(CH_3)$_3$), 1.45 (m, 4H; OCH$_2$CH_2), 1.35 (s, 18H; C(CH_3)$_3$). ^{13}C{^{1}H} NMR (101 MHz, C_6D_6): δ (ppm) 156.11(Ar-*C*), 147.27 (py-*C*),, 137.87(py-*C*), 137.80(Ar-*C*), 137.42 (Ar-*C*), 132.09 (Ar-*C*), 124.66 (py-*C*), 122.61 (Ar-*C*), 120.36 (Ar-*C*), 68.72 (O*C*H$_2$), 35.63 (*C*(CH$_3$)$_3$), 34.36 (*C*(CH$_3$)$_3$), 32.00 (C(*C*H$_3$)$_3$), 29.76 (C(*C*H$_3$)$_3$), 25.53 (OCH$_2$*C*H$_2$). ^{11}B NMR (128 MHz, C_6D_6): δ (ppm) –10.78 (q, $^{1}J_{B\text{–}H}$ = 100.4 Hz).

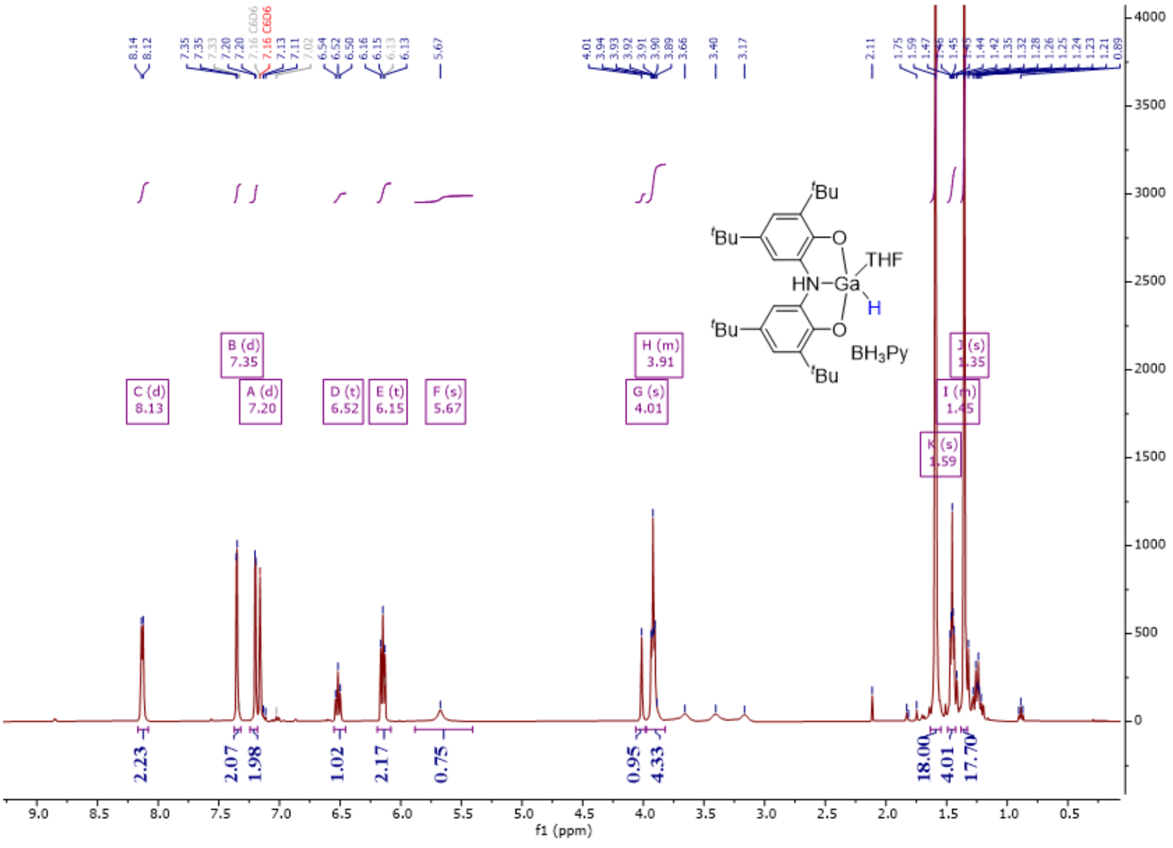

Figure S5. ^{1}H NMR spectrum for compound **3**·BH_3:py in C_6D_6.

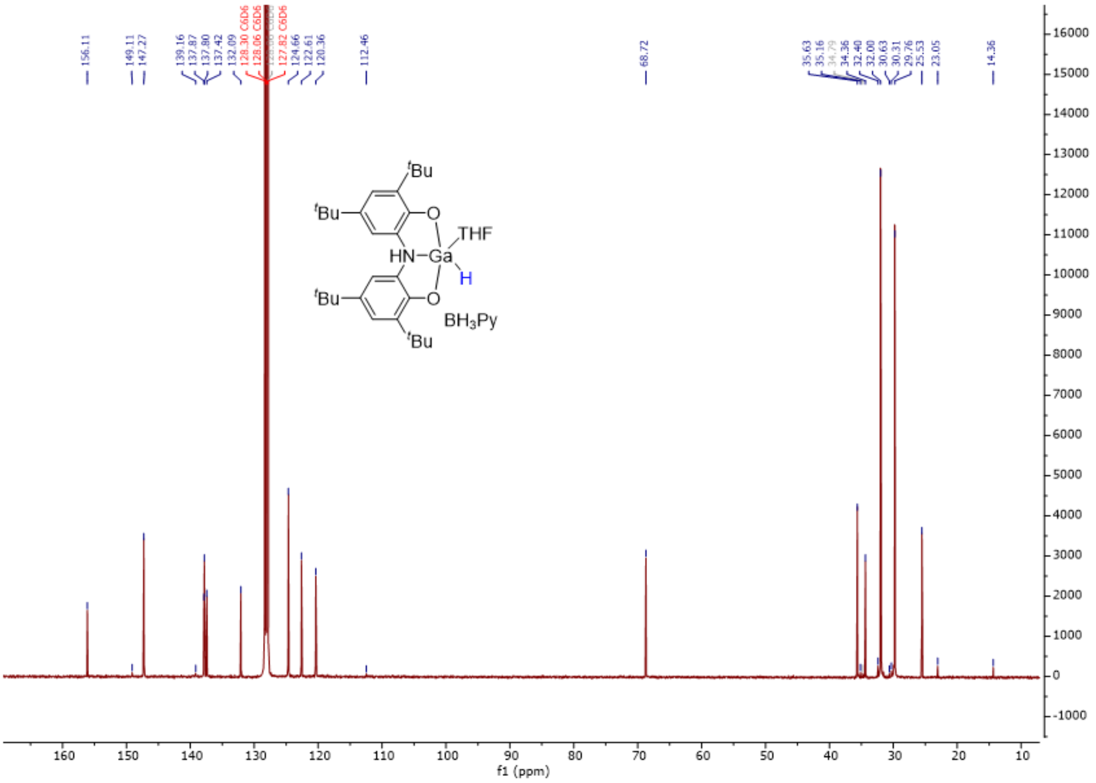

Figure S6. ^{13}C NMR spectrum for compound **3**·BH_3:py in C_6D_6.

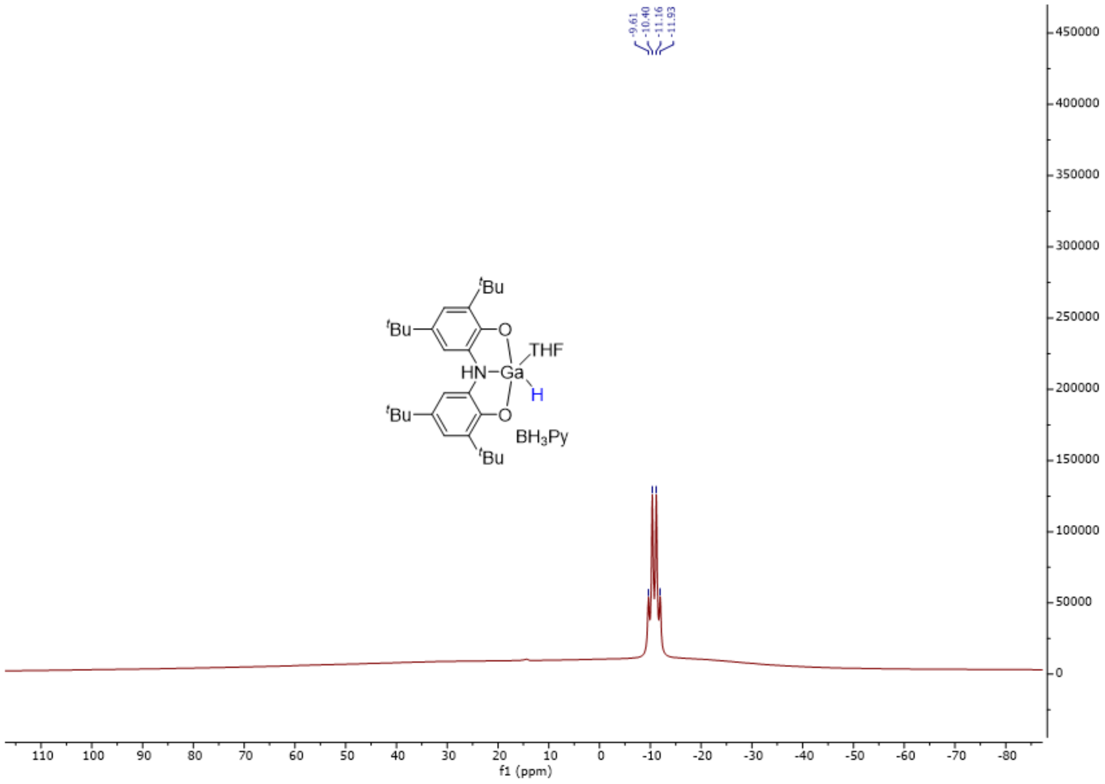

Figure S7. ^{11}B NMR spectrum for compound **3**·BH_3:py in C_6D_6.

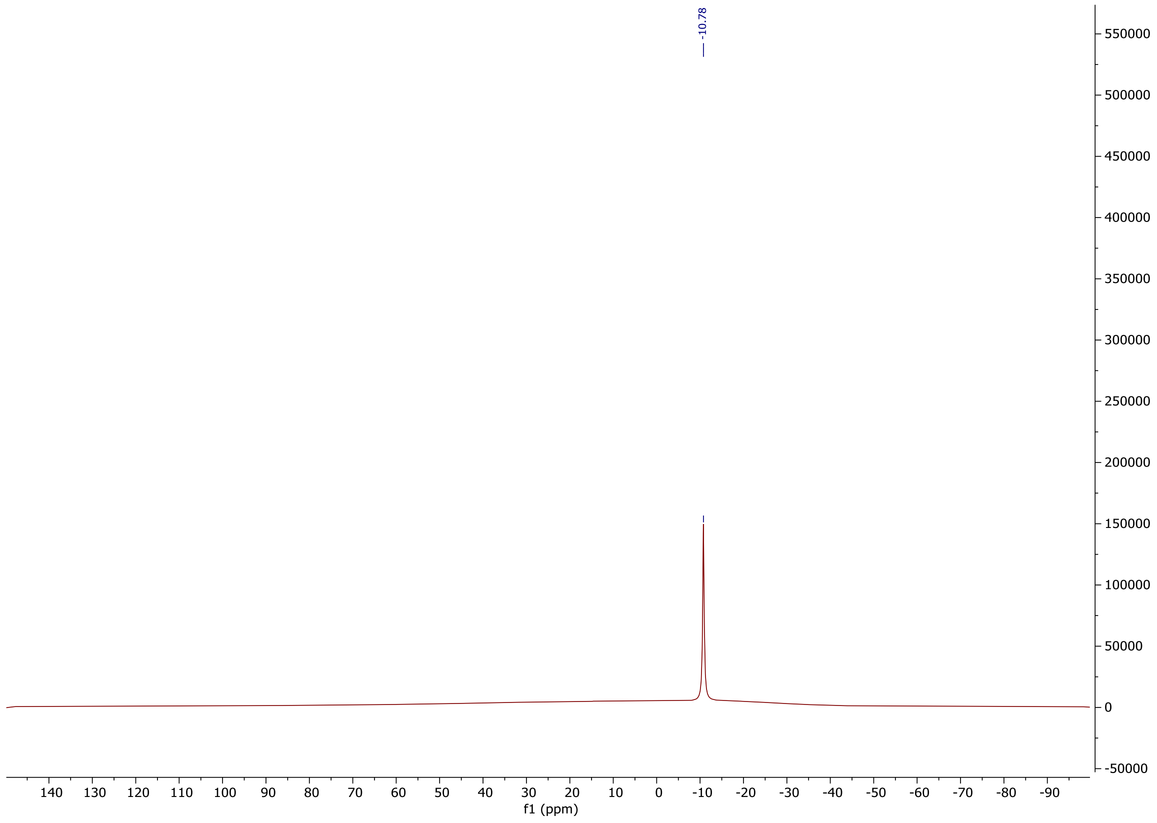

Figure S8. ^{11}B (proton - decoupled) NMR spectrum for compound **3**·BH_3py in C_6D_6.

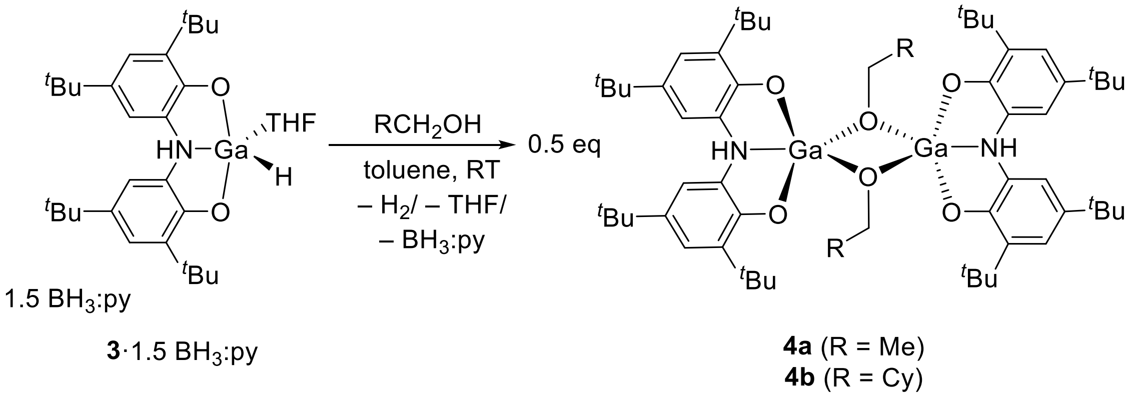

Scheme S2. Synthesis of compounds **4a** and **4b**.

2.5. Preparation of {Ga[ON(H)O](OCH_2CH_3)}$_2$ (**4a**)

3·BH_3:py (2.82 g, 4.28 mmol) was stirred in ethanol (5 mL) overnight yielding a white precipitate. The solution was filtered and the white solid was washed with ethanol (4 × 10 mL) and pentane (1 × 10 mL). The solid was dried *in vacuo*, affording **4a** (1600 mg, 1.49 mmol,

69.5% yield). The product was crystallised as colourless crystals suitable for single crystal X-ray diffraction from a concentrated benzene solution at room temperature. Elemental analysis calculated for $C_{60}H_{92}Ga_2N_2O_6$: C, 66.92%; H, 8.61%; N, 2.60%; found: C, 66.51%; H, 8.73%; N, 2.54%. ^{1}H NMR (500 MHz, C_6D_6): δ (ppm) 7.46 (d, $^{4}J_{H–H}$ = 2.4 Hz, 4H; Ar-*H*), 7.42 (d, $^{4}J_{H–H}$ = 2.4 Hz, 4H; Ar-*H*), 4.70 (s, 2H; N*H*), 4.05 (br, 4H; OC*H*$_2$), 1.40−1.29 (overlapped singlets, 78H; C(C*H*$_3$)$_3$ and CH$_2$C*H*$_3$). ^{13}C{^{1}H} NMR (126 MHz, C_6D_6): δ (ppm) 154.60 (Ar-*C*), 139.00 (Ar-*C*), 138.40 (Ar-*C*), 130.52 (Ar-*C*), 123.43 (Ar-*C*), 119.73 (Ar-*C*), 61.85 (O*C*H$_2$), 35.74 (*C*(CH$_3$)$_3$), 34.49 (*C*(CH$_3$)$_3$), 31.94 (C(*C*H$_3$)$_3$), 29.68 (C(*C*H$_3$)$_3$), 19.07 (OCH$_2$*C*H$_3$).

^{1}H NMR (500 MHz, $CDCl_3$): δ (ppm) 7.56 (d, $^{4}J_{H–H}$ = 2.3 Hz, 4H; Ar-*H*), 7.22 (d, $^{4}J_{H–H}$ = 2.3 Hz, 4H; Ar-*H*), 5.05 (s, 2H; N*H*), 4.23–3.96 (m, 4H; OC*H*$_2$), 1.68−1.25 (overlapped singlets, 78H; C(C*H*$_3$)$_3$ and CH$_2$C*H*$_3$). ^{13}C{^{1}H} NMR (126 MHz, $CDCl_3$): δ (ppm) 154.33 (Ar-*C*), 138.61 (Ar-*C*), 138.13 (Ar-*C*), 129.78 (Ar-*C*), 123.43 (Ar-*C*), 119.51 (Ar-*C*), 61.59 (O*C*H$_2$), 35.52 (*C*(CH$_3$)$_3$), 34.44 (*C*(CH$_3$)$_3$), 31.84 (C(*C*H$_3$)$_3$), 29.44 (C(*C*H$_3$)$_3$), 18.87 (OCH$_2$*C*H$_3$).

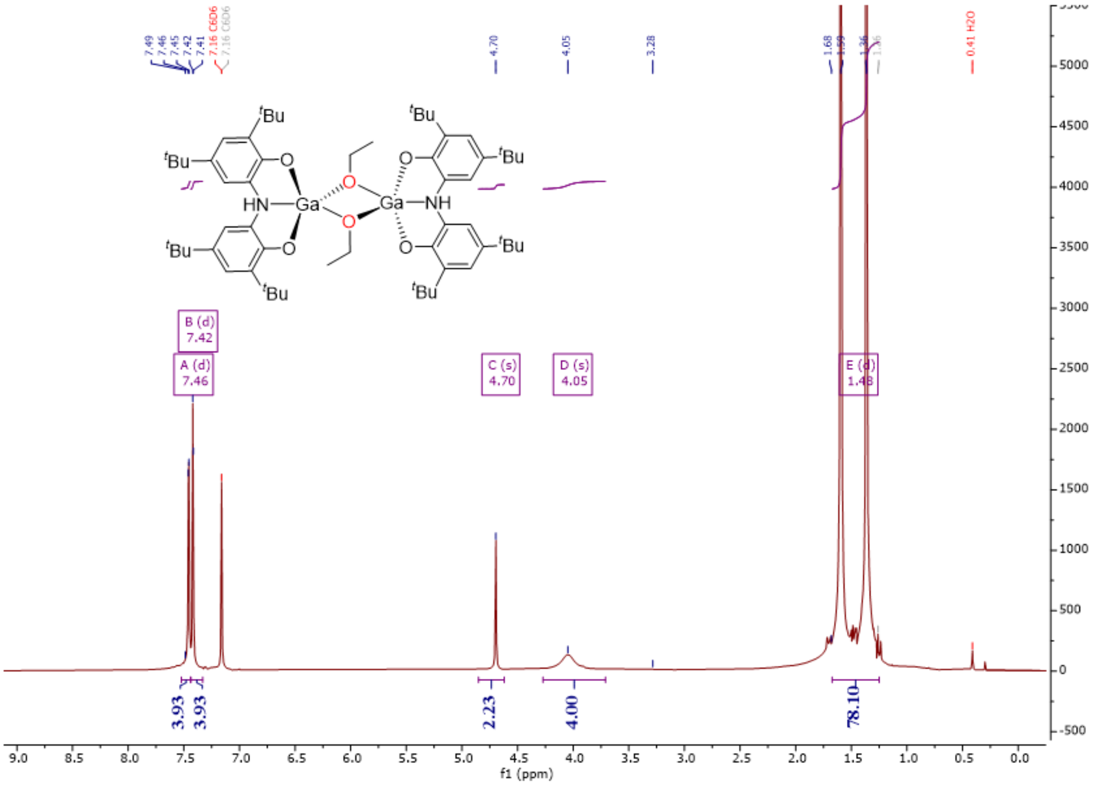

Figure S9. ^{1}H NMR spectrum for compound **4a** in C_6D_6.

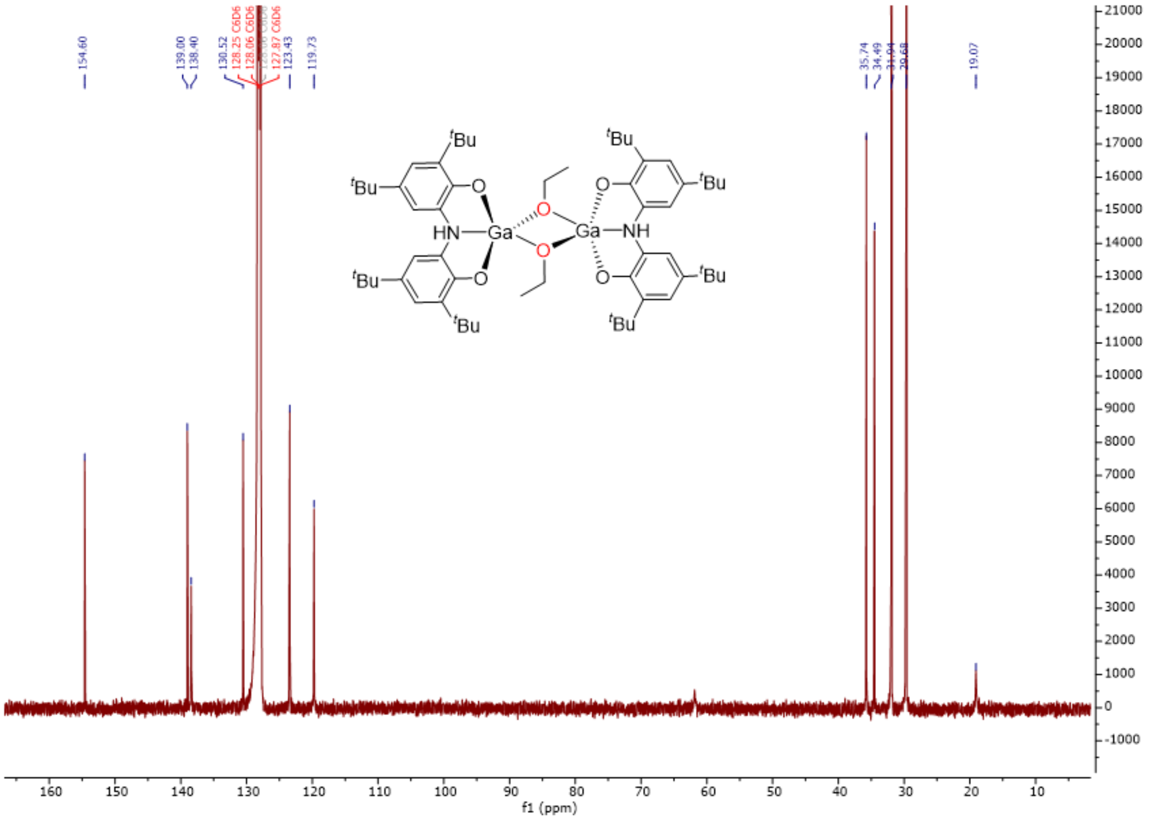

Figure S10. ^{13}C NMR spectrum for compound **4a** in C_6D_6.

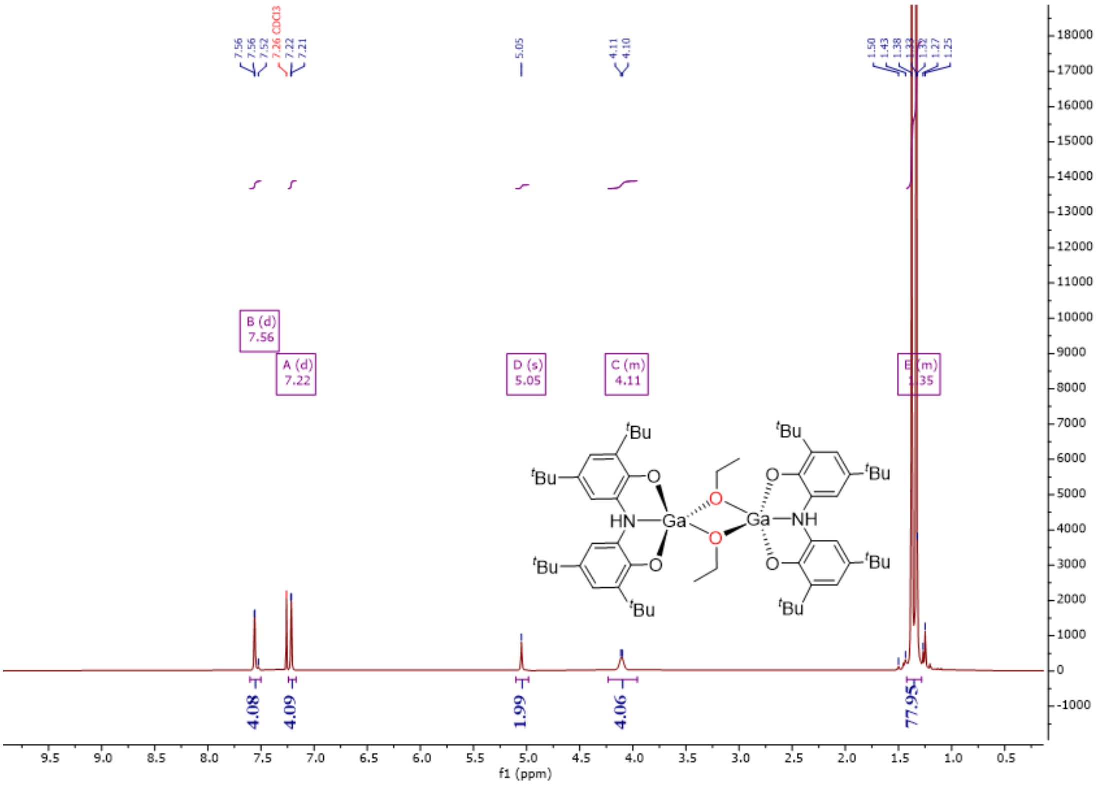

Figure S11. ^{1}H NMR spectrum for compound **4a** in $CDCl_3$.

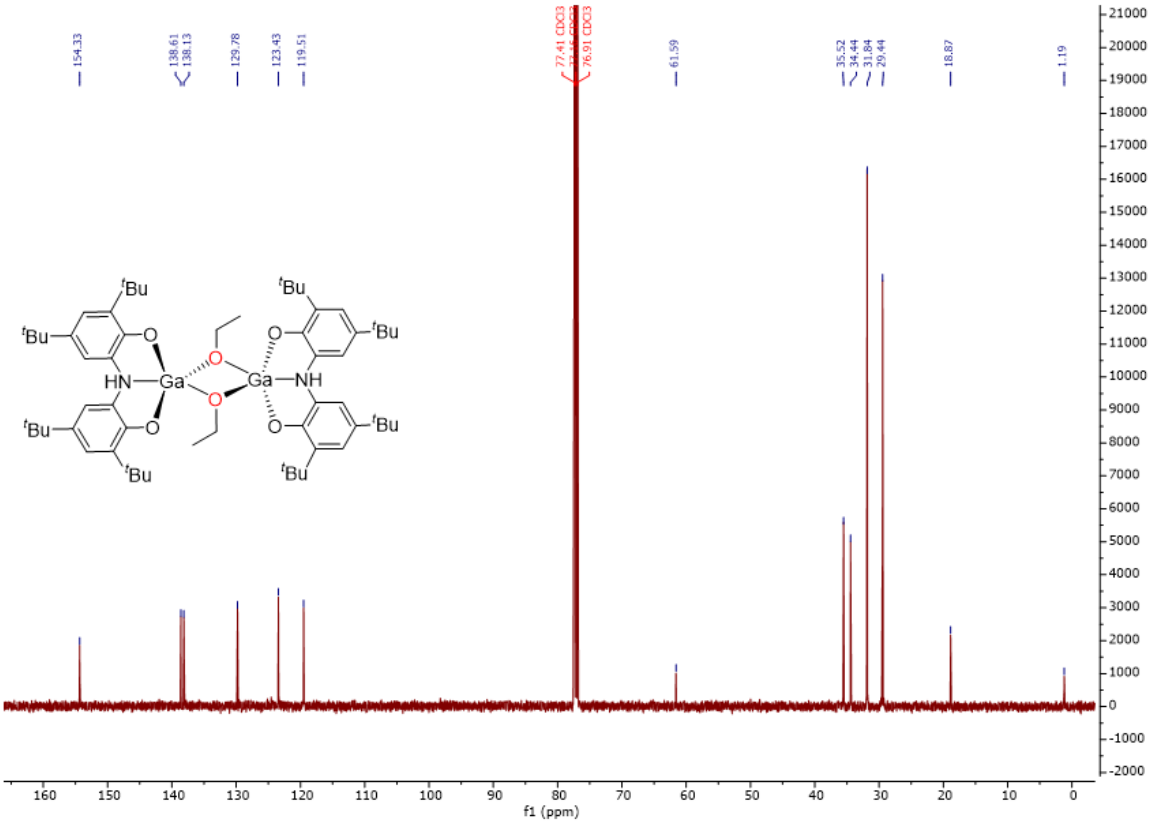

Figure S12. ^{13}C NMR spectrum for compound **4a** in $CDCl_3$.

2.6. Preparation of {Ga[ON(H)O](OCH$_2$Cy)}$_2$ (**4b**)

Cyclohexylmethanol (21 mg, 0.18 mmol) was added into a toluene solution (2.0 mL) of **3** (100 mg, 0.18 mmol) in an ampoule under argon and stirred overnight. The solvent was removed under a dynamic vacuum and the solid remnants were washed with pentane (2 mL), then dried *in vacuo*, affording the desired product as white solid. X-ray quality crystals were obtained from concentrated toluene or chloroform solutions, yielding colourless crystals. (60mg, 0.05mmol, 56%). Elemental analysis calculated for $C_{70}H_{108}N_2O_6Ga_2$: C, 69.31%; H, 8.97%; N, 2.31%; found: C, 69.28%; H, 9.20%; N, 2.49%. ^{1}H NMR (500 MHz, $CDCl_3$): δ (ppm) 7.52 (br, 4H; Ar-*H*), 7.21 (br, 4H; Ar-*H*), 4.97 (s, 2H; N*H*), 3.82 (s, 4H; OC*H*$_2$), 1.82 (s, 2H; OCH$_2$C*H*), 1.54 (s, 8H; OCH$_2$CHC*H*$_2$), 1.39 (s, 36H; C(C*H*$_3$)$_3$), 1.33 (s, 36H; C(C*H*$_3$)$_3$), 1.16 (s, 8H; OCH$_2$CHCH$_2$C*H*$_2$), 0.67 (s, 4H; OCH$_2$CHCH$_2$CH$_2$C*H*$_2$). ^{13}C{^{1}H} NMR (126 MHz, $CDCl_3$): δ (ppm) 154.33 (Ar-*C*), 138.67 (Ar-*C*), 138.04 (Ar-*C*), 129.54 (Ar-*C*), 123.32 (Ar-*C*), 119.39

(Ar-*C*), 71.83 (O*C*H$_2$), 40.01 (OCH$_2$*C*H), 35.51 (*C*(CH$_3$)$_3$), 34.41 (*C*(CH$_3$)$_3$), 31.83 (C(*C*H$_3$)$_3$), 29.98 (OCH$_2$CH*C*H$_2$), 29.47(C(*C*H$_3$)$_3$), 26.74 (OCH$_2$CHCH$_2$*C*H$_2$), 25.66 (OCH$_2$CHCH$_2$CH$_2$*C*H$_2$).

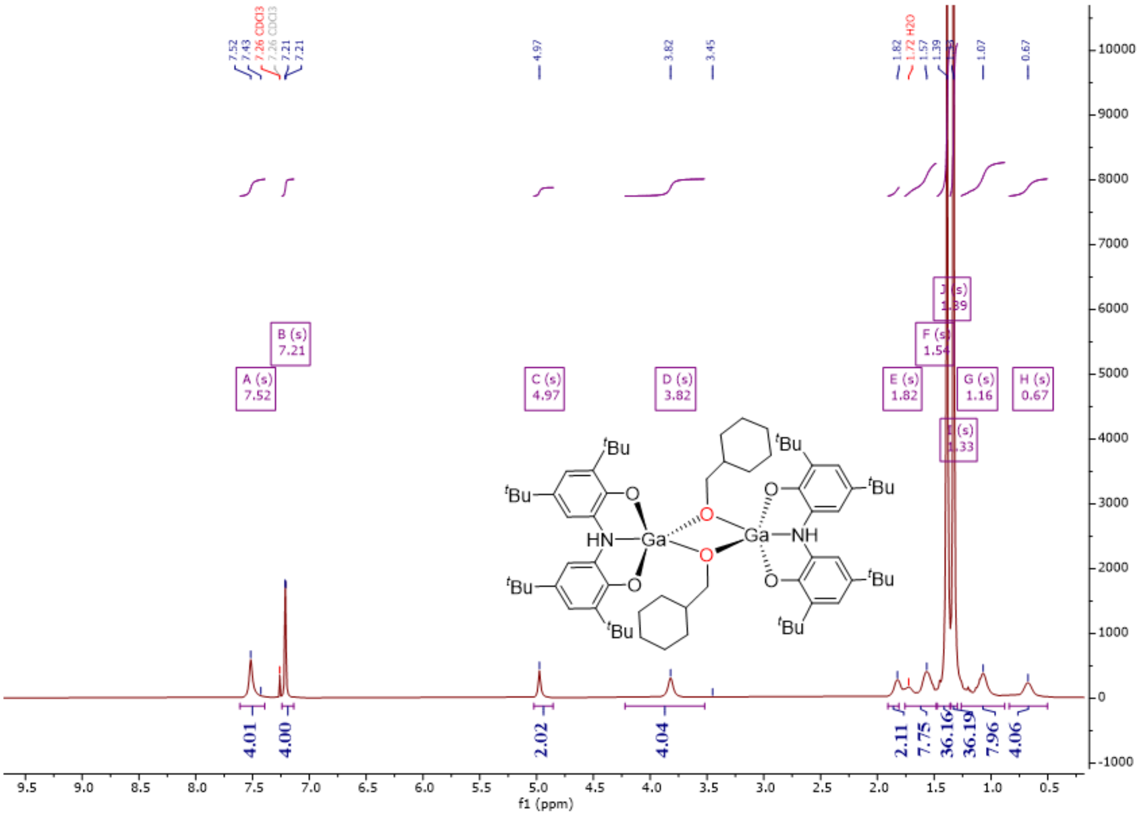

Figure S13. ^{1}H NMR spectrum for compound **4b** in $CDCl_3$.

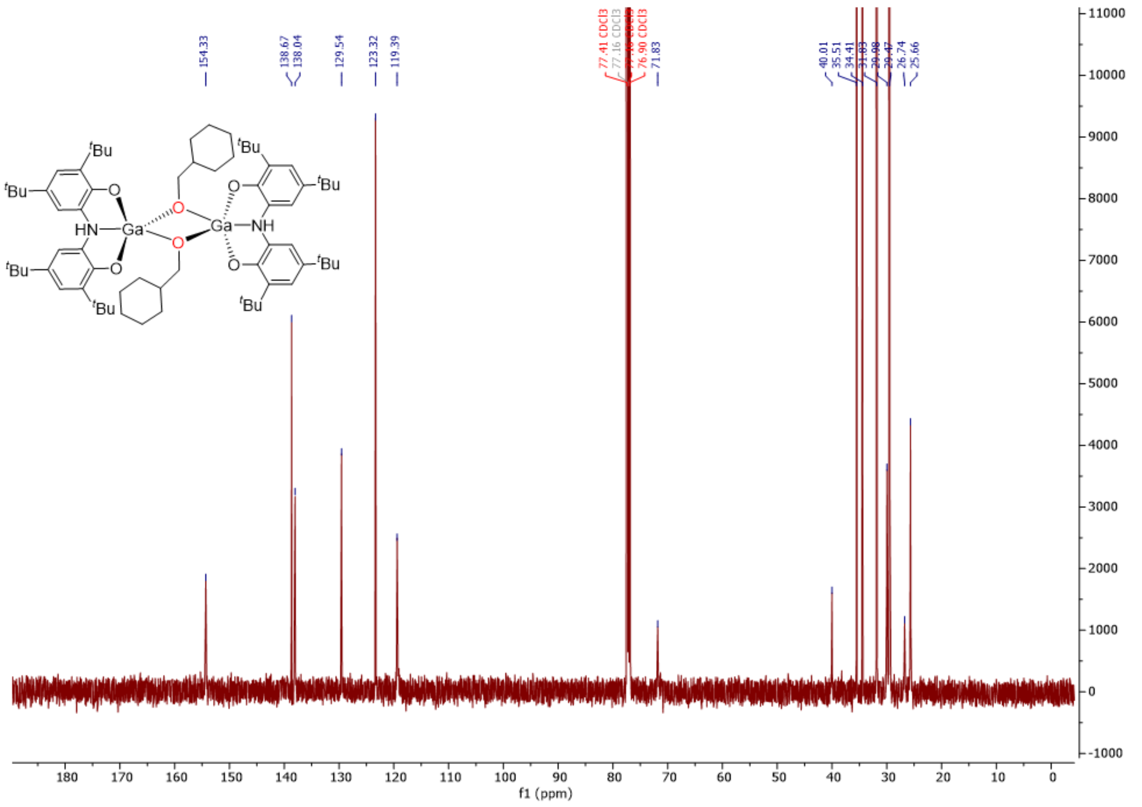

Figure S14. ^{13}C NMR spectrum for compound **4b** in $CDCl_3$.

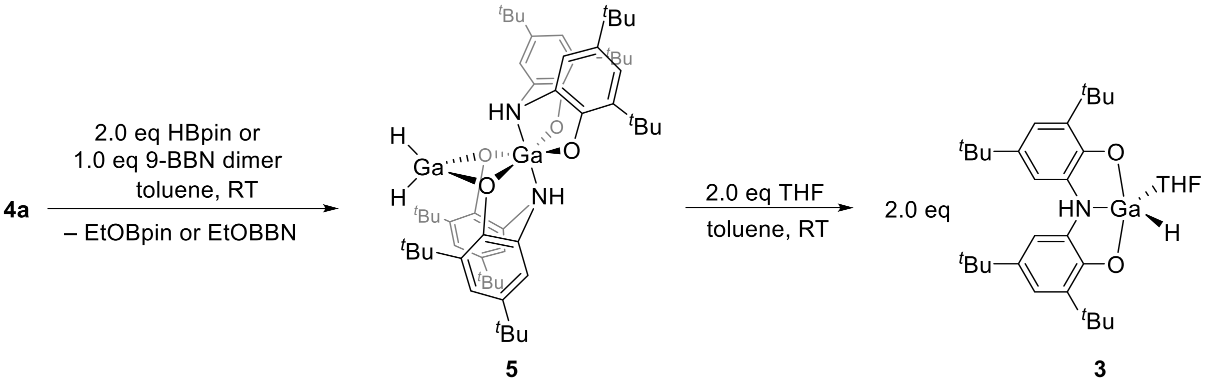

Scheme S3. Synthesis of compounds **3** and **5**.

2.7. Preparation of $H_2Ga[\mu^2\text{-}ON(H)O]Ga[ON(H)O]$ (**5**)

Method 1:

In glovebox, pinacolborane (17 µl, 15.0mg, 0.117mmol) was added to a solution of **4a** (60 mg, 0.056mmol) in toluene (2 mL) using a micro syringe. This afforded a colourless solution after

stirring for 2 mins. After storing at –30 °C in a freezer for 5 days colourless crystals formed. However, using this method we were unable to separate the product from EtOBpin.

Method 2:

4a (180 mg, 0.168 mmol) was stirred in hexane (4 mL) with 9-BBN (9-borabicyclo(3.3.1)nonane) (42.0mg, 0.172 mmol) at room temperature overnight giving a colourless solution. The solution was stored at –30 °C in a freezer for 3 days, affording colourless crystals. The supernatant was decanted and the remaining solid was washed with n-pentane (3 × 2 mL) and subsequently dried *in vacuo* to afford **5** as a white powder (25mg, 0.025 mmol, 15%). The product is soluble in pentane, hexane, benzene, toluene and chloroform. Elemental analysis calculated for $C_{56}H_{84}Ga_2N_2O_4$: C, 68.03%; H, 8.56%; N, 2.83%; found: C, 67.46%; H, 8.50%; N, 2.82%. Room temperature ^{1}H NMR spectra indicate the existence of at least four isomers of compound **5**.

Isomer **5$_{\mathrm{I}}$**:

^{1}H NMR (500 MHz, C_6D_6): δ (ppm) 7.32 (d, $^4J_{H–H}$ = 2.4 Hz, 4H; Ar-*H*), 7.05 (d, $^4J_{H–H}$ = 2.4 Hz, 4H; Ar-*H*), 5.55 (s, 2H; Ga*H*), 4.02 (s, 2H; N*H*), 1.58 (s, 36H; C(CH_3)$_3$), 1.30 (s, 36H; C(CH_3)$_3$). ^{13}C NMR (126 MHz, C_6D_6): δ (ppm) 155.59 (Ar-*C*), 138.96 (Ar-*C*), 138.57 (Ar-*C*), 131.25 (Ar-*C*), 123.50 (Ar-*C*), 118.76 (Ar-*C*), 35.72 (*C*(CH$_3$)$_3$), 34.36 (*C*(CH$_3$)$_3$), 31.84 (C(*C*H$_3$)$_3$), 29.54 (C(*C*H$_3$)$_3$).

Or in toluene-d_8 solution: ^{1}H NMR (500 MHz, toluene-d_8): δ (ppm) 7.28 (d, $^4J_{H–H}$ = 2.4 Hz, 2H; Ar-*H*), 7.06 (d, $^4J_{H–H}$ = 2.4 Hz, 2H; Ar-*H*), 5.53 (s, 1H; Ga*H*), 3.90 (s, 1H; N*H*), 1.54 (s, 18H; C(CH_3)$_3$), 1.31 (s, 18H; C(CH_3)$_3$). ^{13}C NMR (126 MHz, toluene-d_8): δ (ppm) 155.92 (Ar-*C*), 139.18 (Ar-*C*), 138.82 (Ar-*C*), 131.56 (Ar-*C*), 123.79 (Ar-*C*), 119.01 (Ar-*C*), 36.08 (*C*(CH$_3$)$_3$), 34.74 (*C*(CH$_3$)$_3$), 32.20 (C(*C*H$_3$)$_3$), 29.90 (C(*C*H$_3$)$_3$).

Isomer **5**$_{\mathbf{II}}$:

^{1}H NMR (500 MHz, C_6D_6): δ 7.55 (d, $^4J_{H-H}$ = 2.4 Hz, 2H; Ar-*H*), 7.33 (m, 4H; Ar-*H*), 6.81 (d, $^4J_{H-H}$ = 2.4 Hz, 2H; Ar-*H*), 5.94 (s, 1H; Ga*H*), 5.86 (s, 1H; Ga*H*), 5.11 (s, 1H; N*H*), 4.82 (s, 1H; N*H*), 1.43 (s, 18H; C(CH_3)$_3$), 1.32 (s, 18H; C(CH_3)$_3$), 1.25 (s, 18H; C(CH_3)$_3$), 1.24 (s, 18H; C(CH_3)$_3$).

Or in toluene-d_8 solution: ^{1}H NMR (500 MHz, toluene-d_8): δ 7.51 (d, $^4J_{H-H}$ = 2.3 Hz, 2H; Ar-*H*), 7.31 (d, $^4J_{H-H}$ = 2.4 Hz, 2H; Ar-*H*), 7.29 (d, $^4J_{H-H}$ = 2.4 Hz, 2H; Ar-*H*), 6.79 (d, $^4J_{H-H}$ = 2.3 Hz, 2H; Ar-*H*), 5.97 (s, 1H; Ga*H*), 5.91 (s, 1H; Ga*H*), 4.63 (s, 1H; N*H*), 4.53 (s, 1H; N*H*), 1.40 (s, 18H; C(CH_3)$_3$), 1.32 (s, 18H; C(CH_3)$_3$), 1.25 (s, 18H; C(CH_3)$_3$), 1.22 (s, 18H; C(CH_3)$_3$).

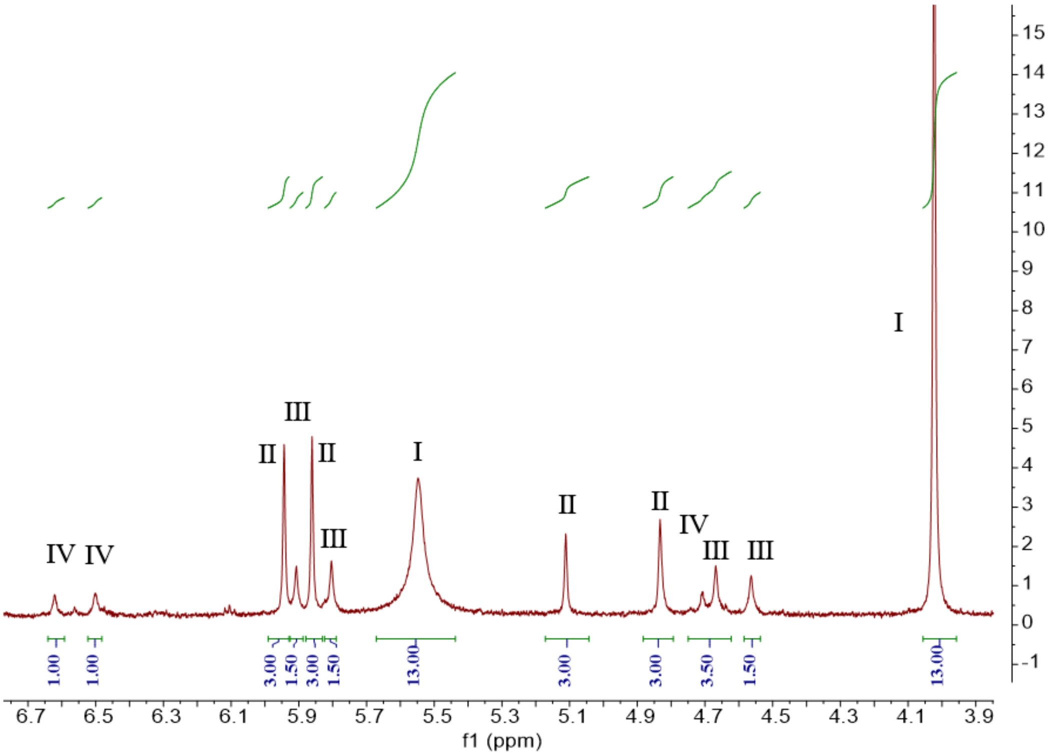

Figure S15. Ga–H region of the room temperature ^{1}H NMR spectrum of complex **5** in C_6D_6.

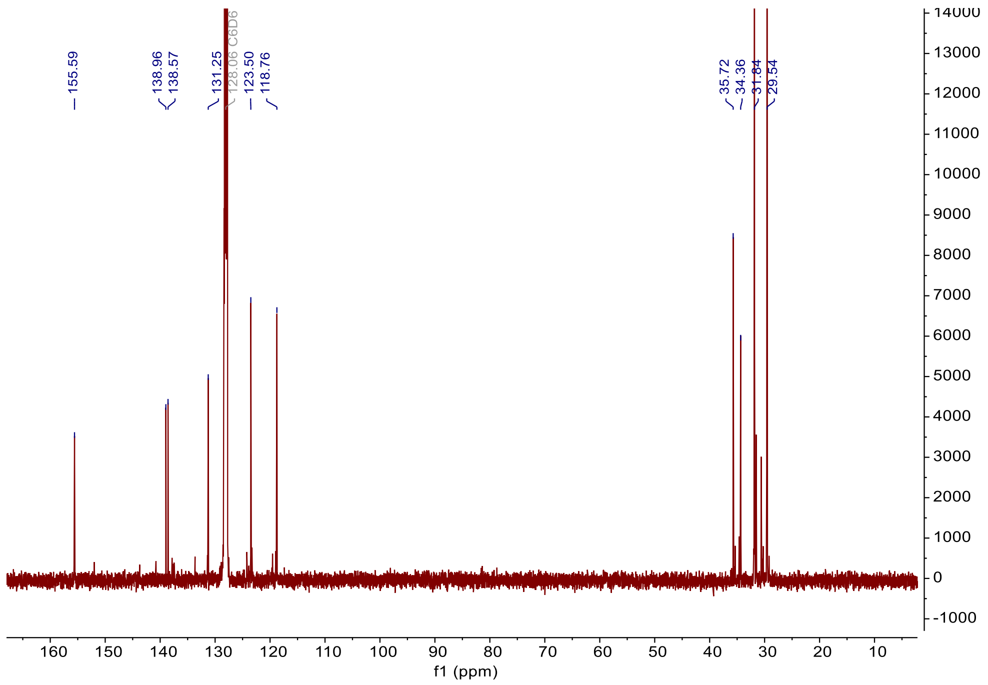

Figure S16. ^{13}C NMR spectrum of compound **5** in C_6D_6 at room temperature.

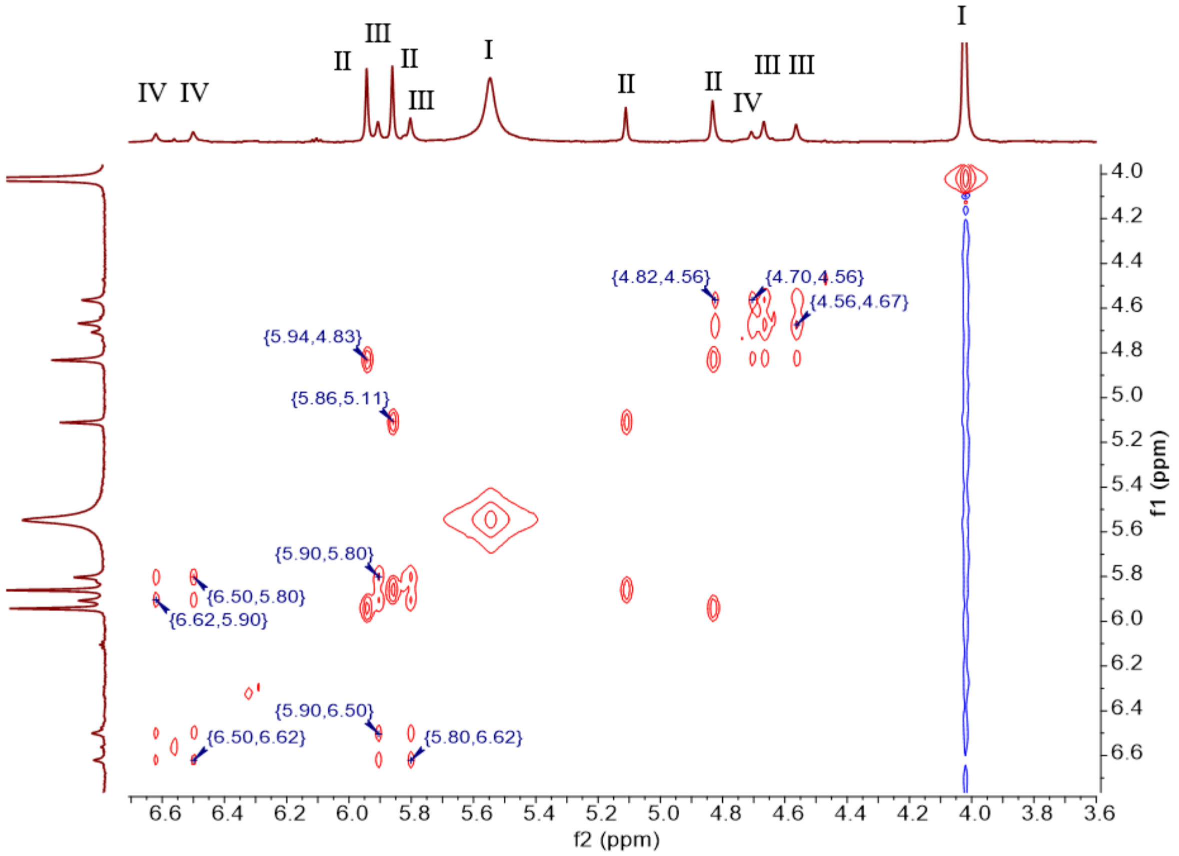

Figure S17. 2D NOESY NMR spectrum of **5** in C_6D_6 at room temperature.

NMR resonances at 4.01 (**I**), 4.56 (**III**), 4.67 (**III**), 4.70 (**IV**), 4.82 (**II**) and 5.11 (**II**) ppm are assigned as N*H* peaks of the ligand backbone. Resonances at 5.55 (**I**), 5.80 (**III**), 5.86 (**II**), 5.90 (**III**), 5.94 (**II**), 6.50 (**IV**), 6.62 (**IV**) ppm are assigned as Ga*H* peaks. The exchanging peaks shown above are listed in the table below. The NOESY NMR spectrum indicates that the N*H* resonance of **II** at 4.82 ppm is exchanging with the N*H* peak of **III** at 4.56 ppm, 4.67 ppm and **IV** at 4.70 ppm. The Ga*H* resonance of **III** at 5.80 ppm are exchanging with **III** at 5.90 ppm.

Table S1. Resonances and assignment of 2D NOESY spectrum of **5**.

Entry	f2 (ppm)	Isomer	f1 (ppm)	Isomer
1	6.62	IV	6.50	IV
2	6.62	IV	5.90	III
3	6.50	IV	5.80	III
4	5.94	II	4.83	II
5	5.90	III	5.80	III
6	5.90	III	6.50	IV
7	5.86	II	5.11	II
8	5.80	III	6.62	IV
9	4.82	II	4.67	III
10	4.82	II	4.56	II
11	4.70	IV	4.67	III
12	4.70	IV	4.56	II
13	4.56	III	4.67	III

One-dimension selective gradient NOESY:

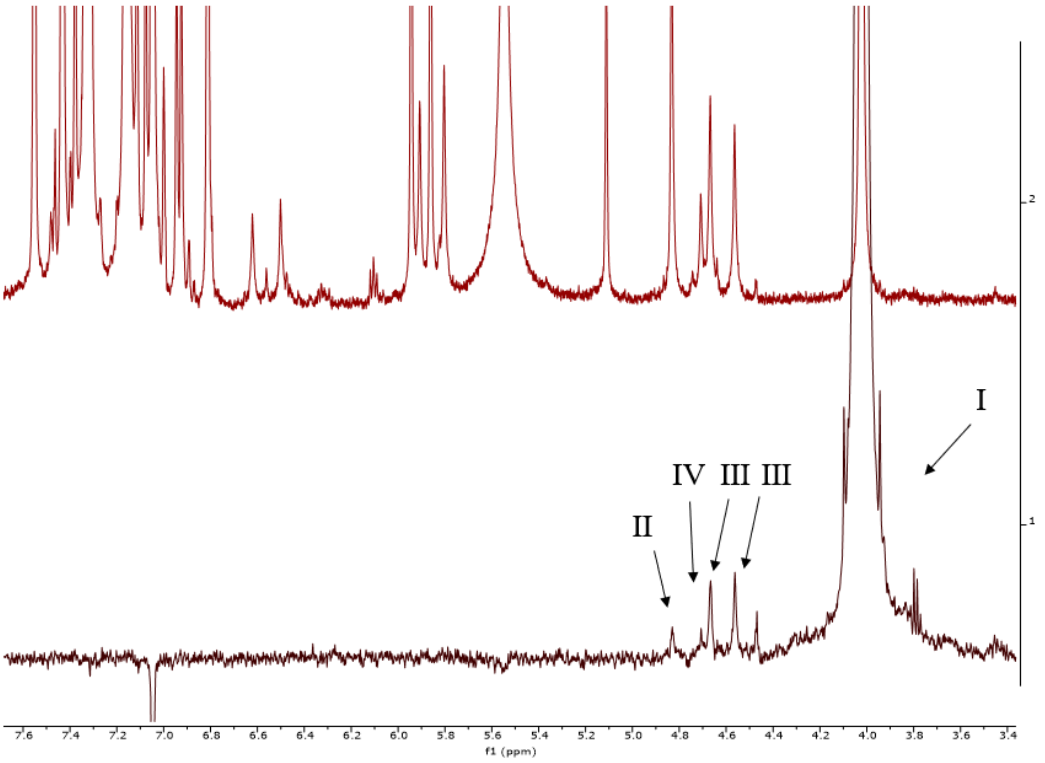

Figure S18. One-dimensional selective gradient NOESY spectrum of **5** at room temperature in C_6D_6.

Irradiation of N*H* (**I**) at the 4.0 ppm in C_6D_6 with the mixing time of 0.1s, showing N*H* at 4.0 ppm is exchanging with N*H* of **5**$_{\mathbf{II}}$, **5**$_{\mathbf{III}}$, **5**$_{\mathbf{IV}}$ at 4.82 (**II**), 4.56 and 4.67 (**III**), 4.70 ppm (**IV**) respectively. This N*H* of **5**$_{\mathbf{I}}$ at 4.0 ppm has NOE signal with Ar*H* of **5I** at 7.03 ppm and *tert*-butyl peak at 1.30 ppm and 1.58 ppm.

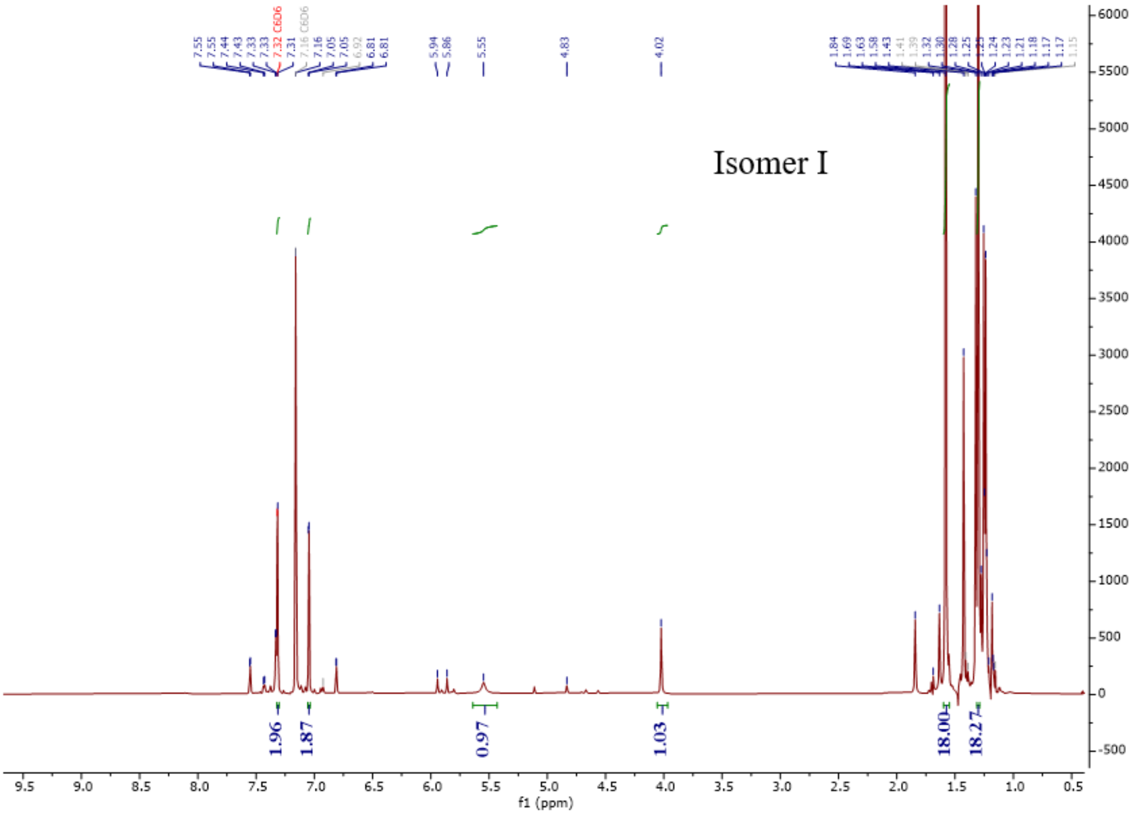

Figure S 19. ^{1}H NMR spectrum of **5** in C_6D_6 (with resonances for isomer **5**$_{\mathbf{I}}$ highlighted/integrated).

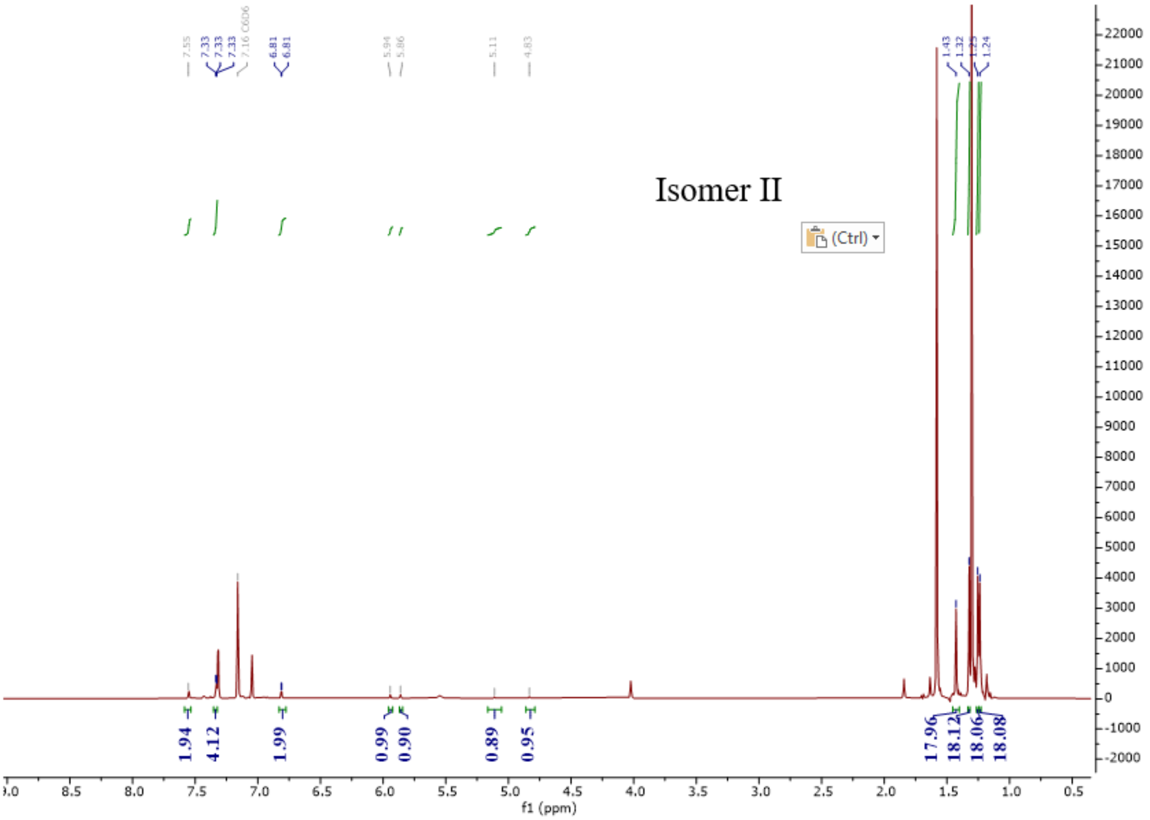

Figure S20. ^{1}H NMR spectrum of isomer **5** in C_6D_6 at room temperature (with resonances for isomer $\mathbf{5_{II}}$ highlighted/integrated).

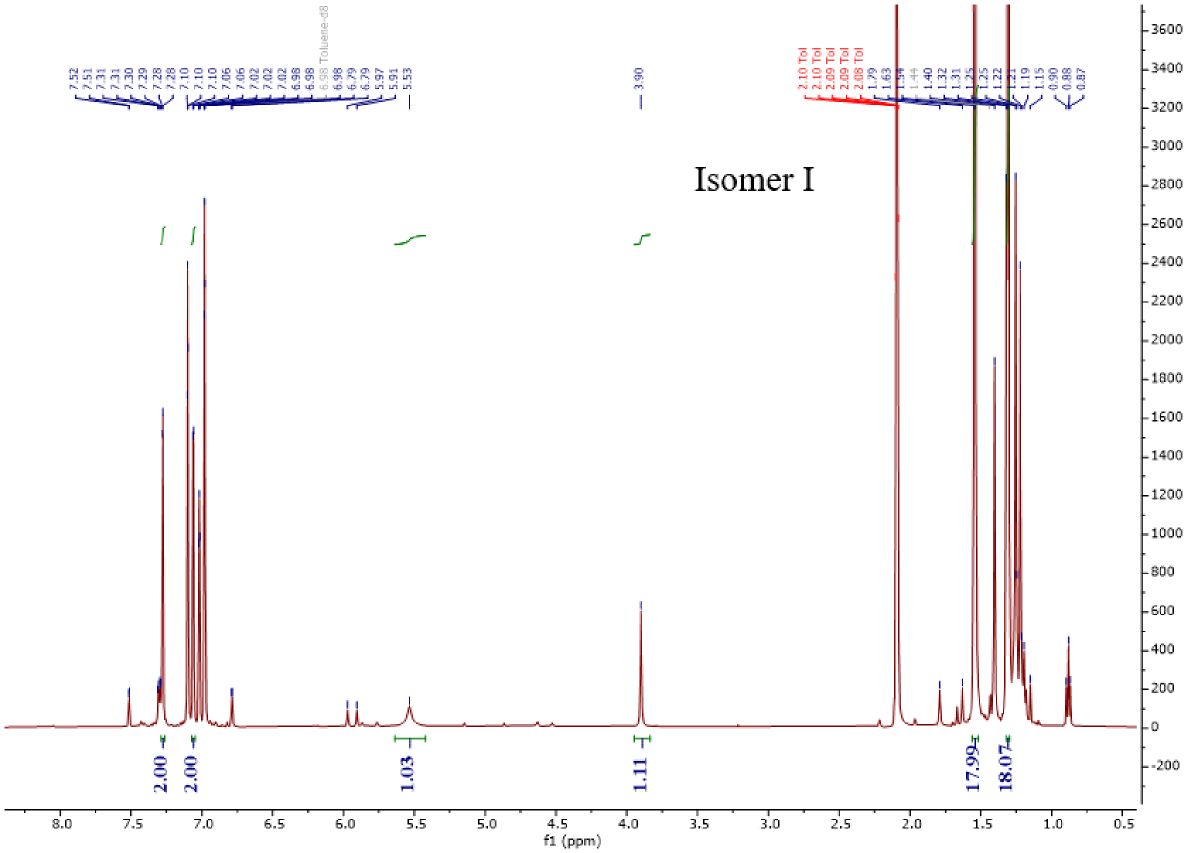

Figure S21. ^{1}H NMR spectrum of **5** in toluene-d_8 at room temperature (with resonances for isomer $\mathbf{5_I}$ highlighted/integrated).

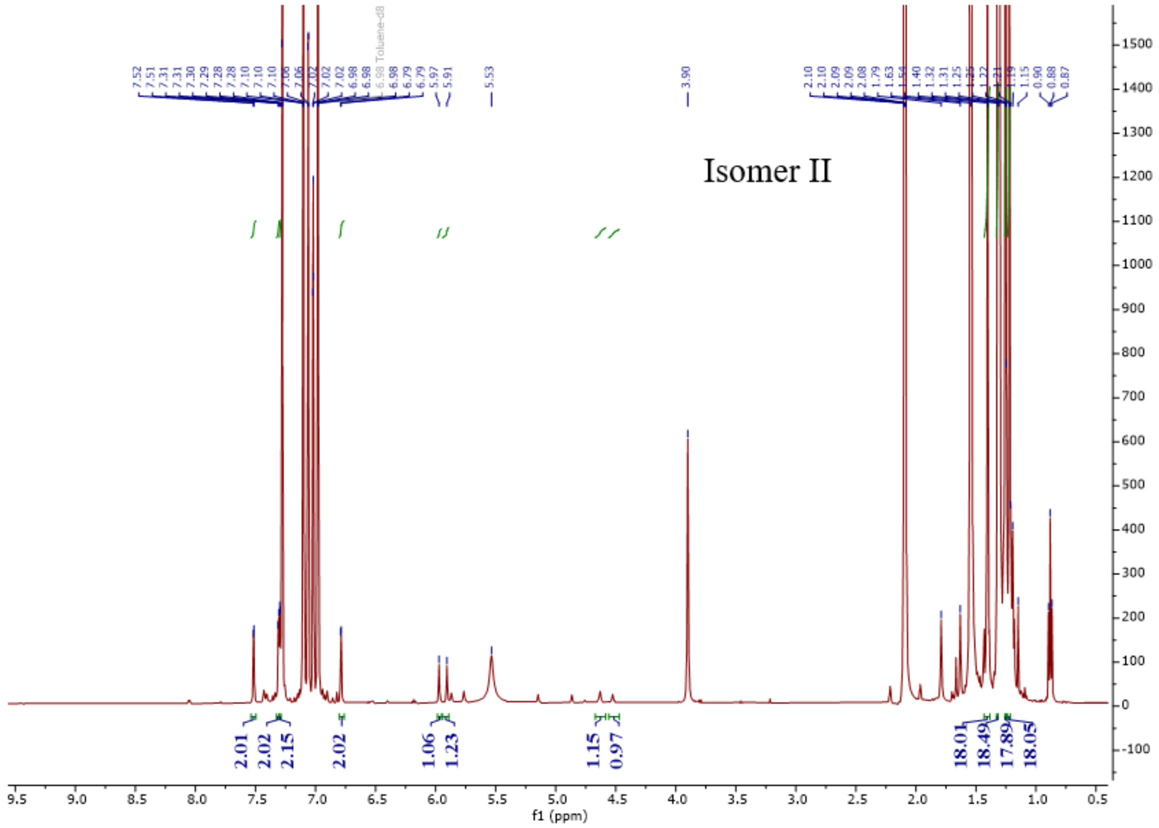

Figure S22. ^{1}H NMR spectrum of **5** in toluene-d_8 at room temperature (with resonances for isomer **5$_{\mathbf{II}}$** highlighted/integrated).

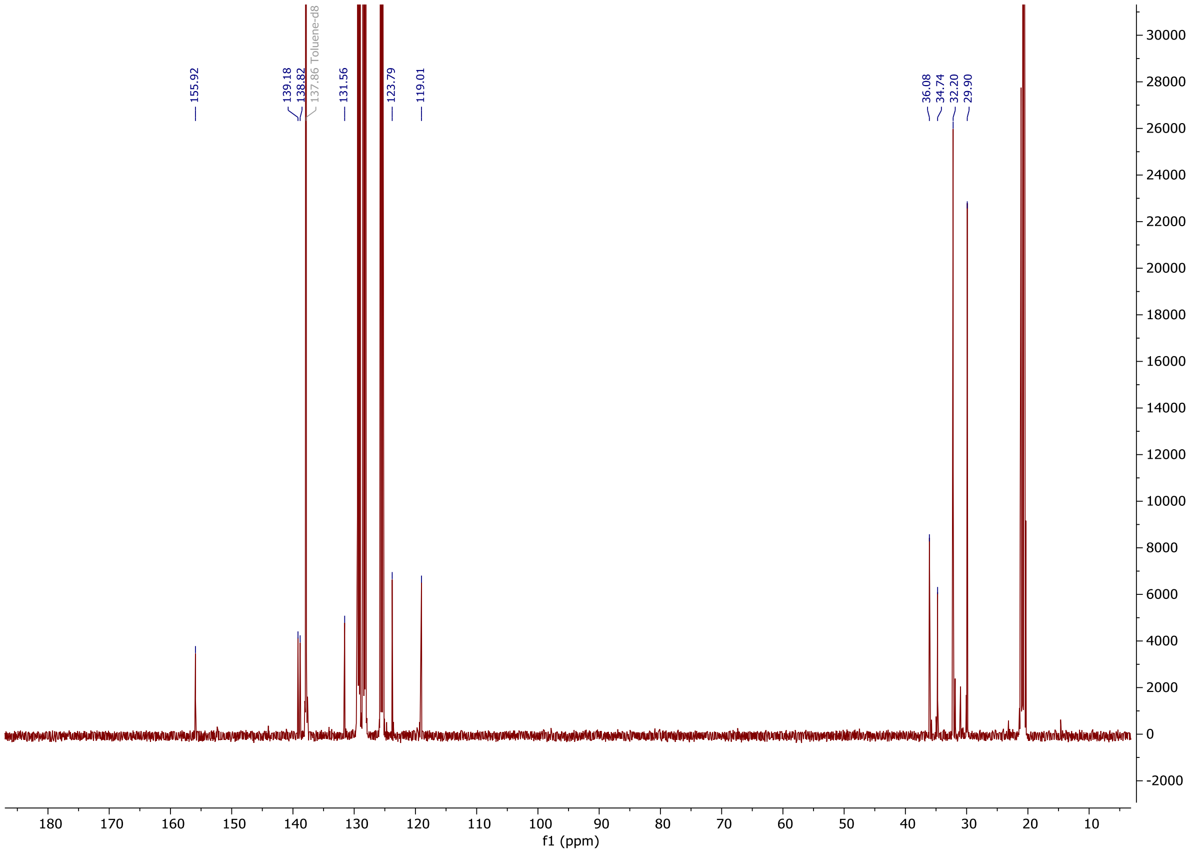

Figure S23. ^{13}C NMR spectrum of compound **5** in toluene-d_8 at room temperature.

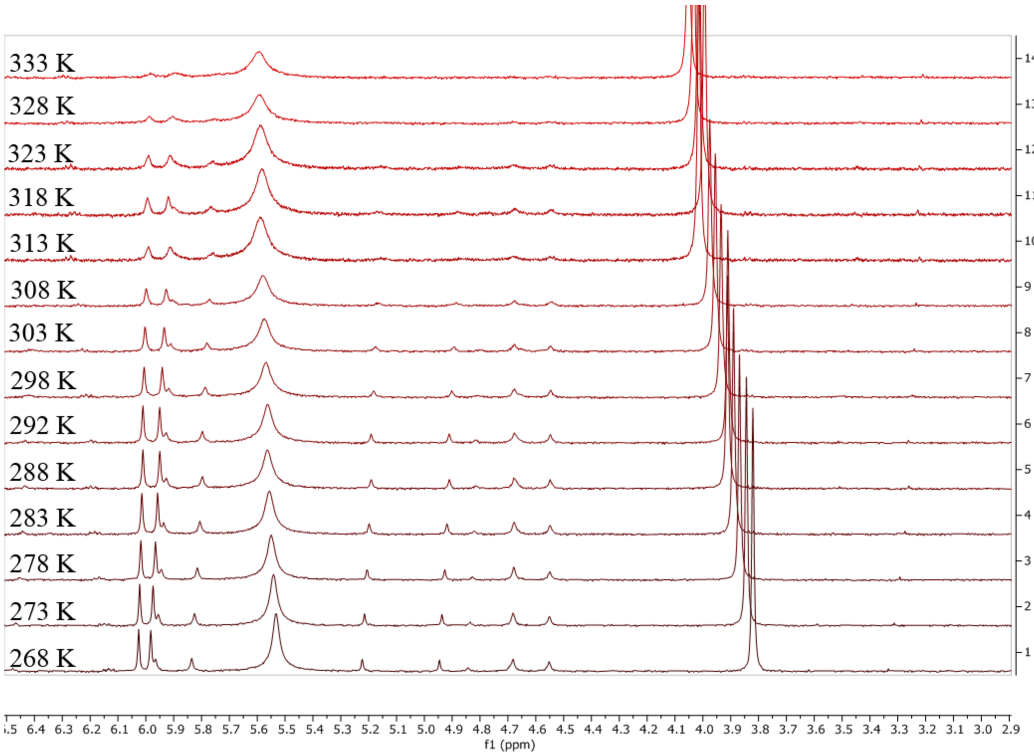

Figure S24. Variable-temperature ^{1}H NMR spectra of **5** (268–333K) in toluene-d_8.

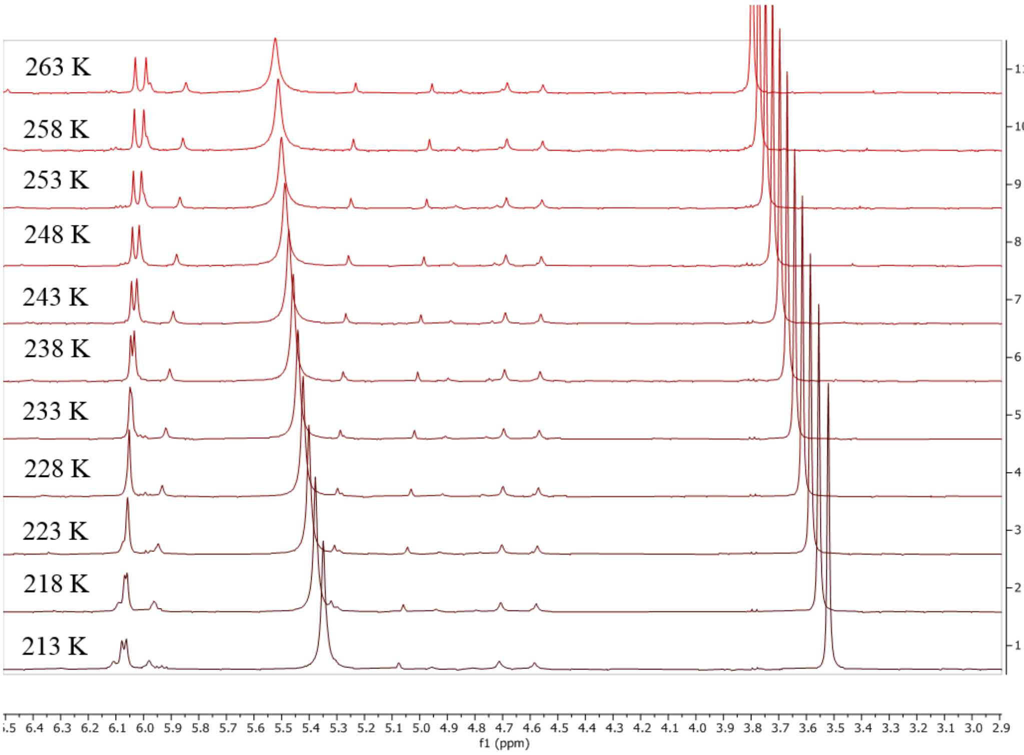

Figure S25. Variable-temperature ^{1}H NMR spectra of **5** (213–263K) in toluene-d_8.

The Variable-temperature NMR studies indicate that the relative concentration of compound **5$_{\mathbf{I}}$** gradually increases at temperatures above 298K.

Van't Hoff Plot of **5**:

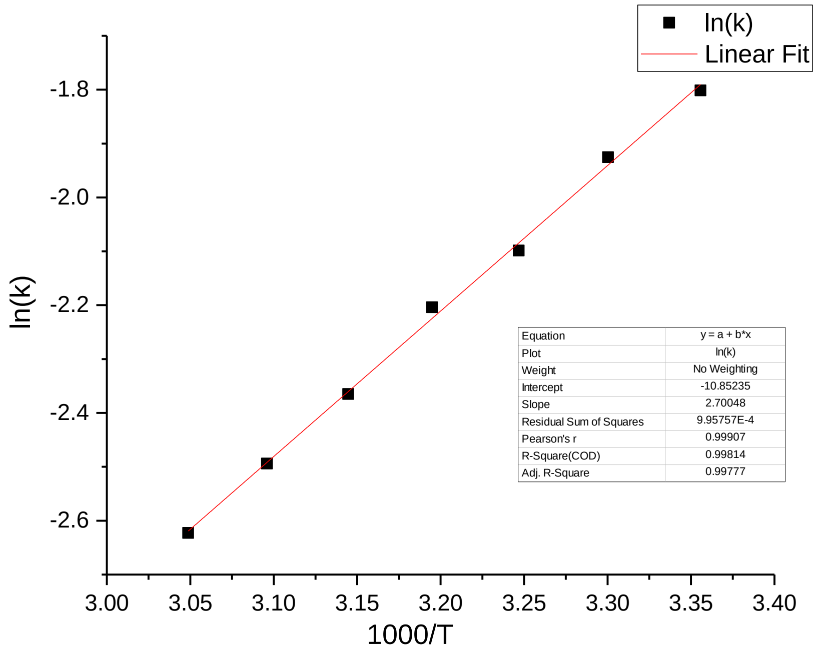

Figure S26. Van't Hoff plot of equilibrium between isomer **I** and **II**.

For the equilibrium between isomer **I** and **II**:

$$\Delta H = -R \times slope = -8.314 J \cdot K^{-1} \cdot mol^{-1} \times (2700) = -22.45\ KJ \cdot mol^{-1}$$

$$\Delta S = R \times intercept = 8.314 J \cdot K^{-1} \cdot mol^{-1} \times (-10.86) = -90.29\ J \cdot K^{-1} \cdot mol^{-1}$$

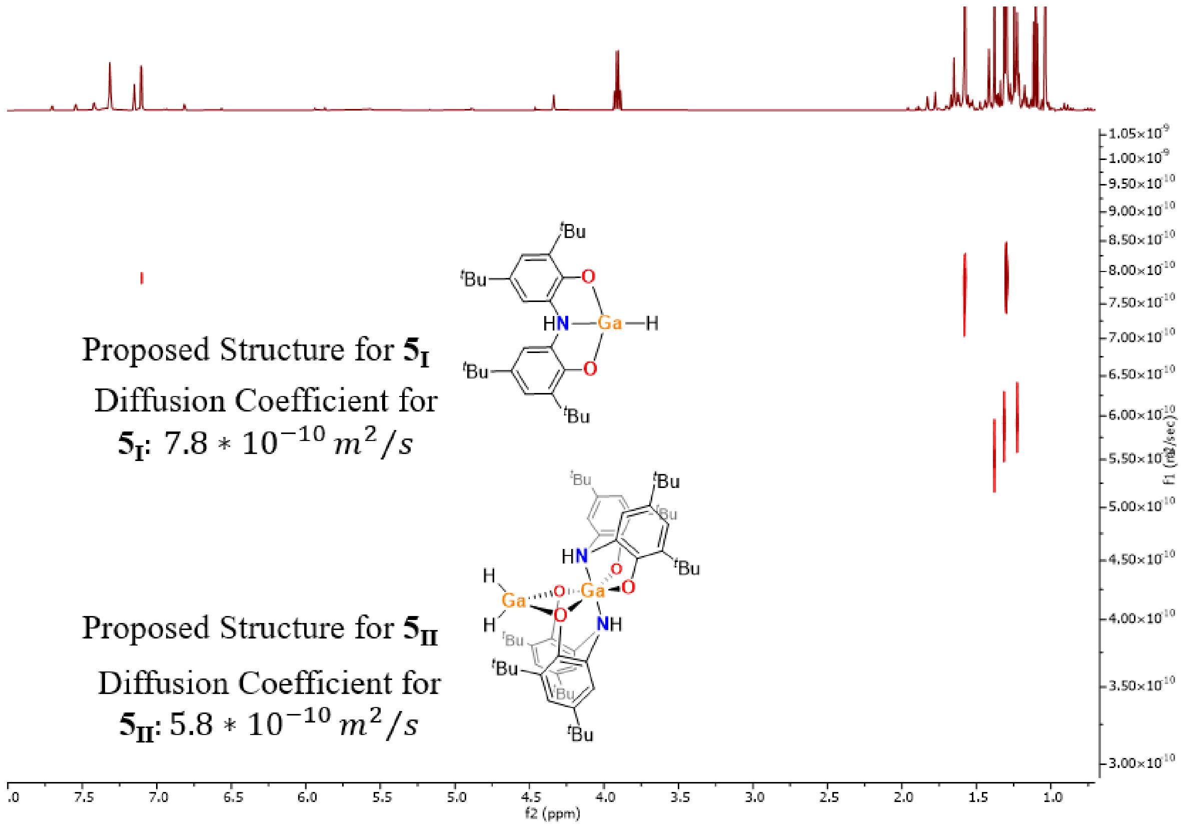

Figure S27. DOSY NMR spectrum of **5** in C_6D_6.

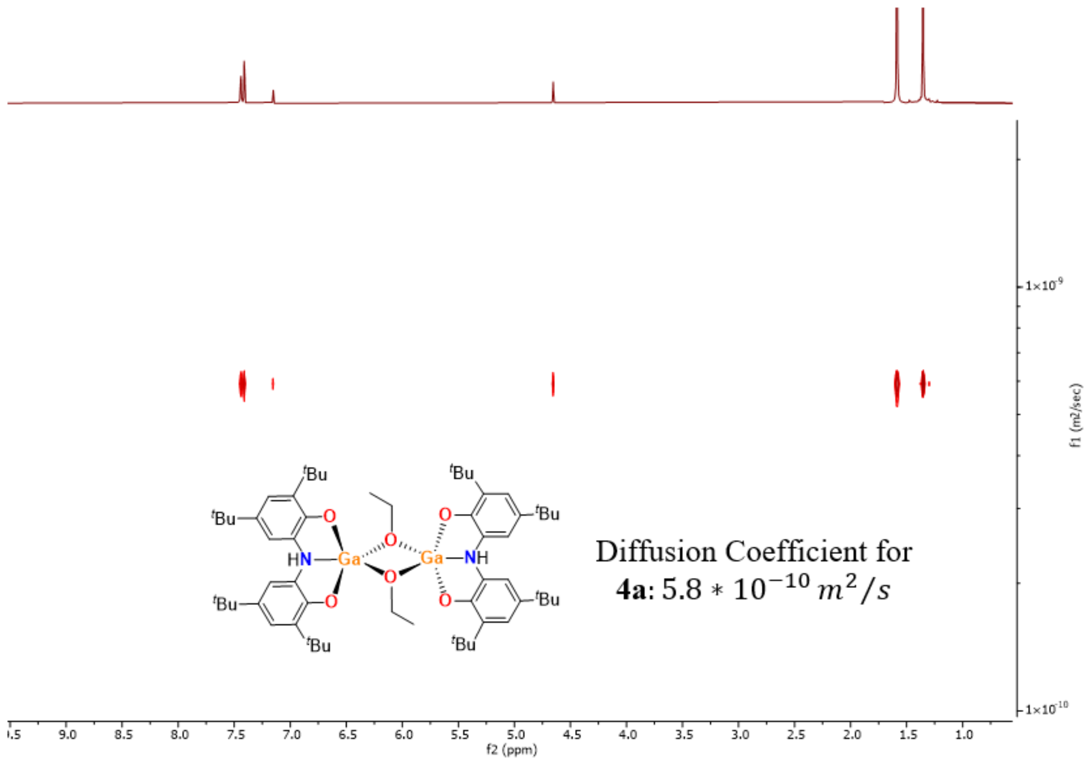

Figure S28. DOSY NMR spectrum of **4a** in C_6D_6.

The SEGWE (Stokes-Einstein Gierer-Wirtz Estimation) calculator was used for the calculation of molecular weights.[3,4]

Solution diffusion-ordered spectroscopy (DOSY) (Figure S27) of a C_6D_6 solution of **5** suggests allows for the observation of two isomers with different diffusion coefficients, $7.8\cdot10^{-10}\cdot m^2\cdot s^{-1}$ (isomer $\mathbf{5_I}$; with *tert*-butyl signals at 1.58, 1.30 ppm) and $5.8\cdot10^{-10}\cdot m^2\cdot s^{-1}$ (isomer $\mathbf{5_{II}}$; with *tert*-butyl signals at 1.32, 1.25, 1.24 ppm). The concentrations of other isomers are relatively low and affected by a poor signal to noise ratio, so they could not be detected.

Calculations suggests that, at 298.2K, the diffusion coefficient $7.8\cdot10^{-10}\cdot m^2\cdot s^{-1}$ corresponds to a species with a molecular weight of 445 $g\cdot mol^{-1}$ and a hydrodynamic radius of $6.6\cdot10^{-10}\cdot m$. While the diffusion coefficient $5.8\cdot10^{-10}\cdot m^2\cdot s^{-1}$ corresponds to a predicted molecular weight around 846 $g\cdot mol^{-1}$ and a hydrodynamics radius of $8.1\cdot10^{-10}\cdot m$. These results suggest that the molecular weight of $\mathbf{5_{II}}$ is roughly twice of $\mathbf{5_I}$.

For comparison, the DOSY NMR spectrum of **4a** (Figure S28) has a diffusion coefficient of $5.8\cdot10^{-10}\cdot m^2\cdot s^{-1}$ which is comparable to the diffusion coefficient for $\mathbf{5_{II}}$. Taking this into consideration, we postulate that species $\mathbf{5_{II}}$ is a dimer as suggested by single-crystal X-ray diffraction, whereas $\mathbf{5_I}$ is presumed to be a monomeric gallium hydride.

Table S2. [a] Predicted molecular weight and hydrodynamic radii were calculated by the diffusion coefficient using the SEGWE calculator, [b] Computed radii were calculated by density functional theory (DFT) with the Gaussian16 (Revision A.03) program package. [c] The experimental radius is estimated based on the single crystal X-ray diffraction structure.

Complex	Diffusion coefficient ($10^{-10}\cdot m^2\cdot s^{-1}$)	Predicted molecular weight ($g\cdot mol^{-1}$)[a]	Molecular Weight ($g\cdot mol^{-1}$)
**5$_{I}$**	7.8	445	494
**5$_{II}$**	5.8	846	988
**4a**	5.8	846	1077

Complex	Predicted radius (Å)[a]	Computed radius (Å)[b]	Experimental radius (Å)[c]
**5$_{I}$**	6.6	6.5	N/A
**5$_{II}$**	8.1	8.5	8.4
**4a**	8.1	9.9	9.8

2.8. Preparation of Ga[ON(H)O]H(THF) (**3**)

In a glove box, pinacolborane (17 µl, 15.0mg, 0.117mmol) was added to a suspension of **5** (60 mg, 0.056mmol) in hexane (2 mL) using a micro syringe, which afforded a colourless solution after stirring for 2 minutes. THF (10 µl, 8.9mg, 0.123mmol) was added and the solution stored at –30 °C overnight, resulting in precipitation of a white solid. The supernatant was decanted and the remaining solid was washed with *n*-pentane (3 × 2 mL) and subsequently dried *in vacuo* to afford **6** as a white powder (35mg, 0.062 mmol, 55% yield). Elemental analysis calculated for $C_{32}H_{50}GaNO_3$: C, 67.85%; H, 8.90%; N, 2.47%; found: C, 67.83%; H, 8.89%; N, 2.53%. ^{1}H NMR (400 MHz, C_6D_6): δ (ppm) 7.35 (d, $^4J_{H-H}$ = 2.4 Hz, 2H; Ar-*H*), 7.15 (d, $^4J_{H-H}$ = 2.4 Hz, 2H; Ar-*H*), 5.65 (s, 1H; Ga*H*), 3.92 (s, 1H; N*H*), 3.90–3.81 (m, 4H; OCH_2), 1.59 (s, 18H; $C(CH_3)_3$), 1.48–1.41 (m, 4H; OCH_2CH_2), 1.35 (s, 18H; $C(CH_3)_3$). ^{13}C{^{1}H} NMR (101 MHz, C_6D_6): δ (ppm) 155.92 (Ar-*C*), 138.19 (Ar-*C*), 137.78 (Ar-*C*), 131.76 (Ar-*C*), 122.91 (Ar-*C*), 119.82 (Ar-*C*), 68.55 (OCH_2), 35.67 ($C(CH_3)_3$), 34.35 ($C(CH_3)_3$), 31.94 ($C(CH_3)_3$), 29.69 ($C(CH_3)_3$), 25.56 (OCH_2CH_2).

^{1}H NMR (500 MHz, $CDCl_3$): δ (ppm) 7.42 (d, $^4J_{H-H}$ = 2.4 Hz, 2H; Ar-*H*), 7.18 (d, $^4J_{H-H}$ = 2.4 Hz, 2H; Ar-*H*), 5.59 (s, 1H; Ga*H*), 5.26 (s, 1H; N*H*), 4.04–3.84 (m, 4H; OCH_2), 2.13–1.77 (m, 4H; OCH_2CH_2), 1.40 (s, 18H; $C(CH_3)_3$), 1.30 (s, 18H; $C(CH_3)_3$). ^{13}C{^{1}H} NMR (126 MHz, $CDCl_3$): δ (ppm) 155.54 (Ar-*C*), 138.69 (Ar-*C*), 138.18 (Ar-*C*), 130.72 (Ar-*C*), 123.53 (Ar-*C*), 119.07 (Ar-*C*), 68.60 (OCH_2), 35.54 ($C(CH_3)_3$), 34.36 ($C(CH_3)_3$), 31.79 ($C(CH_3)_3$), 29.43 ($C(CH_3)_3$), 25.71 (OCH_2CH_2).

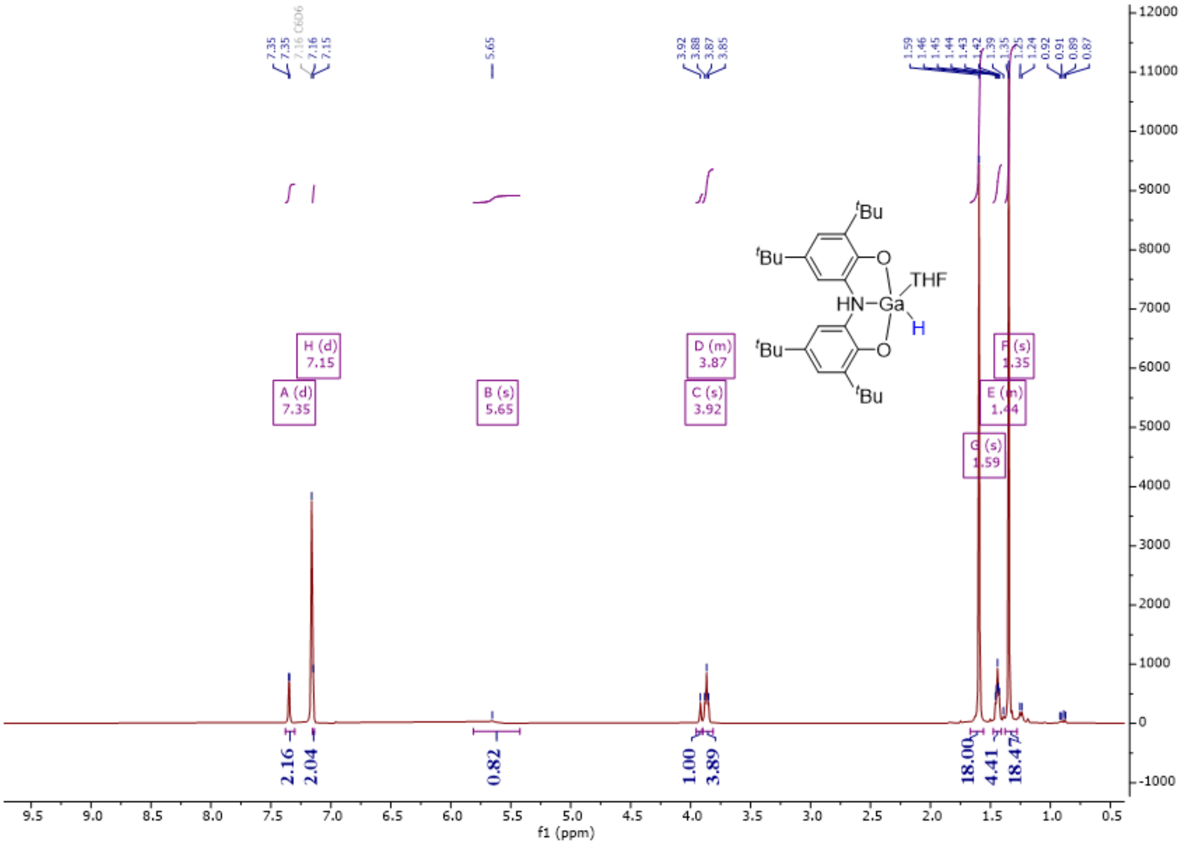

Figure S29. ^{1}H NMR spectrum for compound **3** in C_6D_6.

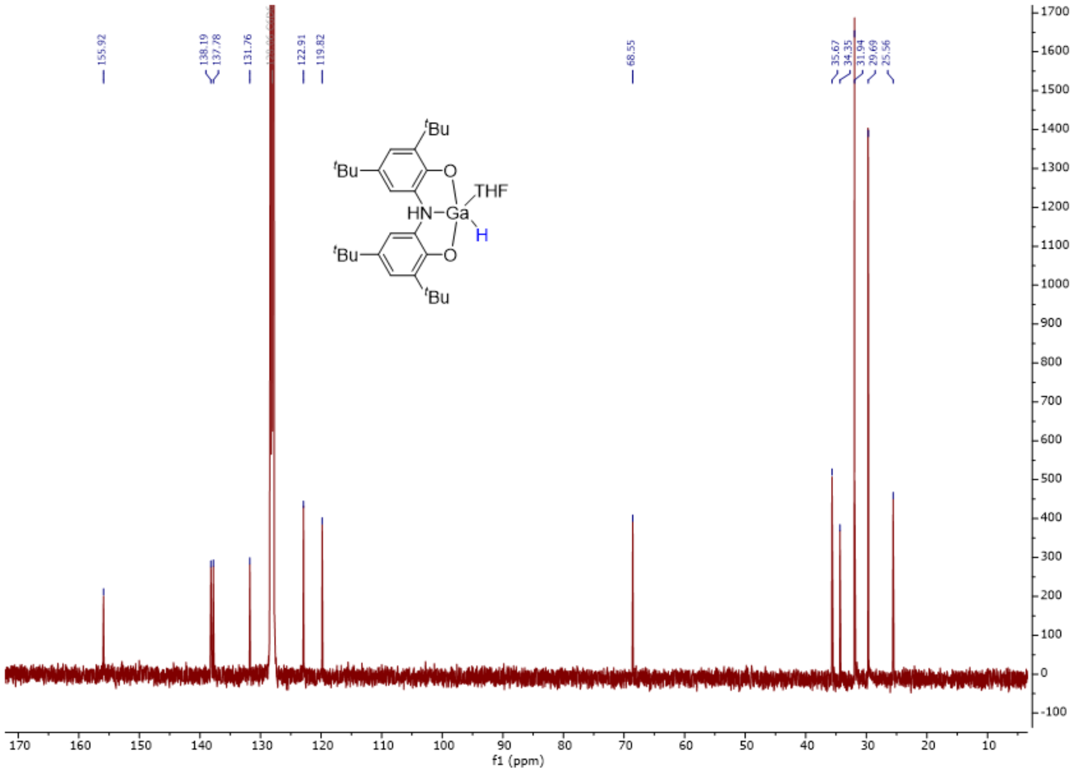

Figure S30. ^{13}C NMR spectrum for compound **3** in C_6D_6.

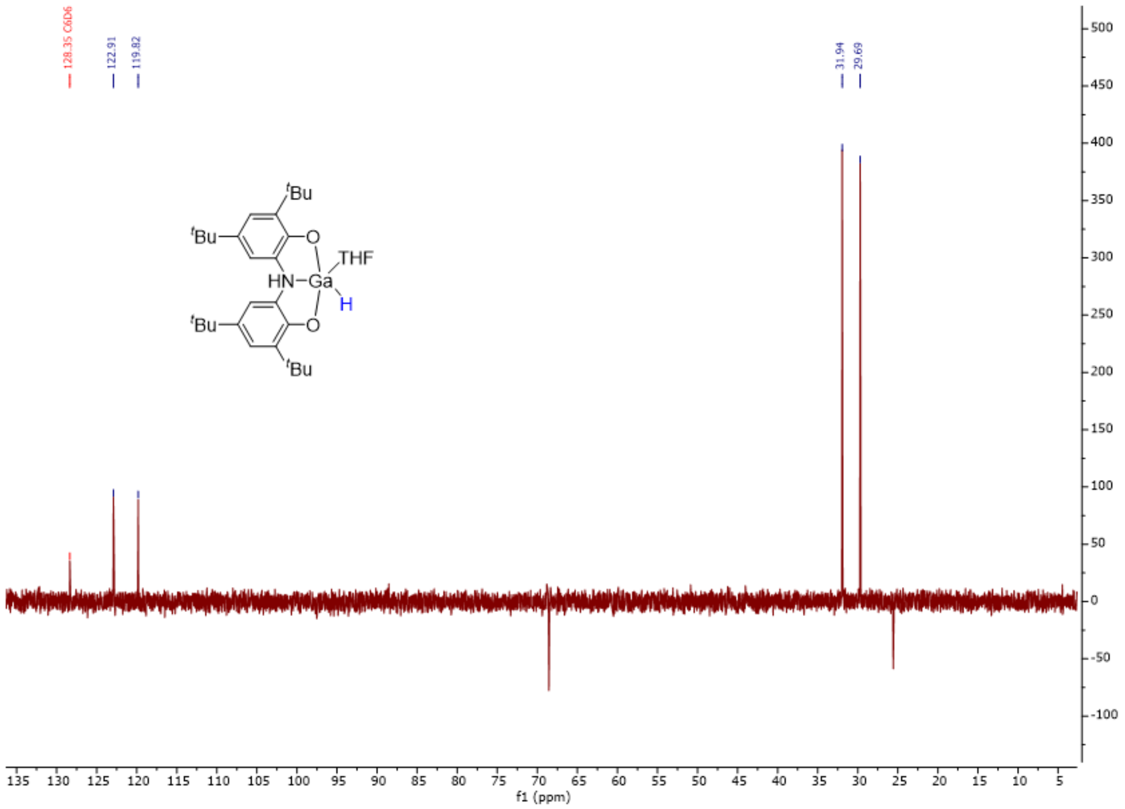

Figure S31. DEPT 135 NMR spectrum for compound **3** in C_6D_6.

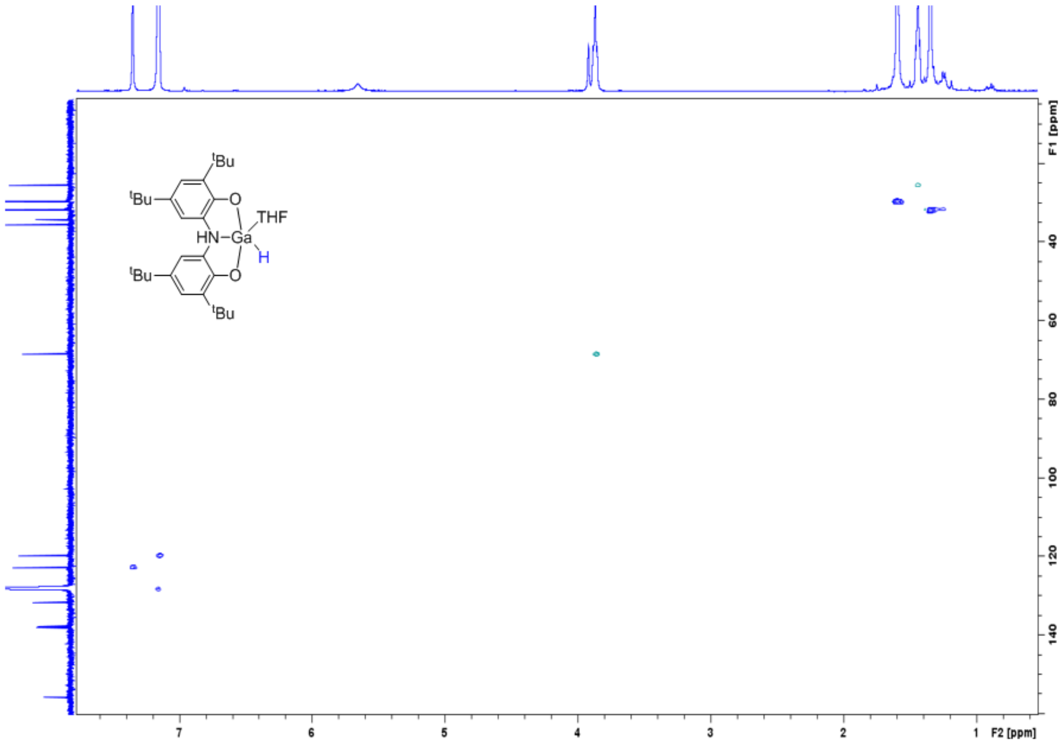

Figure S32. HSQC NMR spectrum for compound **3** in C_6D_6.

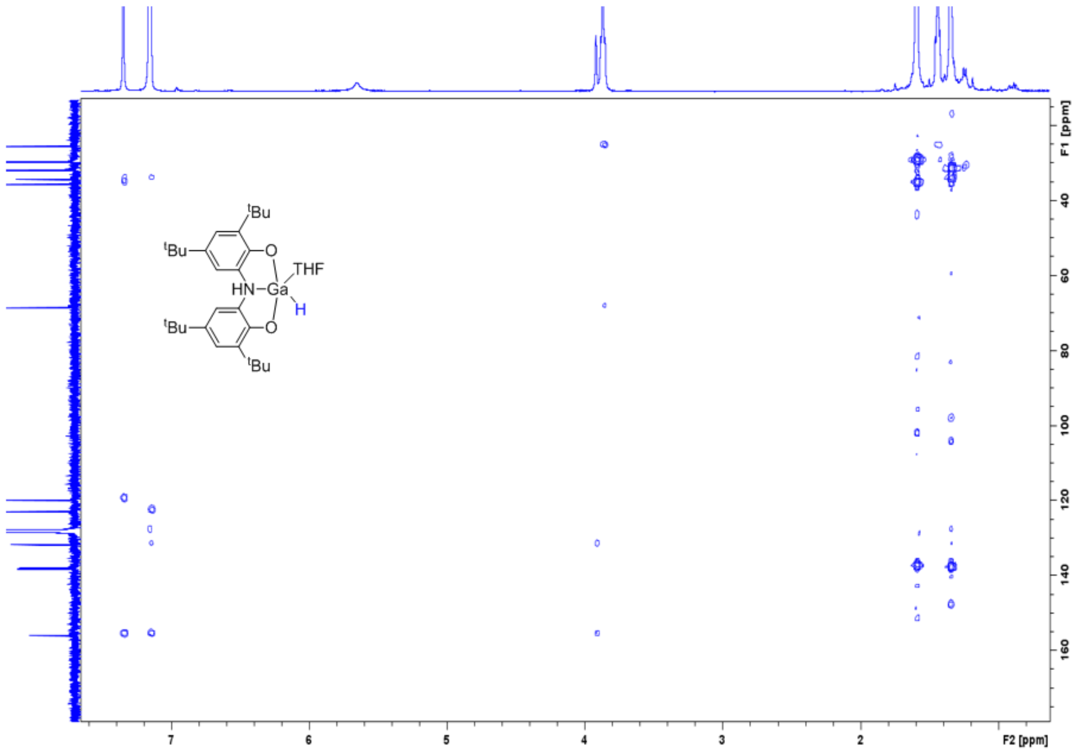

Figure S33. HMBC NMR spectrum for compound **3** in C_6D_6.

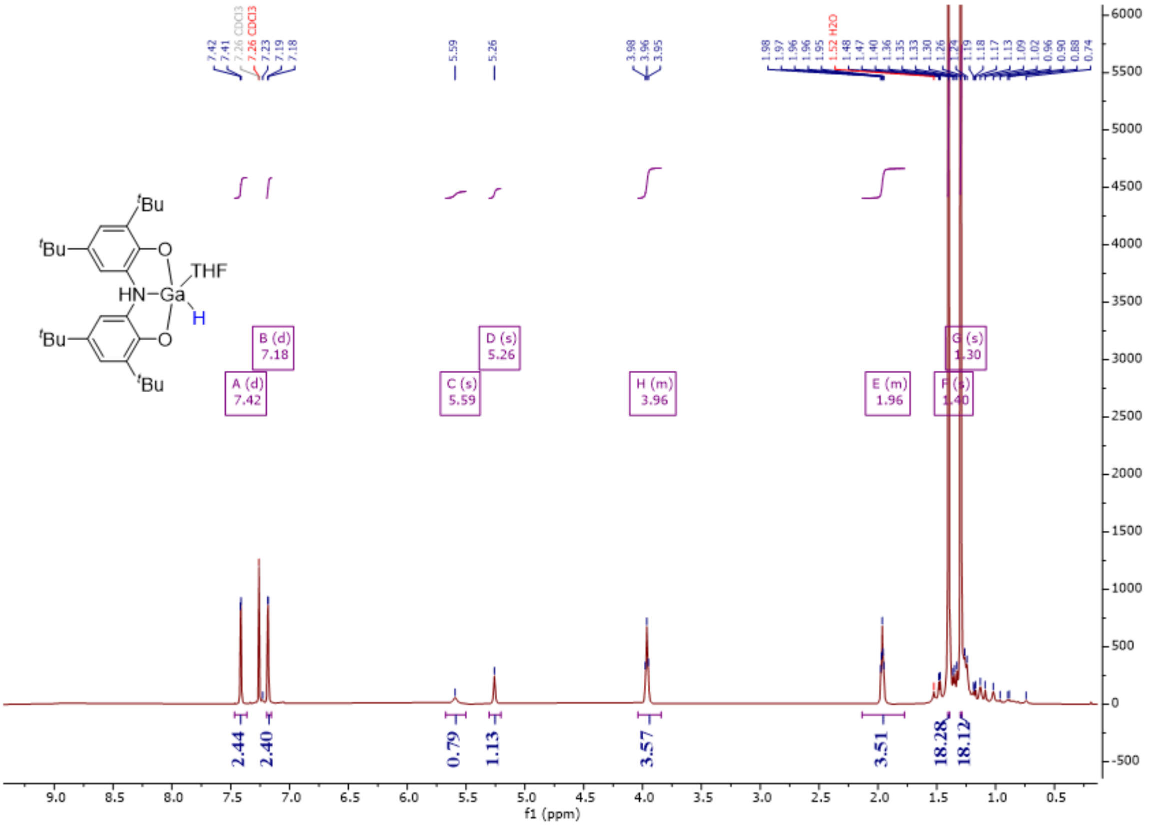

Figure S34. ^{1}H NMR spectrum for compound **3** in $CDCl_3$.

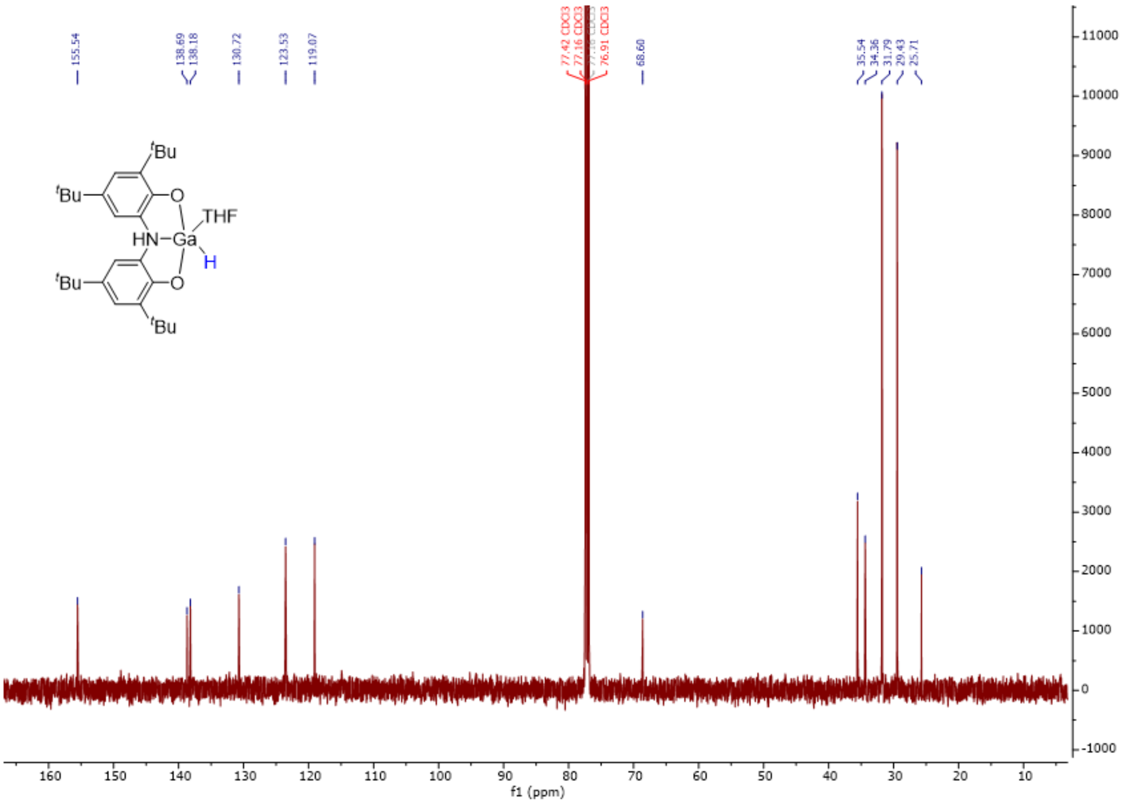

Figure S35. ^{13}C NMR spectrum for compound **3** in $CDCl_3$.

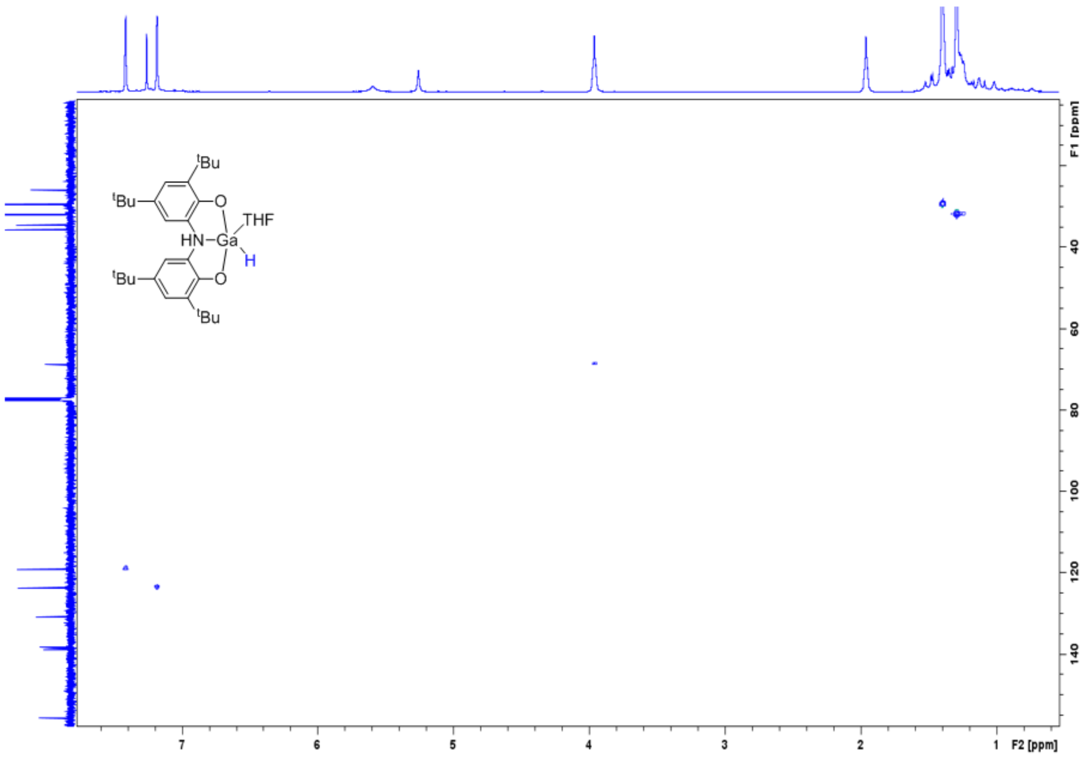

Figure S36. HSQC NMR spectrum for compound **3** in $CDCl_3$.

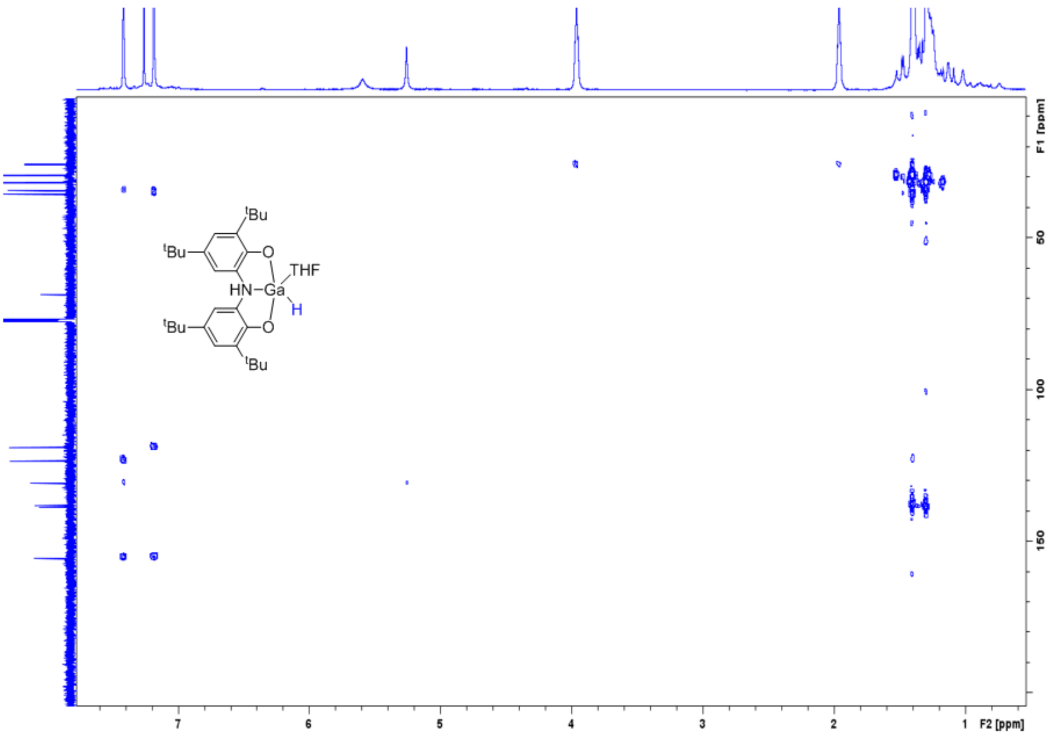

Figure S37. HMBC NMR spectrum for compound **3** in $CDCl_3$.

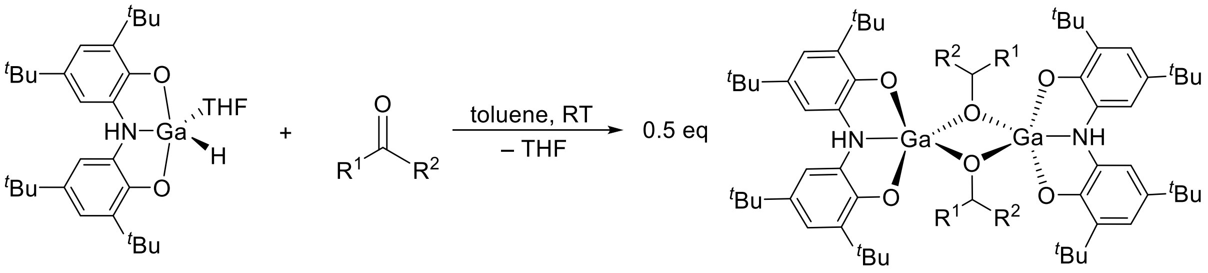

3

6, **7**
[Ga(ON(H)O)(OC(H)(R^1)R^2)]$_2$

Aldehydes
6a: R^1 = C_6H_5, R^2 = H
6b: R^1 = ^{i}Pr, R^2 = H
6c: R^1 = 4-OMeC_6H_4, R^2 = H
6d: R^1 = 4-BrC_6H_4, R^2 = H
6e: R^1 = Mes, R^2 = H

Ketones
7a: R^1 = C_6H_5, R^2 = C_6H_5
7b: R^1 = Me, R^2 = Me
7c: R^1 = 4-OMeC_6H_4, R^2 = Me
7d: R^1 = 4-BrC_6H_4, R^2 = Me
7e: R = 2-indanyl
7f: R^1 = 4-$NO_2C_6H_4$, R^2 = Me

Scheme S4. Synthetic route to compounds **6a**–**6e**, **7a**–**7f**.

2.9. General synthesis and characterisation data for {Ga[ON(H)O](OC(H)R^1R^2)}$_2$ (**6**, **7**).

3 (100 mg, 0.18 mmol) was dissolved in toluene (2 mL) giving a colourless solution. One molar equivalent of aldehyde or ketone (0.18 mmol) was added and the solution kept stirring overnight for all samples (note: benzophenone requires stirring for 5 days). The solvent was pumped and the solid remnants were washed with pentane (2 mL), then dried *in vacuo*, affording the desired products as white solids. X-ray quality crystals were obtained by from concentrated toluene, $CDCl_3$ or C_6D_6 solutions, yielding colourless crystals.

2.9.1. Preparation of {Ga[ON(H)O](OCH_2Ph)}$_2$ (**6a**)

In accordance with the general procedure using benzaldehyde (19 mg, 0.18 mmol) as the substrate to afford **6a**. (85 mg, 0.07 mmol, 82% yield). Elemental analysis calculated for $C_{70}H_{96}Ga_2N_2O_6$: C, 70.01%; H, 8.06%; N, 2.33%; found: C, 70.00%; H, 7.96%; N, 2.25%. ^{1}H NMR (400 MHz, THF-d_8): δ (ppm) 7.43 (d, $^3J_{H\text{–}H}$ = 7.1 Hz, 4H; Ar-*H*), 7.17 (d, $^4J_{H\text{–}H}$ = 2.4 Hz, 4H; Ar-*H*), 7.09 (d, $^4J_{H\text{–}H}$ = 2.4 Hz, 4H, Ar-*H*), 7.00 (m, 6H, Ar-*H*), 5.67 (s, 2H; N*H*), 5.29 (s, 4H; C*H*$_2$), 1.37 (s, 36H; C(C*H*$_3$)$_3$), 1.26 (s, 36H; C(C*H*$_3$)$_3$). ^{13}C{^{1}H} NMR (101 MHz, THF-d_8):

δ (ppm) 155.04 (Ar-*C*), 141.54 (Ar-*C*), 138.75 (Ar-*C*), 137.76 (Ar-*C*), 131.65 (Ar-*C*), 129.00 (Ar-*C*), 128.19 (Ar-*C*), 127.41 (Ar-*C*), 123.09 (Ar-*C*), 120.35 (Ar-*C*), 68.14 (O*C*H_2), 36.11 (*C*(CH_3)$_3$), 34.95 (*C*(CH_3)$_3$), 32.24 (C(*C*H_3)$_3$)., 30.05 (C(*C*H_3)$_3$).

^{1}H NMR (500 MHz, $CDCl_3$): δ (ppm) 7.53 (d, $^3J_{H–H}$ = 6.9 Hz, 4H; Ar-*H*), 7.19–7.04 (m, 10H; Ar-*H*), 7.01 (d, $^4J_{H–H}$ = 2.3 Hz, 4H; Ar-*H*), 5.22 (s, 4H; CH_2), 3.93 (s, 2H; N*H*), 1.39 (s, 36H; C(CH_3)$_3$), 1.27 (s, 36H; C(CH_3)$_3$). ^{13}C{^{1}H} NMR (126 MHz, $CDCl_3$): δ (ppm) 153.96 (Ar-*C*), 140.53 (Ar-*C*), 138.36 (Ar-*C*), 137.65 (Ar-*C*), 129.54 (Ar-*C*), 128.86 (Ar-*C*), 128.28 (Ar-*C*), 126.78 (Ar-*C*), 123.03 (Ar-*C*), 119.25 (Ar-*C*), 67.63 (O*C*H_2), 35.52 (*C*(CH_3)$_3$), 34.30 (*C*(CH_3)$_3$), 31.80 (C(*C*H_3)$_3$), 29.43 (C(*C*H_3)$_3$).

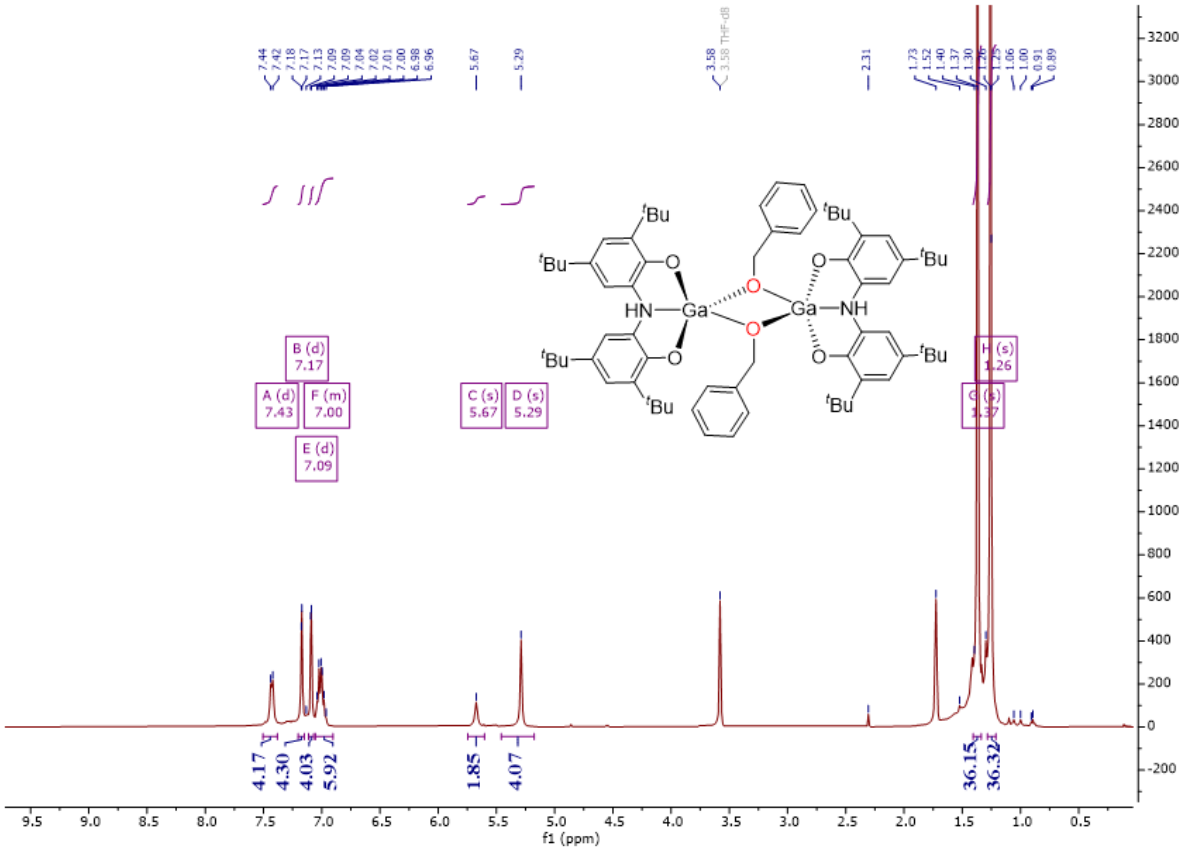

Figure S38. ^{1}H NMR spectrum for compound **6a** in THF-d_8.

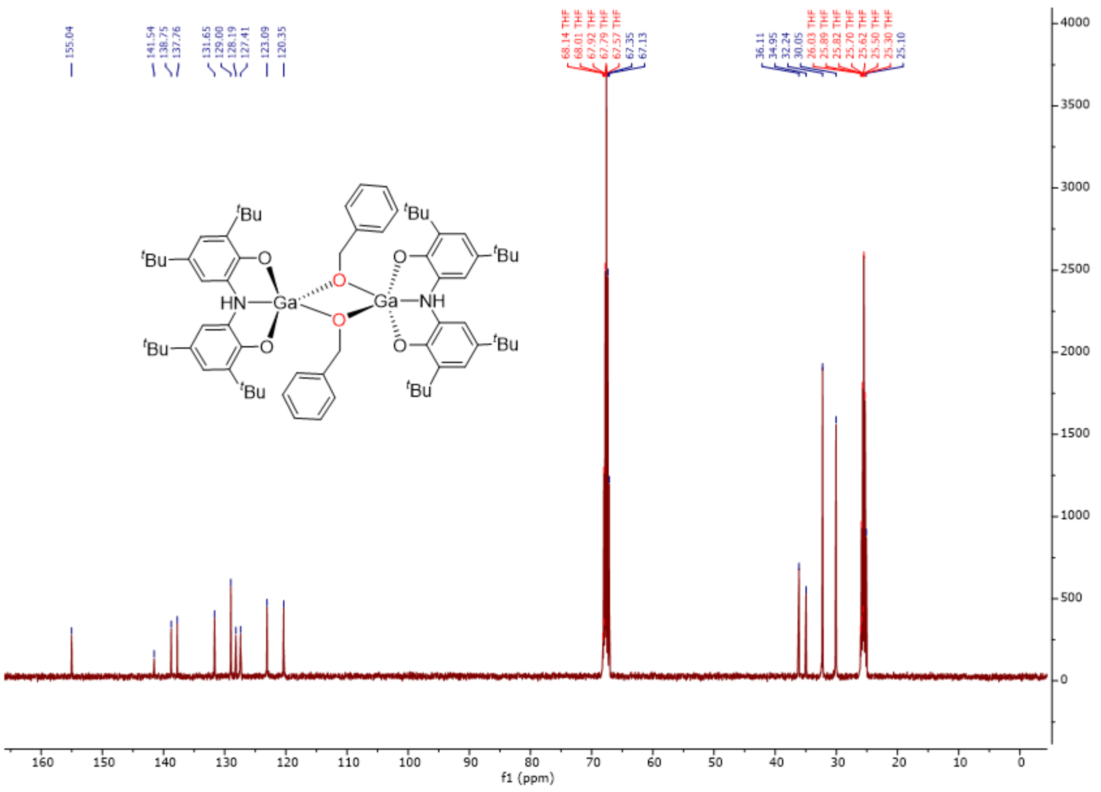

Figure S39. ^{13}C NMR spectrum for compound **6a** in THF-d_8.

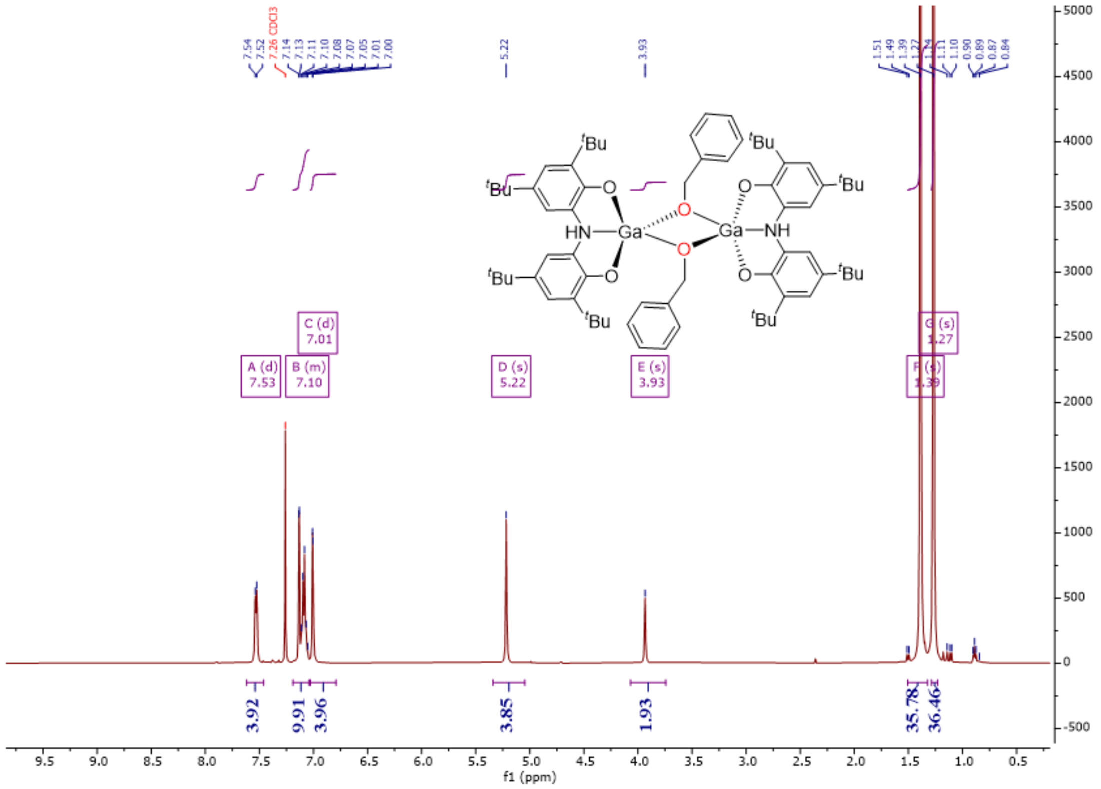

Figure S40. ^{1}H NMR spectrum for compound **6a** in $CDCl_3$

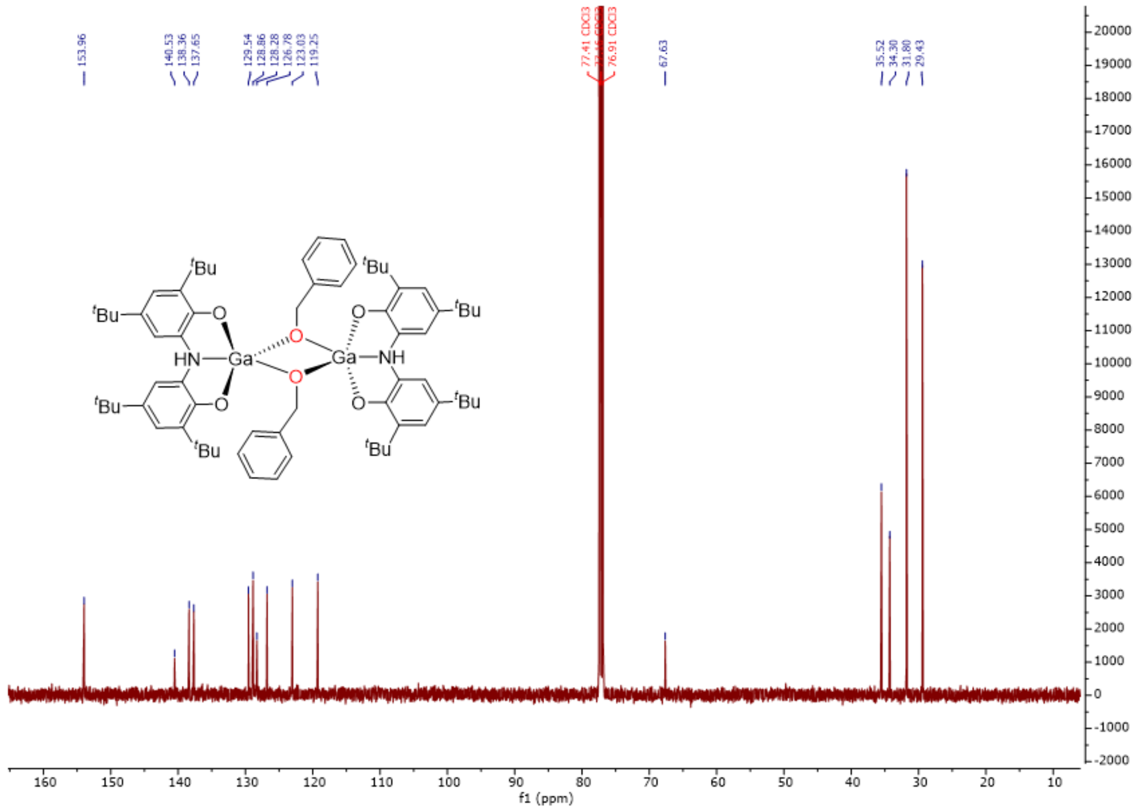

Figure S41. ^{13}C NMR spectrum for compound **6a** in $CDCl_3$.

2.9.2. Preparation of {Ga[ON(H)O](OCH$_2$CHMe$_2$)}$_2$ (**6b**)

In accordance with the general procedure described above using **3** (100 mg, 0.18 mmol) and isobutyraldehyde (17 µL, 0.19 mmol) to afford product **6b** (57 mg, 0.050 mmol, 57% yield). Elemental analysis calculated for $C_{64}H_{100}Ga_2N_2O_6$: C, 67.85%; H, 8.90%; N, 2.47%; found: C, 67.68%; H, 8.85%; N, 2.57%. ^1H NMR (400 MHz, $CDCl_3$): δ (ppm) 7.53 (br, 4H; Ar-*H*), 7.21 (br, 4H; Ar-*H*), 5.01 (br, 2H; N*H*), 3.79 (br, 4H; OC*H*$_2$), 2.09 (br, 2H; C*H*Me$_2$), 1.38 (s, 36H; C(C*H*$_3$)$_3$), 1.33 (s, 36H; C(C*H*$_3$)$_3$), 1.03 – 0.66 (m, 12H; CH(C*H*$_3$)$_2$).$^{13}C\{^1H\}$ NMR (101 MHz, $CDCl_3$): δ (ppm) 154.25 (Ar-*C*), 138.66 (Ar-*C*), 137.98 (Ar-*C*), 129.56 (Ar-*C*), 123.39 (Ar-*C*), 119.39 (Ar-*C*), 72.81 (O*C*H$_2$), 35.47 (*C*(CH$_3$)$_3$), 34.43 (*C*(CH$_3$)$_3$), 31.83 (C(*C*H$_3$)$_3$), 30.74 (O*C*H(CH$_3$)$_2$), 29.41 (C(*C*H$_3$)$_3$), 19.32 (OCH(*C*H$_3$)$_2$).

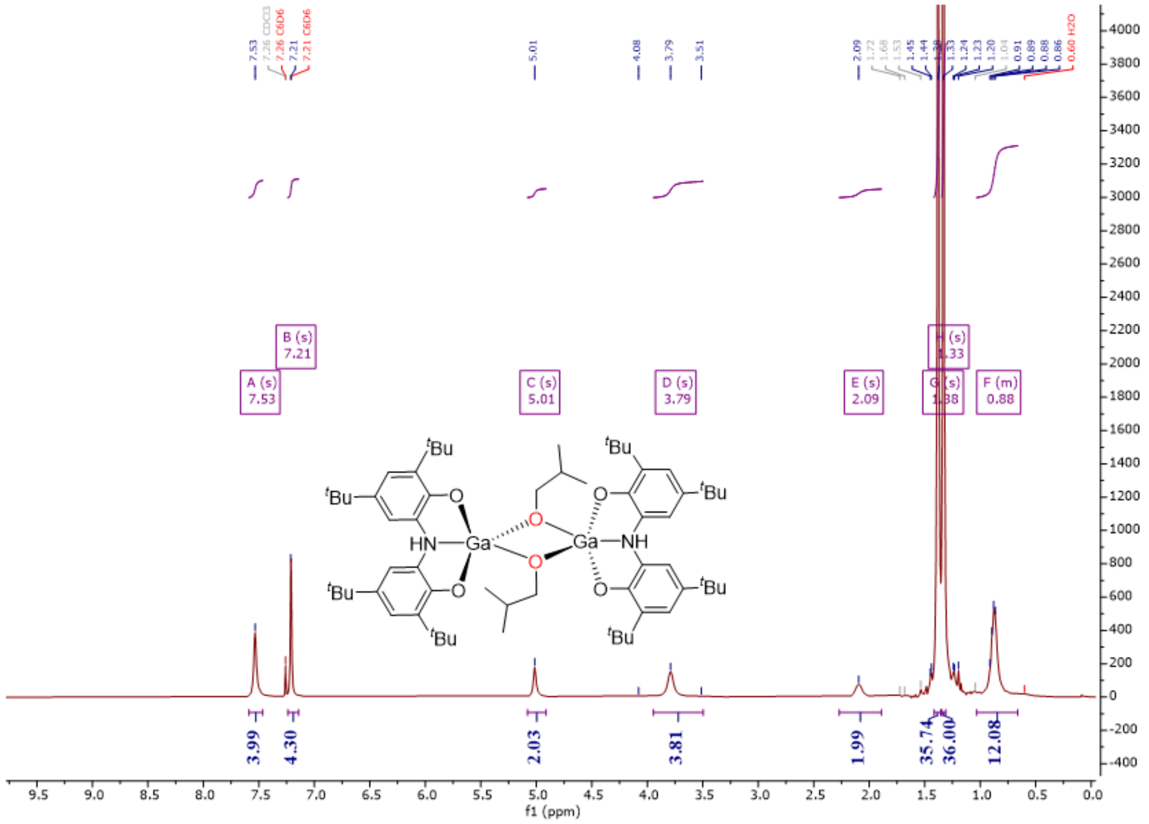

Figure S42. ^{1}H NMR spectrum for compound **6b** in $CDCl_3$.

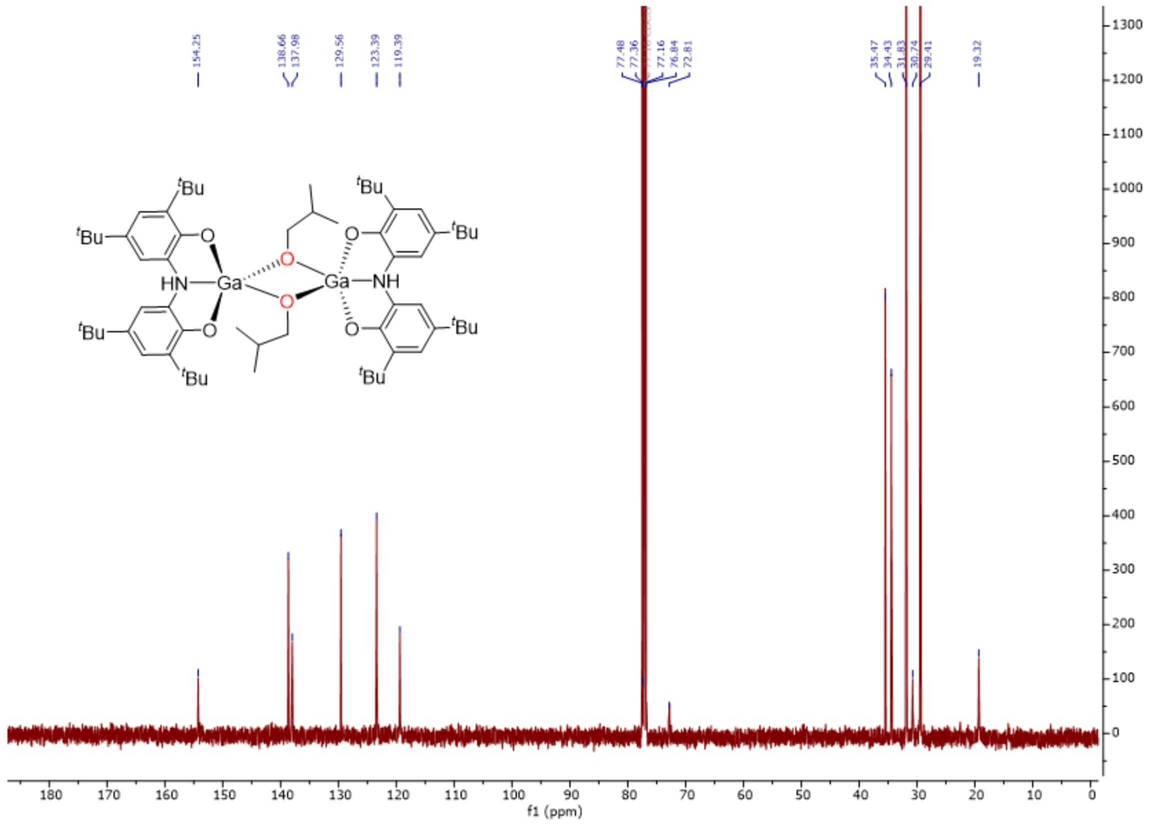

Figure S43. ^{13}C NMR spectrum for compound **6b** in $CDCl_3$.

2.9.3. Preparation of {Ga[ON(H)O](OCH$_2$C$_6$H$_4$OMe)}$_2$ (**6c**)

In accordance with general procedure described above using 4-methoxybenzaldehyde (30 mg, 0.18 mmol) as the substrate to afford **6c** (52 mg, 0.04 mmol, 47% yield). Elemental analysis calculated for $C_{84}H_{112}Ga_2N_2O_8$: C, 71.19%; H, 7.97%; N, 1.98%; found: C, 70.62%; H, 7.88%; N, 1.98%. ^{1}H NMR (400 MHz, CDCl$_3$): δ (ppm) 7.48 (d, $^{3}J_{H–H}$ = 8.2 Hz, 4H, Ar-*H*), 7.14 (d, $^{4}J_{H–H}$ = 2.2 Hz, 4H; Ar-*H*), 7.08 (d, $^{4}J_{H–H}$ = 2.3 Hz, 4H; Ar-*H*), 6.65 (d, $^{3}J_{H–H}$ = 8.1 Hz, 4H, Ar-*H*), 5.15 (s, 4H; C*H*$_2$), 4.00 (s, 2H; N*H*), 3.68 (s, 6H; OC*H*$_3$), 1.39 (s, 36H; C(C*H*$_3$)$_3$), 1.27 (s, 36H; C(C*H*$_3$)$_3$). ^{13}C{^{1}H} NMR (101 MHz, CDCl$_3$): δ (ppm) 159.46 (Ar-*C*), 154.02 (Ar-*C*), 138.32 (Ar-*C*), 137.60 (Ar-*C*), 132.58 (Ar-*C*), 129.71 (Ar-*C*), 128.20 (Ar-*C*), 122.98 (Ar-*C*), 119.15 (Ar-*C*), 114.26 (Ar-*C*), 67.22 (O*C*H$_2$), 55.22 (O*C*H$_3$), 35.53 (*C*(CH$_3$)$_3$), 34.28 (*C*(CH$_3$)$_3$), 31.73 (C(*C*H$_3$)$_3$), 29.45 (C(*C*H$_3$)$_3$).

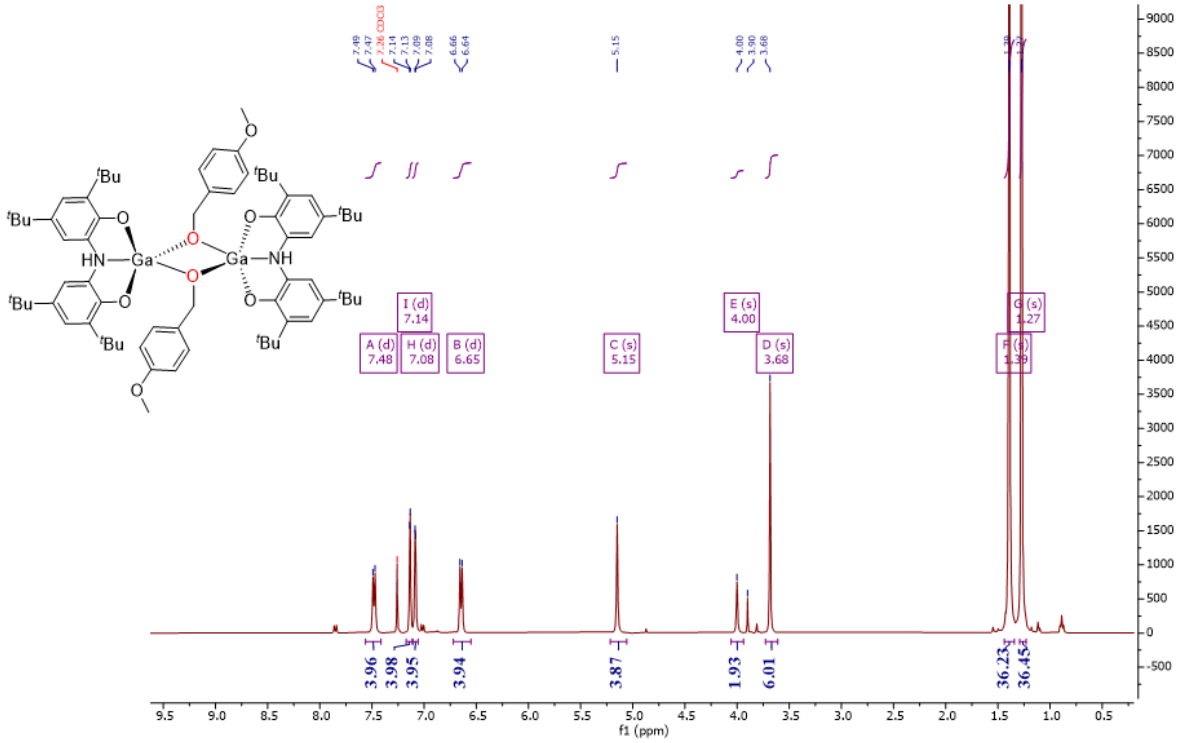

Figure S44. ^{1}H NMR spectrum for compound **6c** in CDCl$_3$.

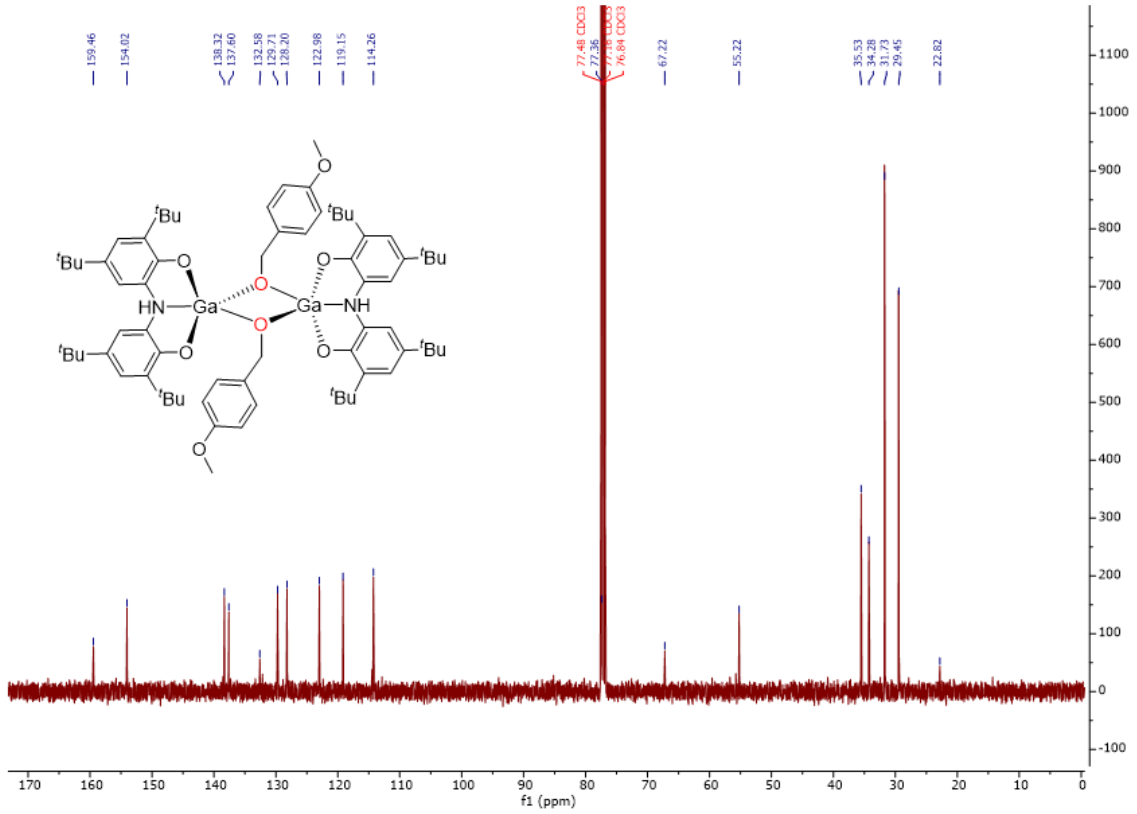

Figure S45. ^{13}C NMR spectrum for compound **6c** in $CDCl_3$.

2.9.4. Preparation of {Ga[ON(H)O](OCH$_2$C$_6$H$_4$-4-Br)}$_2$ (**6d**)

In accordance with the general procedure described above using 4-bromobenzaldehyde (33 mg, 0.18 mmol) to afford **6d** (70 mg, 0.051 mmol, 58% yield). Elemental analysis calculated for $C_{70}H_{94}Br_2Ga_2N_2O_6$: C, 61.88%; H, 6.97%; N, 2.06%; C, 61.43%; H, 7.22%; N, 2.19%. ^{1}H NMR (500 MHz, $CDCl_3$): δ (ppm) 7.44 (d, $^{3}J_{H-H}$ = 8.1 Hz, 4H, Ar-*H*), 7.29 (d, $^{3}J_{H-H}$ = 8.1 Hz, 4H, Ar-*H*), 7.20 (d, $^{4}J_{H-H}$ = 2.4 Hz, 4H; Ar-*H*), 7.15 (d, $^{4}J_{H-H}$ = 2.4 Hz, 4H; Ar-*H*), 5.21 (s, 4H, C*H*$_2$), 4.02 (s, 2H; N*H*), 1.40 (s, 36H; C(C*H*$_3$)$_3$), 1.32 (s, 36H; C(C*H*$_3$)$_3$). ^{13}C{^{1}H} NMR (126 MHz, $CDCl_3$): δ (ppm) 153.73 (Ar-*C*), 139.18 (Ar-*C*), 138.98 (Ar-*C*), 137.81 (Ar-*C*), 132.07 (Ar-*C*), 129.40 (Ar-*C*), 128.17 (Ar-*C*), 123.38 (Ar-*C*), 122.00 (Ar-*C*), 119.05 (Ar-*C*), 66.85 (O*C*H$_2$), 35.53 (*C*(CH$_3$)$_3$), 34.34 (*C*(CH$_3$)$_3$), 29.71 (C(*C*H$_3$)$_3$), 29.40 (C(*C*H$_3$)$_3$).

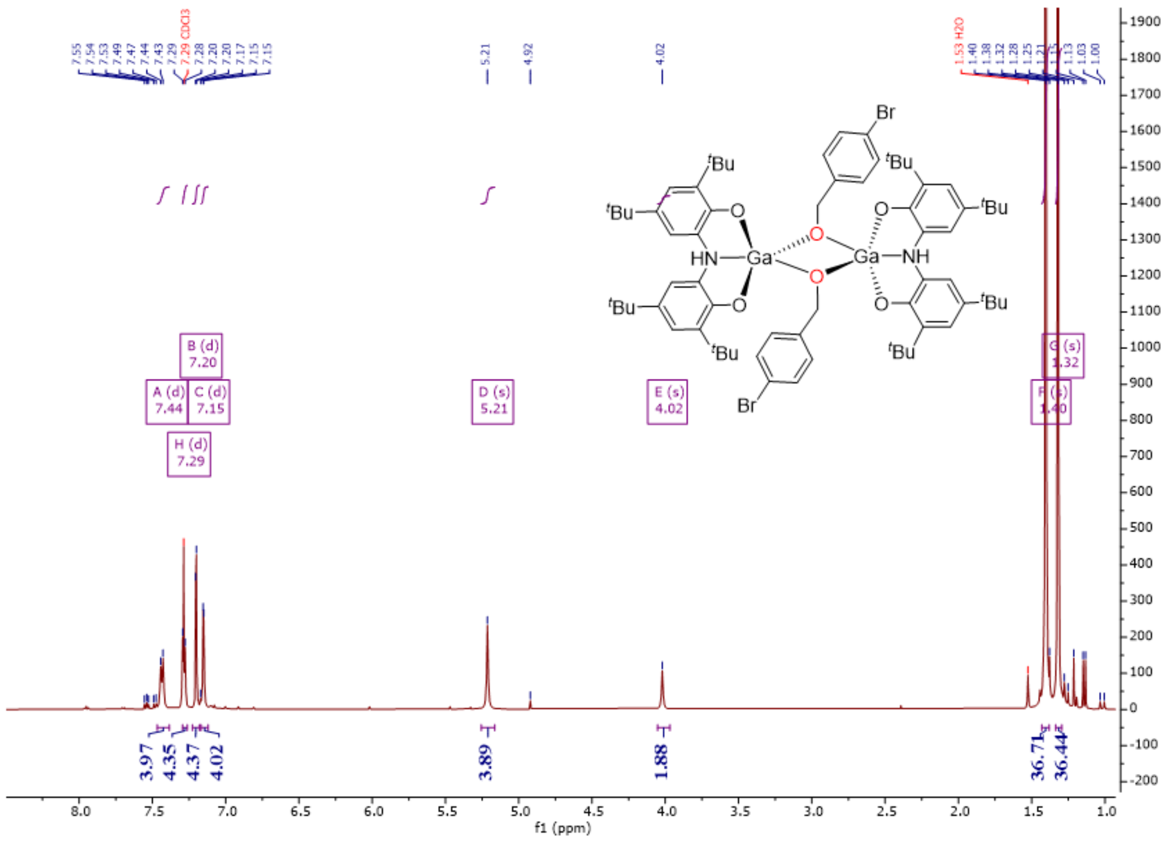

Figure S46. ^{1}H NMR spectrum for compound **6d** in $CDCl_3$.

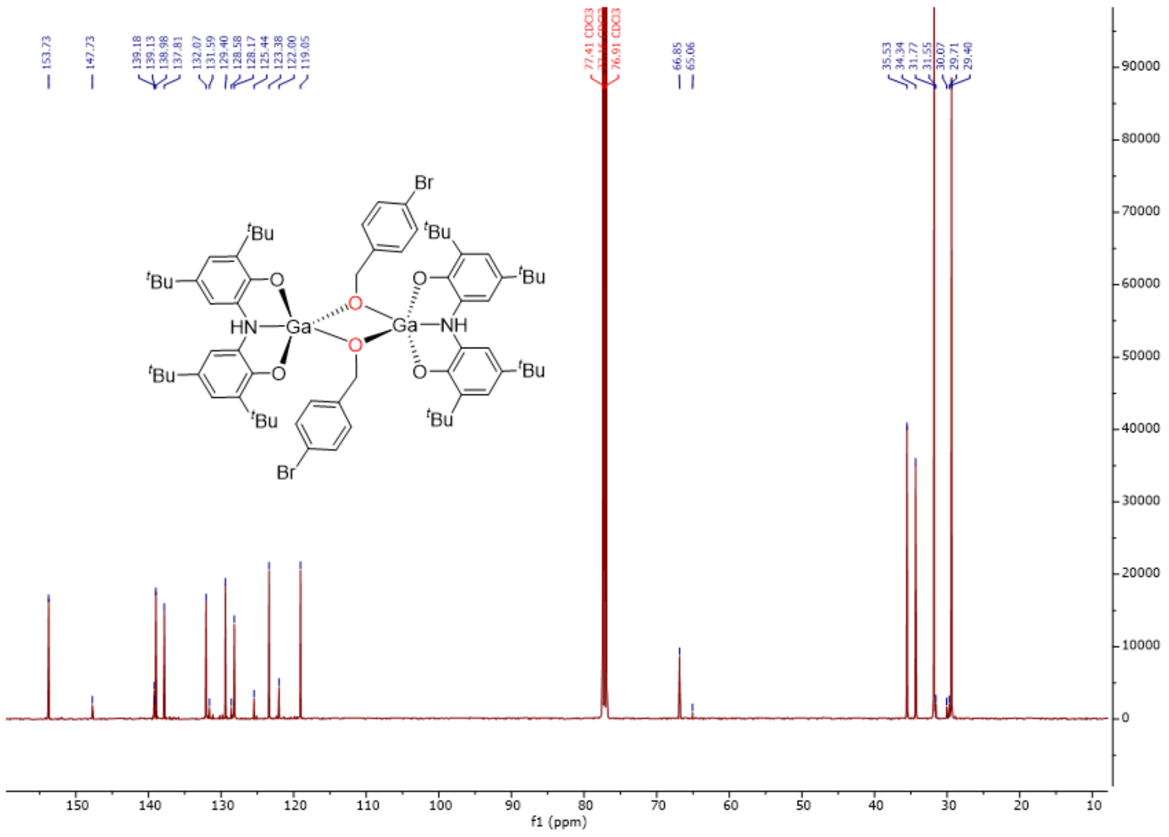

Figure S47. ^{13}C NMR spectrum for compound **6d** in $CDCl_3$.

2.9.5. Preparation of {Ga[ON(H)O](OCH$_2$C$_6$H$_2$Me$_3$)}$_2$ (**6e**)

In accordance with the general procedure described above using 2,4,6-trimethylbenzaldehyde (27 mg, 0.18 mmol) to give afford **6e** (41 mg, 0.032 mmol, 36% yield). Elemental Analysis calculated for $C_{76}H_{108}Ga_2N_2O_6$: C, 71.03%; H, 8.47%; N, 2.18%; found: C, 71.02%; H, 8.62%; N, 2.30%. ^{1}H NMR (500 MHz, CDCl$_3$): δ (ppm) 7.10 (d, $^4J_{H–H}$ = 2.3 Hz, 4H; Ar-*H*), 6.97 (d, $^4J_{H–H}$ = 2.3 Hz, 4H; Ar-*H*), 6.78 (s, 4H; Ar-*H*), 5.35 (s, 4H; C*H*$_2$), 2.49 (s, 6H; C$_6$H$_2$C*H*$_3$), 2.18 (s, 12H; C$_6$H$_2$(C*H*$_3$)$_2$), 1.39 (s, 36H; C(C*H*$_3$)$_3$), 1.31 (s, 36H; C(C*H*$_3$)$_3$). ^{13}C{^{1}H} NMR (126 MHz, CDCl$_3$): δ (ppm) 153.87 (Ar-*C*), 139.16 (Ar-*C*), 138.16 (Ar-*C*), 137.89 (Ar-*C*), 136.97 (Ar-*C*), 133.56 (Ar-*C*), 130.17 (Ar-*C*), 130.04 (Ar-*C*), 122.34 (Ar-*C*), 119.08 (Ar-*C*), 62.98 (O*C*H$_2$), 35.41 (*C*(CH$_3$)$_3$), 34.25 (*C*(CH$_3$)$_3$), 31.87 (C(*C*H$_3$)$_3$), 29.35 (C(*C*H$_3$)$_3$), 21.46 (C$_6$H$_2$*C*H$_3$), 19.13 (C$_6$H$_2$*C*H$_3$).

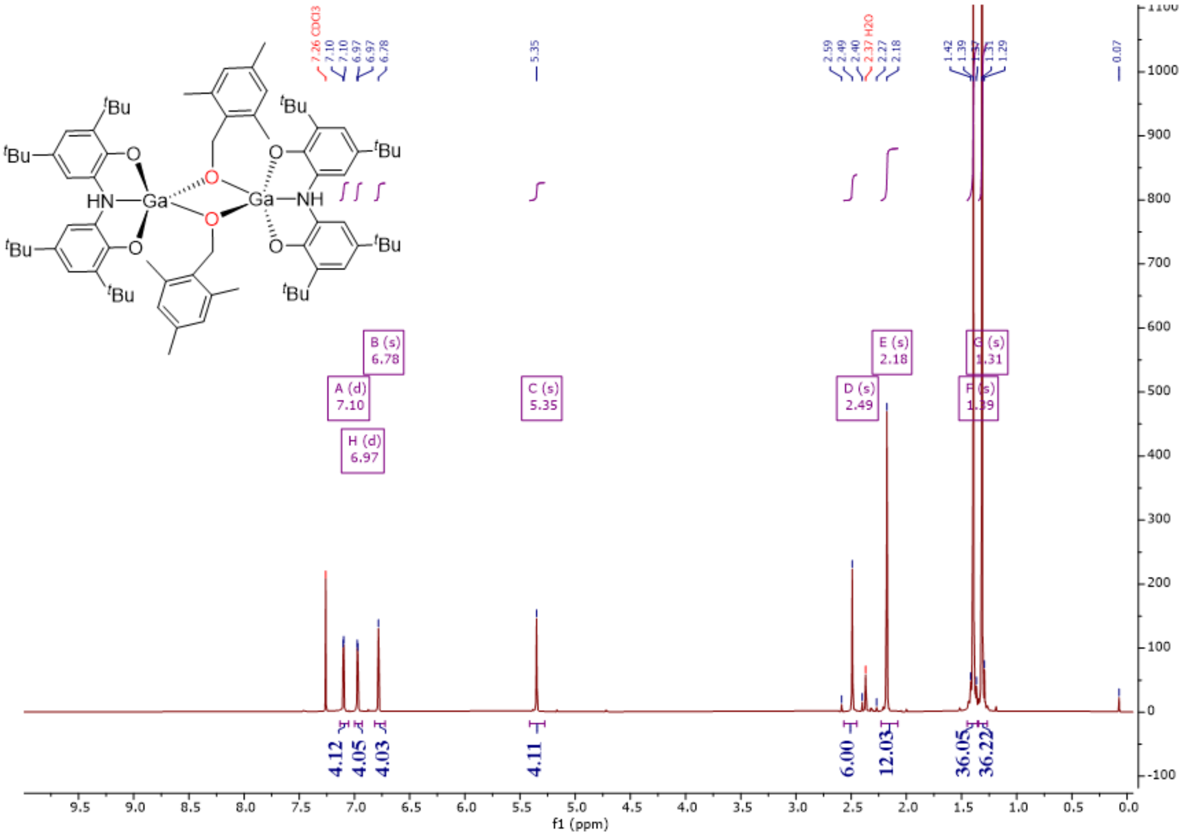

Figure S48. ^{1}H NMR spectrum for compound **6e** in CDCl$_3$.

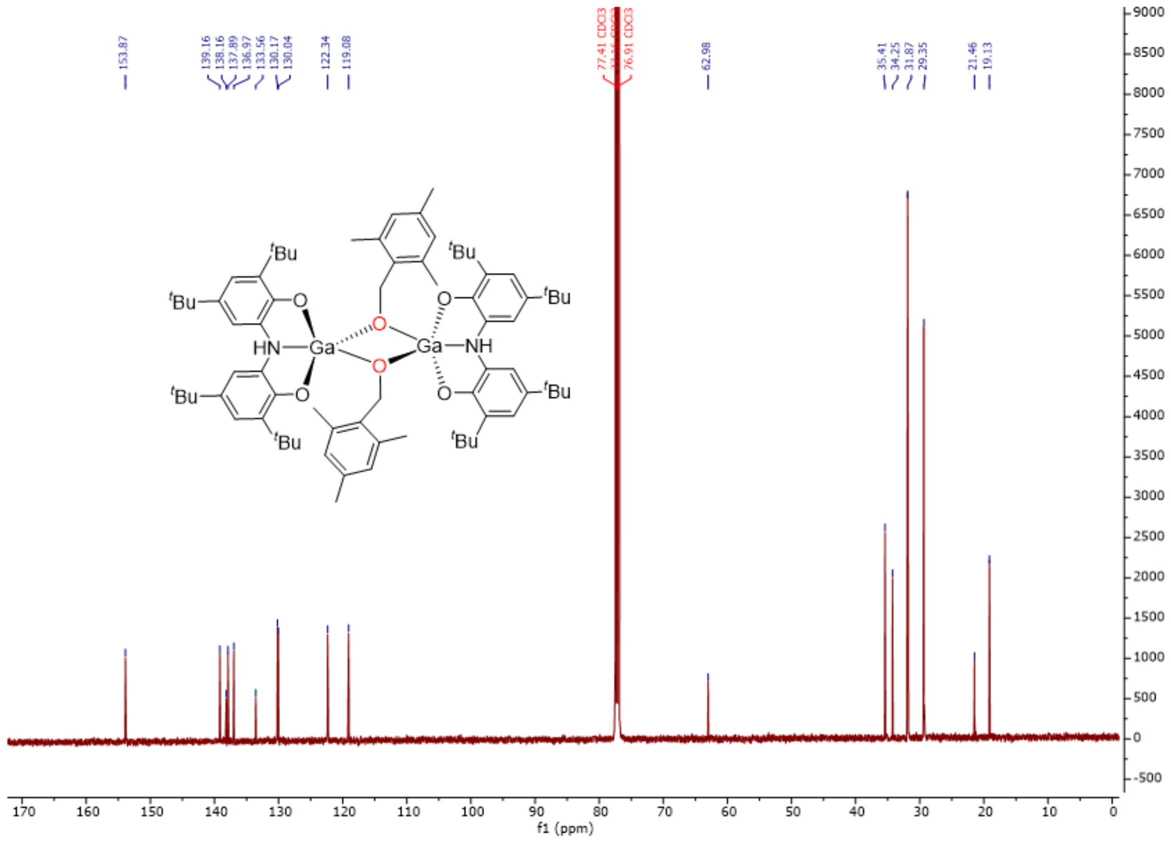

Figure **S49.** ^{13}C NMR spectrum for compound **6e** in $CDCl_3$.

2.9.6. Preparation of {Ga[ON(H)O](OCH(Ph)$_2$)}$_2$ (**7a**)

In accordance with general procedure described above using benzophenone (33 mg, 0.18mmol) to afford **7a** (86 mg, 0.064 mmol, 72% yield). Elemental Analysis calculated for $C_{82}H_{104}Ga_2N_2O_6$: C, 72.78%; H, 7.75%; N, 2.07%; found: C, 72.26%; H, 7.75%; N, 2.30%. ^{1}H NMR (500 MHz, $CDCl_3$): δ (ppm) 7.48–7.41 (m, 8H; Ar-*H*), 7.11–6.99 (m, 16H; Ar-*H*), 6.86 (d, $^4J_{H\text{–}H}$ = 2.3 Hz, 4H; Ar-*H*), 6.71 (s, 2H; OC*H*), 3.75 (s, 2H; N*H*), 1.22 (s, 36H; C(C*H*$_3$)$_3$), 1.19 (s, 36H; C(C*H*$_3$)$_3$). ^{13}C{^{1}H} NMR (126 MHz, $CDCl_3$): δ (ppm) 153.28 (Ar-*C*), 142.48 (Ar-*C*), 138.24 (Ar-*C*), 137.59 (Ar-*C*), 129.34 (Ar-*C*), 128.58 (Ar-*C*), 127.72 (Ar-*C*), 127.14 (Ar-*C*), 122.76 (Ar-*C*), 119.01 (Ar-*C*), 77.86 (O*C*H), 35.13 (*C*(CH$_3$)$_3$), 34.20 (*C*(CH$_3$)$_3$), 31.76 (C(*C*H$_3$)$_3$), 29.13 (C(*C*H$_3$)$_3$).

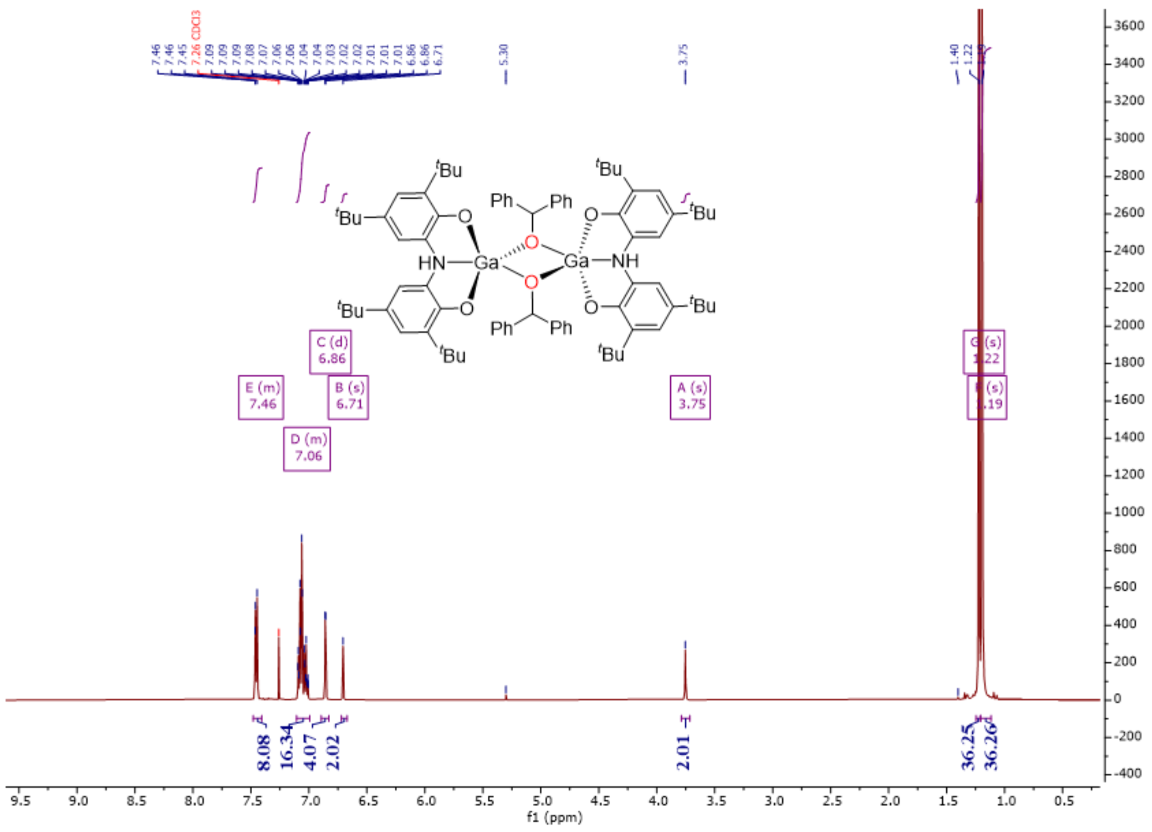

Figure S50. ^{1}H NMR spectrum for compound **7a** in $CDCl_3$.

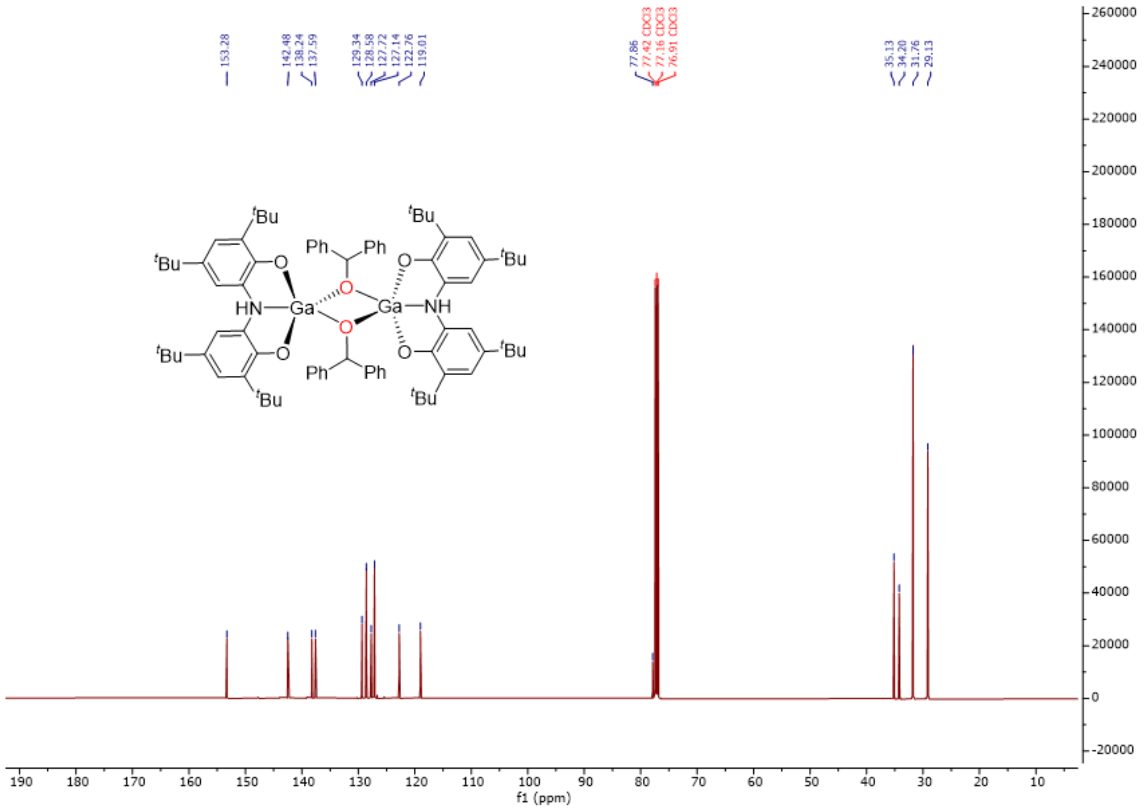

Figure S51. ^{13}C NMR spectrum for compound **7a** in $CDCl_3$.

2.9.7. Preparation of {Ga[ON(H)O](OCH(Me)$_2$)}$_2$ (**7b**)

In accordance with the general procedure described above using acetone (13 μL, 0.18 mmol) to give **7b** (64 mg, 0.058 mmol, 65% yield). Elemental Analysis calculated for $C_{62}H_{96}Ga_2N_2O_6$: C, 67.40%; H, 8.76%; N, 2.54%; found: C, 66.81%; H, 8.78%; N, 2.68%. ^{1}H NMR (500 MHz, CDCl$_3$): δ (ppm) 7.59 (d, $^4J_{H-H}$ = 2.4 Hz, 4H; Ar-*H*), 7.23 (d, $^4J_{H-H}$ = 2.4 Hz, 4H; Ar-*H*), 5.02 (s, 2H; N*H*), 4.39 (sept, $^3J_{H-H}$ = 6.3 Hz, 2H; C*H*(CH$_3$)$_2$), 1.43 (d, $^3J_{H-H}$ = 6.3 Hz, 12H; CH(C*H*$_3$)$_2$), 1.40 (s, 36H; C(C*H*$_3$)$_3$), 1.34 (s, 36H; C(C*H*$_3$)$_3$). ^{13}C{^{1}H} NMR (101 MHz, CDCl$_3$): δ (ppm) 154.16 (Ar-*C*), 138.51 (Ar-*C*), 138.02 (Ar-*C*), 129.73 (Ar-*C*), 123.42 (Ar-*C*), 119.58 (Ar-*C*), 69.59 (O*C*H(CH$_3$)$_2$), 35.49 (*C*(CH$_3$)$_3$), 34.42 (*C*(CH$_3$)$_3$), 31.84 (C(*C*H$_3$)$_3$), 29.75 (C(*C*H$_3$)$_3$), 25.38 (OCH(*C*H$_3$)$_2$).

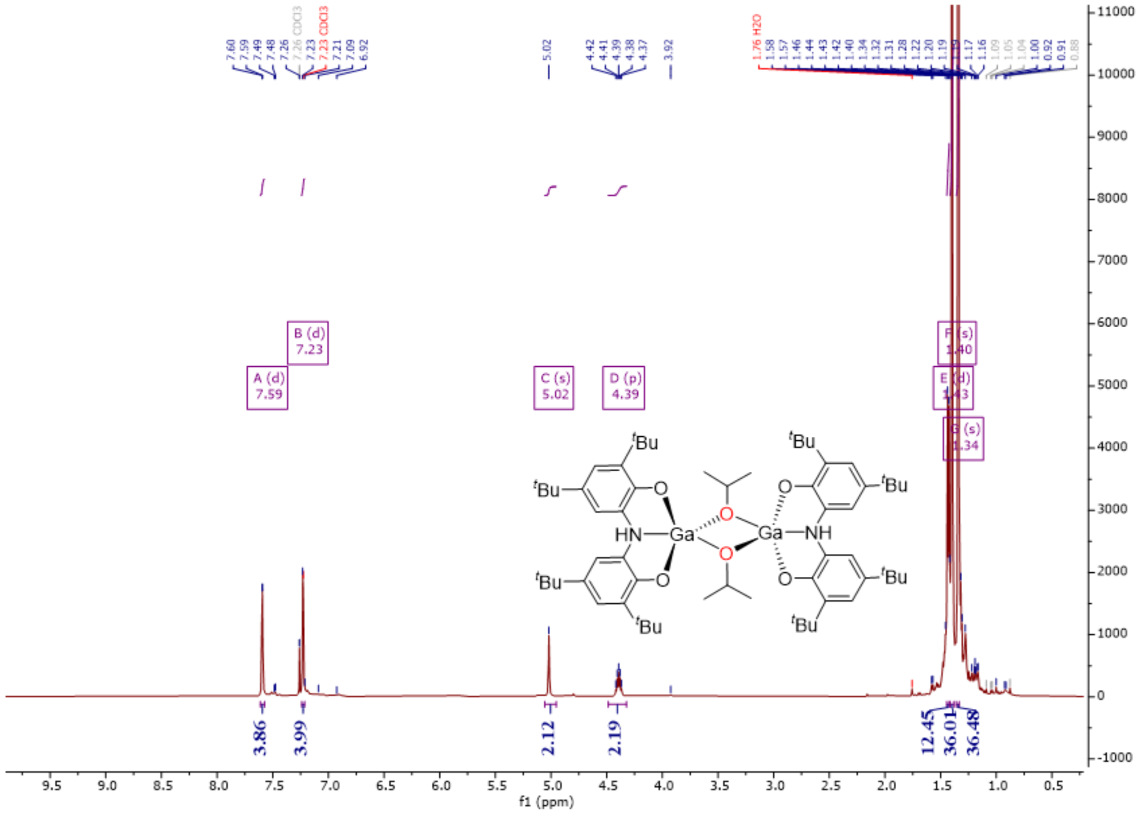

Figure S52. ^{1}H NMR spectrum for compound **7b** in CDCl$_3$.

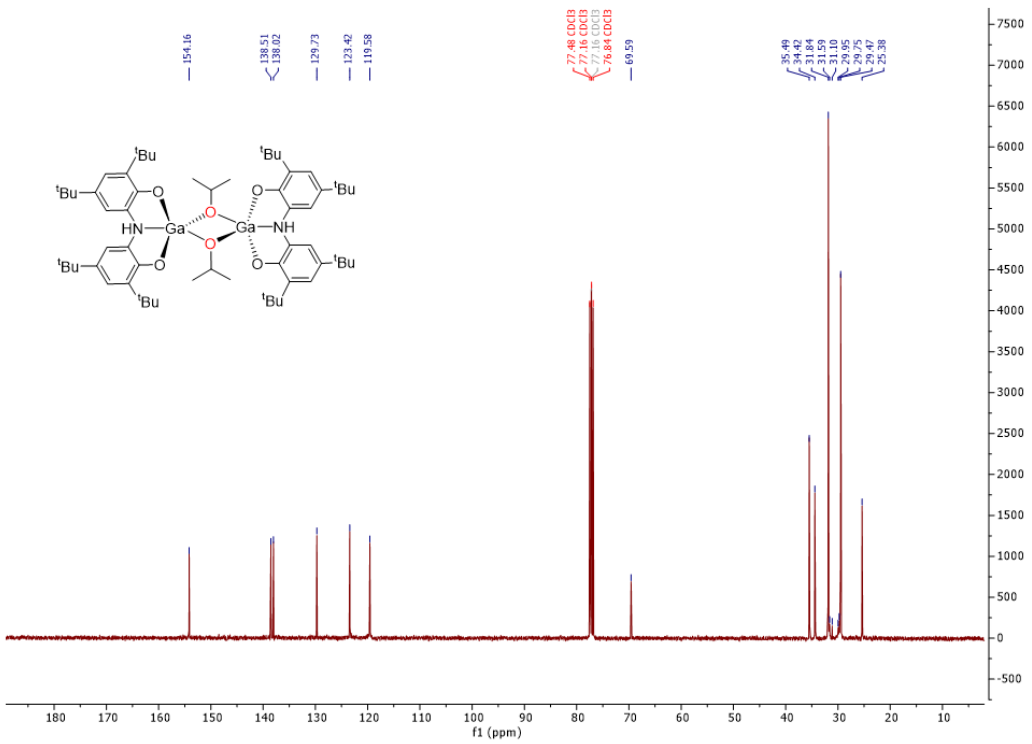

Figure S53. ^{13}C NMR spectrum for compound **7b** in $CDCl_3$.

2.9.8. Preparation of {Ga[ON(H)O][OCH(CH_3)(C_6H_4-4-OMe)]} (**7c**)

In accordance with general procedure described above using 4'-methoxyacetophenone (27 mg, 0.18 mmol) to afford **7c** (90 mg, 0.070 mmol, 79% yield). Elemental Analysis calculated for $C_{74}H_{104}Ga_2N_2O_8$: C, 68.95%; H, 8.13%; N, 2.17%; found: C, 69.06%; H, 8.01%; N, 2.23%. ^{1}H NMR (500 MHz, $CDCl_3$): δ (ppm) 7.63 (overlapped singlets, 4H; Ar-*H*), 7.12 (m, 6H; Ar-*H*), 7.03 (d, $^{4}J_{H–H}$ = 2.3 Hz, 2H, Ar-*H*), 6.70–6.57 (m, 4H; Ar-*H*), 5.46 (q, $^{3}J_{H–H}$ = 6.4 Hz, 2H; OC*H*CH_3), 4.03 (s, 1H; N*H*), 3.96 (s, 1H; N*H*), 3.63 (s, 6H; OCH_3), 3.62 (s, 6H; OCH_3), 1.79 (m, 6H; OCHCH_3), 1.41 (overlapped singlets, 36H; C(CH_3)$_3$), 1.27 (overlapped singlets, 36H; C(CH_3)$_3$). $^{13}C\{^{1}H\}$ NMR (126 MHz, $CDCl_3$): δ (ppm) 159.03 (overlapped singlets, Ar-*C*), 154.16–153.38 (overlapped singlets, Ar-*C*), 138.42–137.90 (overlapped singlets, Ar-*C*), 137.60–137.01 (overlapped singlets, Ar-*C*), 129.62 (overlapped singlets, Ar-*C*), 129.26 (overlapped singlets, Ar-*C*), 128.40–127.62 (overlapped singlets, Ar-*C*), 126.97 (overlapped singlets, Ar-*C*), 122.85 (overlapped singlets, Ar-*C*), 119.98 – 117.95 (overlapped singlets, Ar-

C), 114.10 (s, Ar-*C*), 72.14 (overlapped singlets, O*C*H), 55.12 (overlapped singlets, O*C*H_3), 35.45 (overlapped singlets, *C*(CH_3)$_3$), 34.25 (overlapped singlets, *C*(CH_3)$_3$), 31.74 (overlapped singlets, C(*C*H_3)$_3$), 29.42 (overlapped singlets, C(*C*H_3)$_3$), 24.04 (overlapped singlets, O*C*HCH_3).

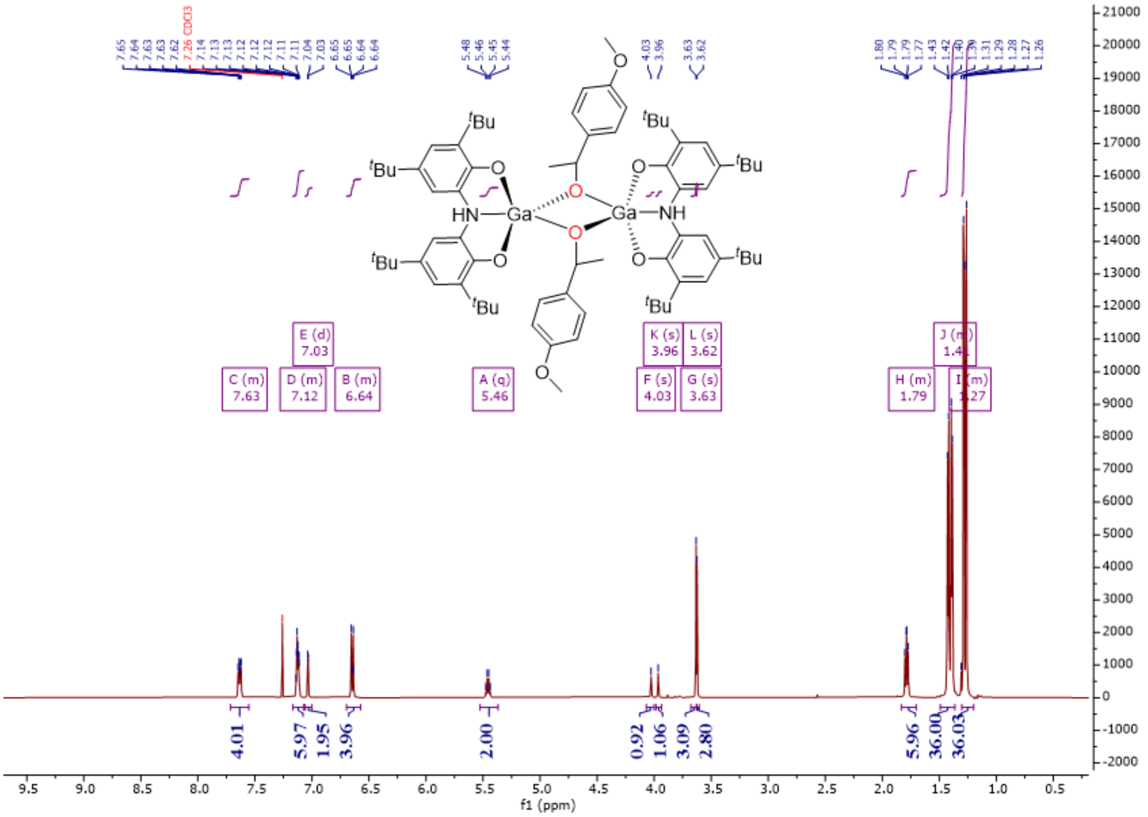

Figure S54. ^{1}H NMR spectrum for compound **7c** in $CDCl_3$.

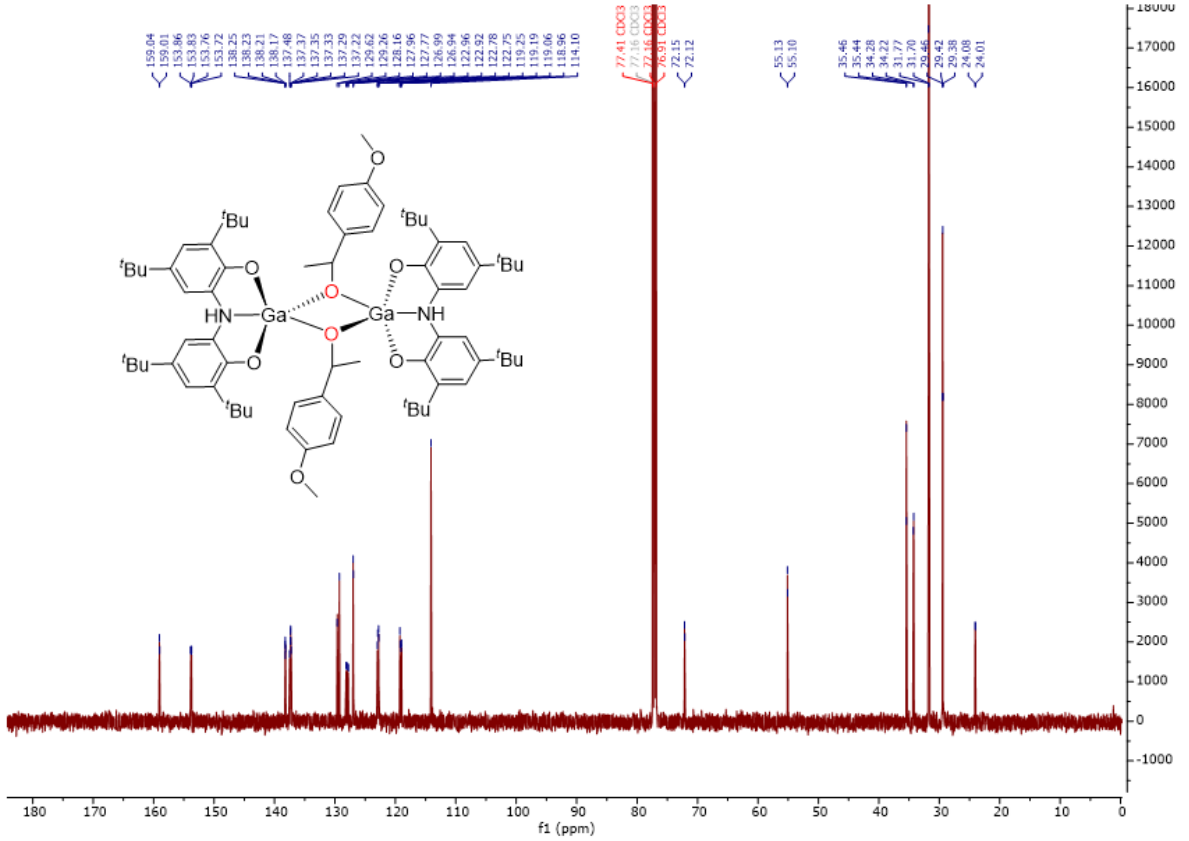

Figure S55. ^{13}C NMR spectrum for compound **7c** in $CDCl_3$.

2.9.9. Preparation of {Ga[ON(H)O][OCH(CH_3)(C_6H_4-4-Br)]}$_2$ (**7d**)

In accordance with the general procedure described above using 4-bromoacetophenone (36 mg, 0.18 mmol) to give **7d** (54 mg, 0.039 mmol, 44% yield). Elemental Analysis calculated for $C_{72}H_{98}Br_2Ga_2N_2O_6$: C, 62.36%; H, 7.12%; N, 2.02%; found: C, 62.10%; H, 7.32%; N, 2.14%. ^{1}H NMR (500 MHz, $CDCl_3$): : δ (ppm) 7.60 – 7.52 (m, 4H; Ar-*H*), 7.25 (d, $^{4}J_{H-H}$ = 1.6 Hz, 2H; Ar-*H*), 7.23 (d, $^{4}J_{H-H}$ = 1.6 Hz, 2H; Ar-*H*), 7.16 (d, $^{4}J_{H-H}$ = 2.3 Hz, 1H; Ar-*H*), 7.15 (d, $^{4}J_{H-H}$ = 2.3 Hz, 2H; Ar-*H*), 7.14 (d, $^{4}J_{H-H}$ = 2.3 Hz, 1H; Ar-*H*), 7.13 (d, $^{4}J_{H-H}$ = 2.3 Hz, 2H; Ar-*H*), 7.07 (d, $^{4}J_{H-H}$ = 2.3 Hz, 1H; Ar-*H*), 7.06 (d, $^{4}J_{H-H}$ = 2.3 Hz, 1H; Ar-*H*), 5.44 (q, $^{3}J_{H-H}$ = 6.5 Hz, 2H; OC*H*CH_3), 3.92 (s, 1H; N*H*), 3.86 (s, 1H; N*H*), 1.94 – 1.64 (m, 6H, CHC*H*$_3$), 1.46 – 1.21 (overlapped singlets, 72H, C(C*H*$_3$)$_3$). $^{13}C\{^{1}H\}$ NMR (126 MHz, $CDCl_3$): δ (ppm) 153.82–

153.10 (overlapped singlets, Ar-*C*), 144.14 (overlapped singlets, Ar-*C*), 139.07–138.61 (overlapped singlets, Ar-*C*), 137.77–137.30 (overlapped singlets, Ar-*C*), 132.01, 129.36–128.85 (overlapped singlets, Ar-*C*), 128.22–127.68 (overlapped singlets, Ar-*C*), 127.40 (overlapped singlets, Ar-*C*), 123.28 (overlapped singlets, Ar-*C*), 118.97 (overlapped singlets, Ar-*C*), 72.21 (overlapped singlets, O*C*H), 35.46 (overlapped singlets, *C*(CH_3)$_3$), 34.31 (overlapped singlets, *C*(CH_3)$_3$), 31.79 (overlapped singlets, C(*C*H_3)$_3$), 29.38 (overlapped singlets, C(*C*H_3)$_3$), 24.28 (overlapped singlets, CH*C*H_3).

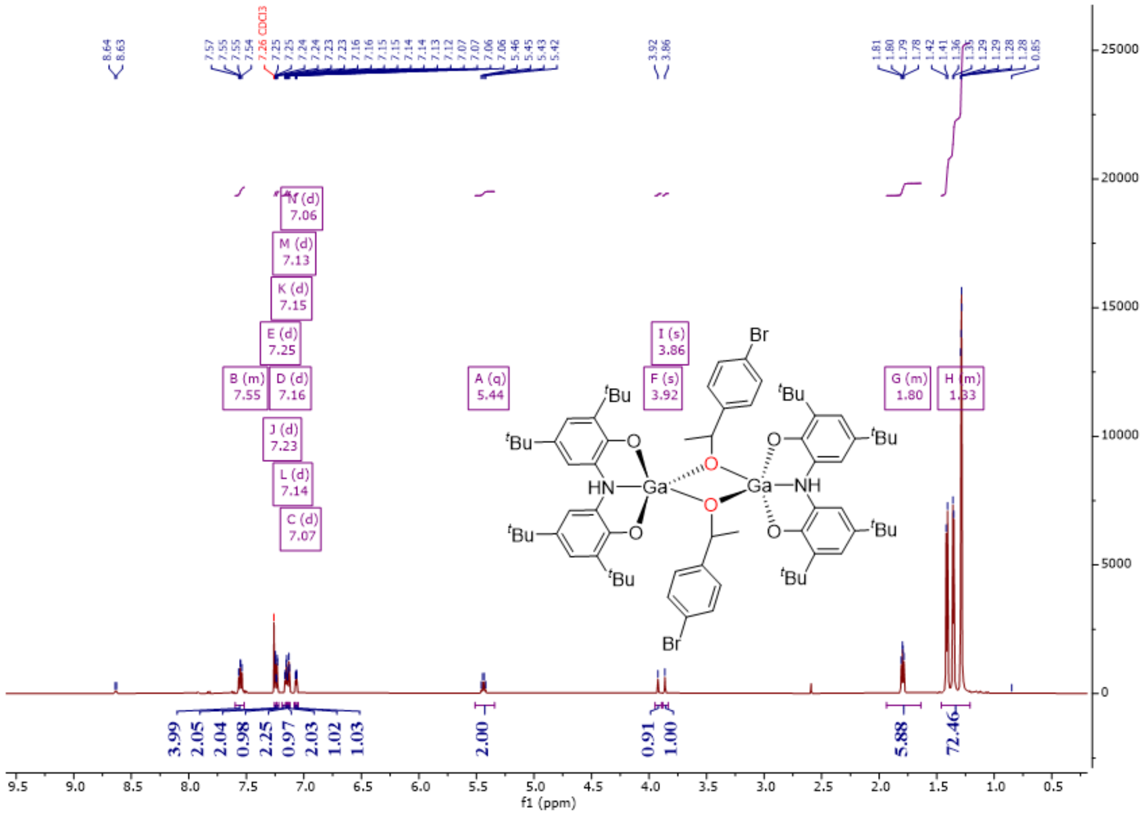

Figure S56. ^{1}H NMR spectrum for compound **7d** in $CDCl_3$.

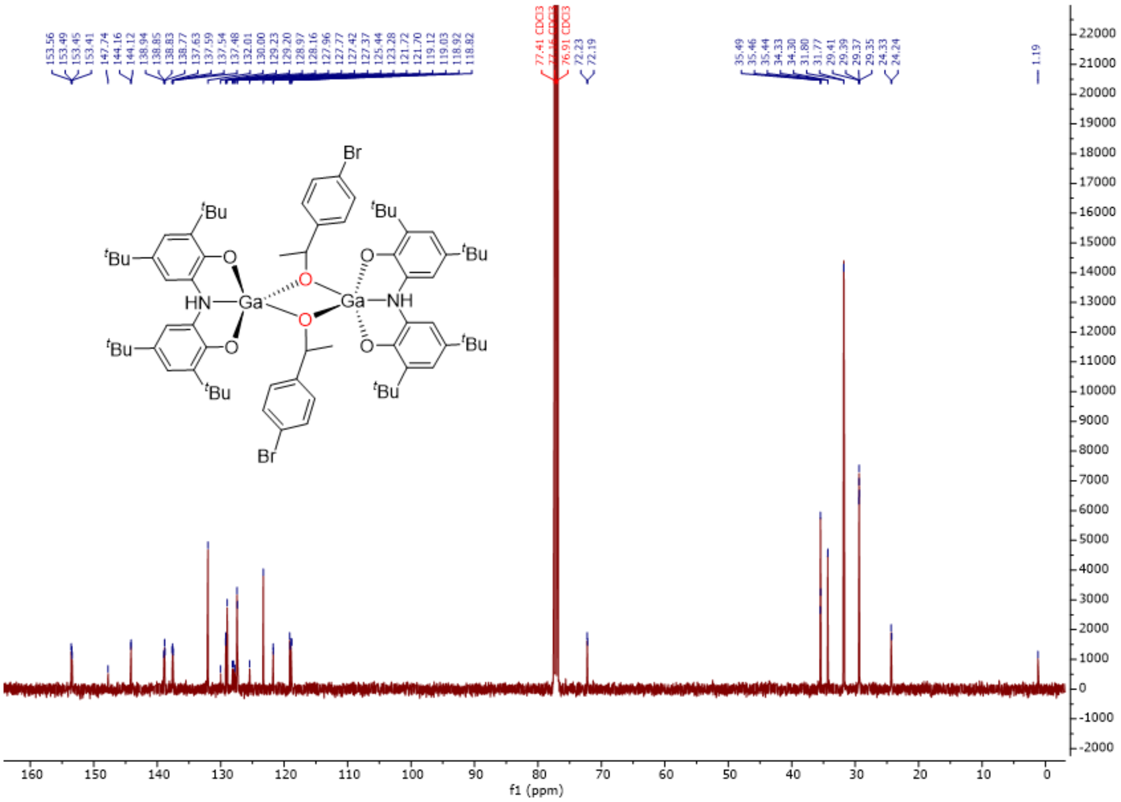

Figure S57. ^{13}C NMR spectrum for compound **7d** in $CDCl_3$.

2.9.10. Preparation of $\{Ga[ON(H)O](OCHC_8H_8)\}_2$ (**7e**)

In accordance with the general procedure described above using 2-indanone (24 mg, 0.18 mmol) to give **7e** (73 mg, 0.058 mmol, 66% yield). Elemental Analysis calculated for $C_{74}H_{100}Ga_2N_2O_6$: C, 70.93%; H, 8.04%; N, 2.24%; found: C, 71.18%; H, 7.72%; N, 2.26%.

^{1}H NMR (500 MHz, $CDCl_3$): δ (ppm) 7.34 (m, 4H; Ar-*H*), 7.17–7.06 (m, 8H; Ar-*H*), 6.85 (d, $^4J_{H-H}$ = 2.3 Hz, 4H; Ar-*H*), 5.33 (m, 2H; OC*H*C_8H_8), 3.66 (s, 2H; N*H*), 3.52 (d, $^3J_{H-H}$ = 7.6 Hz, 2H; OCHCH_2), 3.48 (d, $^3J_{H-H}$ = 7.6 Hz, 2H; OCHCH_2), 3.05 (d, $^4J_{H-H}$ = 1.9 Hz, 2H; OCHCH_2CH_2), 3.01 (d, $^4J_{H-H}$ = 1.9 Hz, 2H; OCHCH_2CH_2),1.39 (s, 36H; C(CH_3)$_3$), 1.29 (s, 36H; C(CH_3)$_3$). ^{13}C{^{1}H} NMR (126 MHz, $CDCl_3$): δ(ppm) 153.90 (Ar-*C*), 141.39 (Ar-*C*), 137.84 (Ar-*C*), 137.24 (Ar-*C*), 129.29 (Ar-*C*), 127.45 (Ar-*C*), 125.88 (Ar-*C*), 122.72 (Ar-*C*), 119.81 (Ar-*C*), 75.40 (O*C*H), 44.03 (O*C*H_2), 35.48 (*C*(CH_3)$_3$), 34.33 (*C*(CH_3)$_3$), 31.84 (C(*C*H_3)$_3$), 29.39 (C(*C*H_3)$_3$).

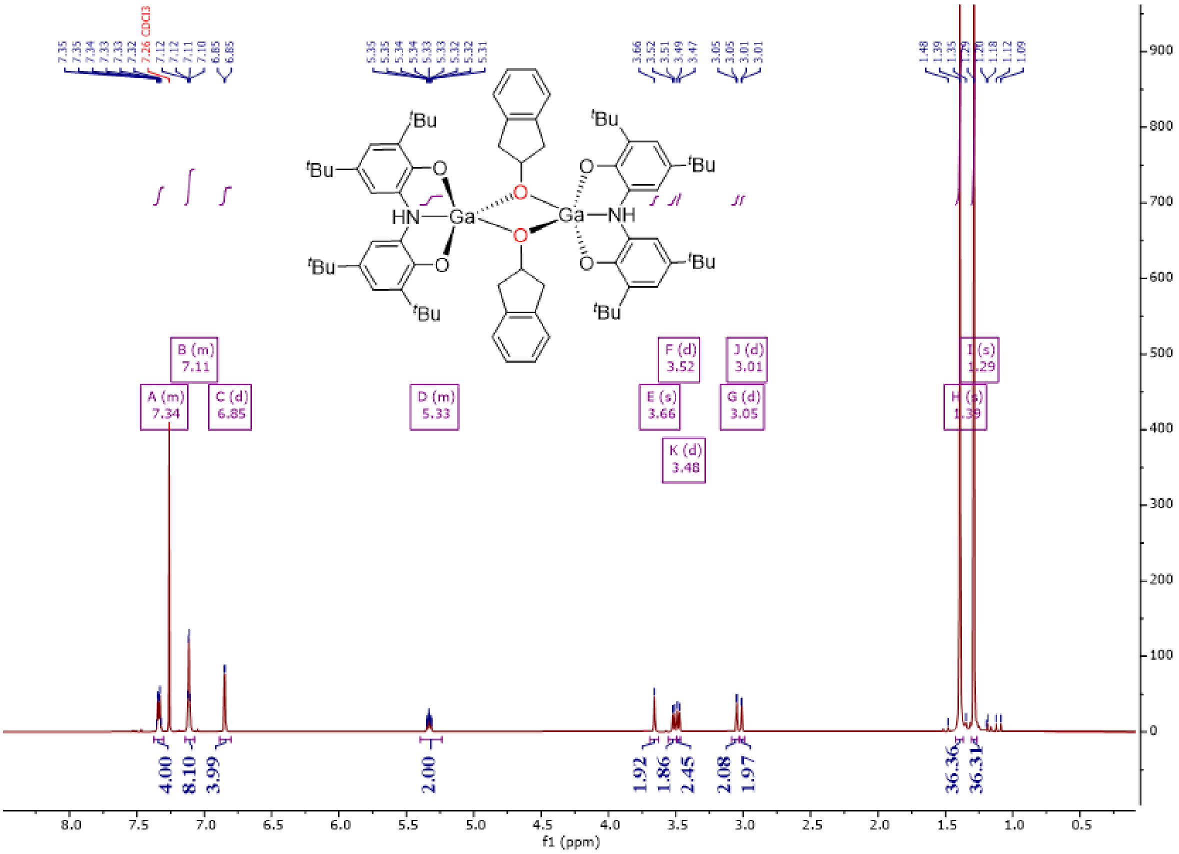

Figure S58. ^{1}H NMR spectrum for compound **7e** in $CDCl_3$.

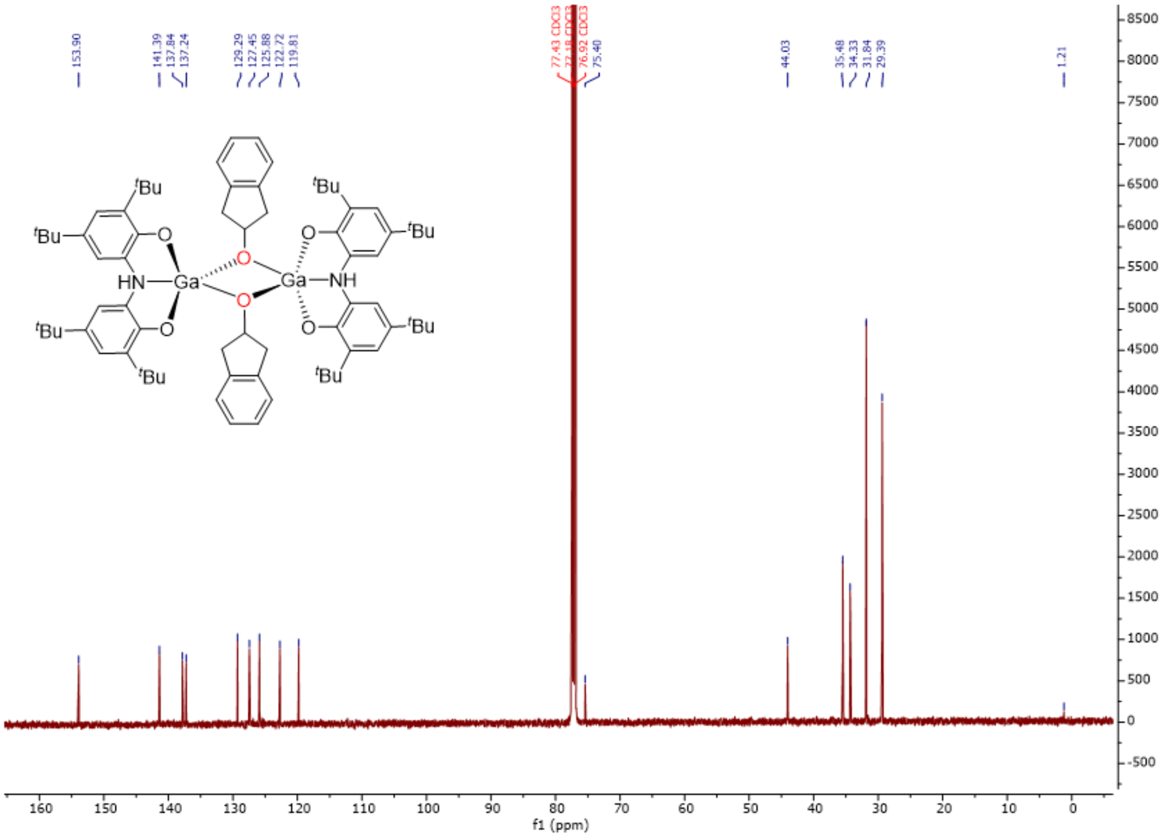

Figure S59. ^{13}C NMR spectrum for compound **7e** in $CDCl_3$.

2.9.11. Preparation of {Ga[ON(H)O][OCH(CH_3)(C_6H_4-4-NO_2)]}$_2$ (**7f**)

In accordance with the general procedure described above using 4′-nitroacetophenone (30 mg, 0.18 mmol) to give **7f**. (64 mg, 0.049 mmol, 55% yield). Elemental Analysis calculated for $C_{72}H_{98}Ga_2N_2O_6$: C, 65.56%; H, 7.49%; N, 4.25%; found: C, 65.35%; H, 7.45%; N, 4.47%. ^{1}H NMR (500 MHz, $CDCl_3$): δ (ppm) 7.96 (m, 4H, Ar-*H*), 7.87 (m, 4H, Ar-*H*), 7.15 (m, 4H, Ar-*H*), 7.09–6.95 (m, 4H, Ar-*H*), 5.58 (q, $^{3}J_{H–H}$ = 6.5 Hz, 2H; OC*H*CH_3), 3.96 (s, 1H; N*H*), 3.94 (s, 1H; N*H*), 1.91 (m, 6H, CHCH_3), 1.39 (overlapped singlets, 36H, C(CH_3)$_3$), 1.22 (overlapped singlets, 36H, C(CH_3)$_3$). ^{13}C{^{1}H} NMR (126 MHz, $CDCl_3$): δ (ppm) 153.23 (overlapped singlets, Ar-*C*), 152.15 (overlapped singlets, Ar-*C*), 147.29 (overlapped singlets, Ar-*C*), 139.29 (overlapped singlets, Ar-*C*), 137.77 (overlapped singlets, Ar-*C*), 128.84 (overlapped singlets, Ar-*C*), 126.54 (overlapped singlets, Ar-*C*), 124.10 (overlapped singlets, Ar-*C*), 123.66 (overlapped singlets, Ar-*C*), 118.89 (overlapped singlets, Ar-*C*), 72.45 (overlapped singlets,

OCH), 35.50 (overlapped singlets, $C(CH_3)_3$), 34.26 (overlapped singlets, $C(CH_3)_3$), 31.61 (overlapped singlets, $C(CH_3)_3$), 29.43 (overlapped singlets, $C(CH_3)_3$), 24.37 (overlapped singlets, $CHCH_3$).

^{1}H NMR (500 MHz, C_6D_6): δ (ppm) 7.86 (d, $^3J_{H–H}$ = 8.6 Hz, 4H; Ar-*H*), 7.42 (d, $^3J_{H–H}$ = 8.4 Hz, 4H; Ar-*H*), 7.37 (d, $^4J_{H–H}$ = 2.3 Hz, 2H; Ar-*H*), 7.35 (d, $^4J_{H–H}$ = 2.3 Hz, 2H; Ar-*H*), 7.29 (d, $^4J_{H–H}$ = 2.3 Hz, 2H; Ar-*H*), 7.24 (d, $^4J_{H–H}$ = 2.3 Hz, 2H; Ar-*H*), 5.13 (q, $^3J_{H–H}$ = 6.6 Hz, 2H; Ar-*H*), 3.90 (s, 2H; N*H*), 1.72 (d, $^3J_{H–H}$ = 6.6 Hz, 6H; $CHCH_3$), 1.58 (s, 18H; $C(CH_3)_3$), 1.55 (s, 18H; $C(CH_3)_3$), 1.33 (s, 18H; $C(CH_3)_3$), 1.28 (s, 18H; $C(CH_3)_3$). The solubility of **7f** in benzene is bad, consequently there is no available ^{13}C NMR spectrum for **7f** in C_6D_6.

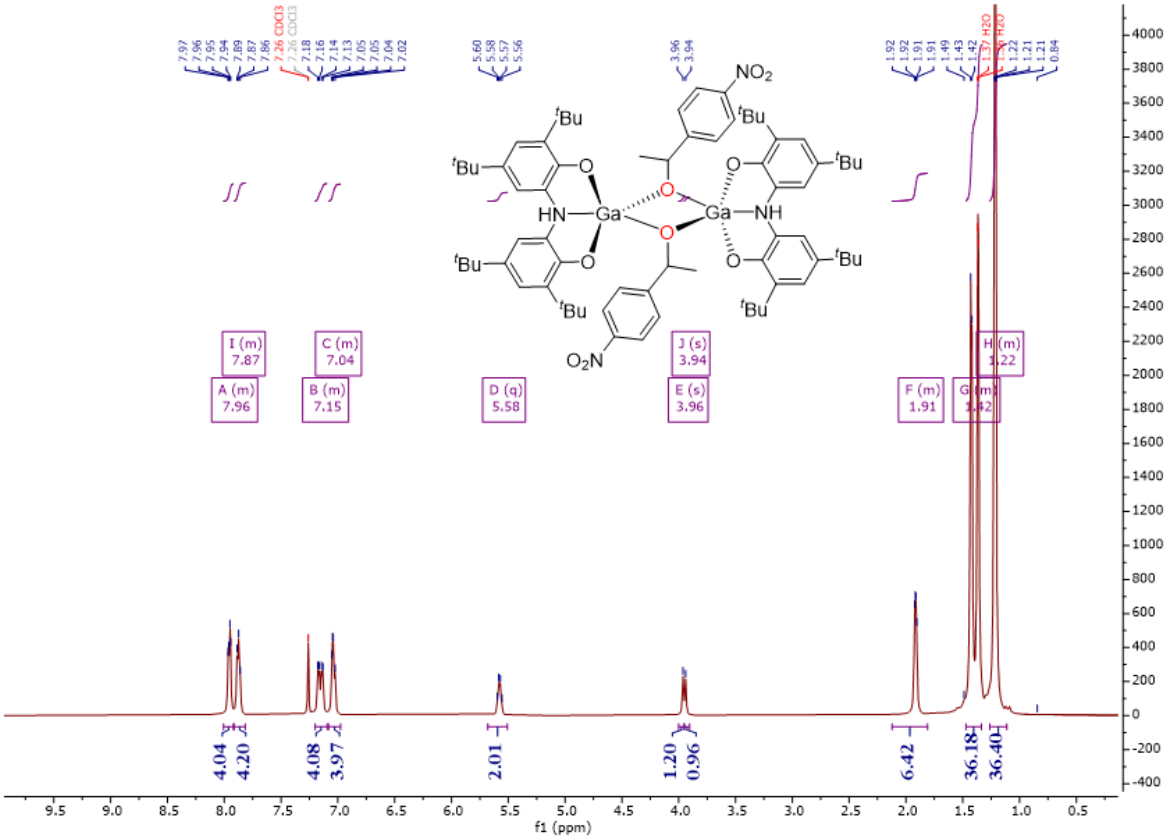

Figure S60. ^{1}H NMR spectrum for compound **7f** in $CDCl_3$.

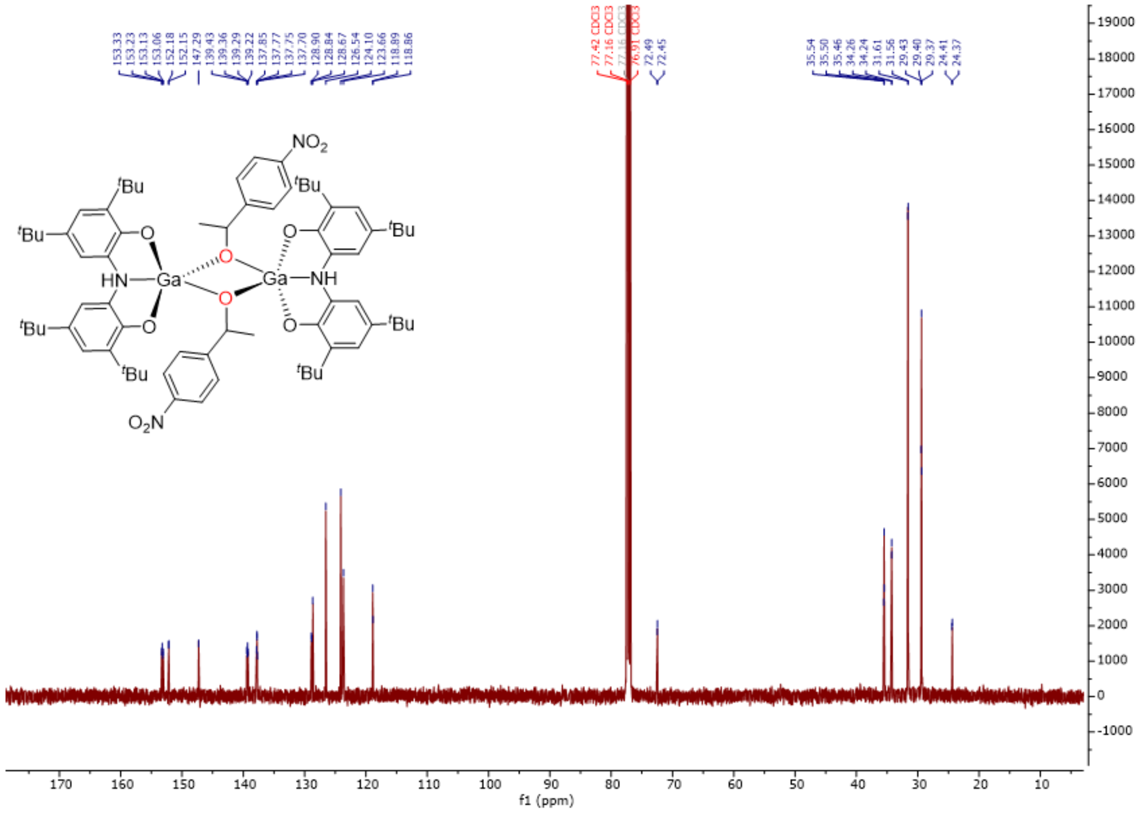

Figure S61. ^{13}C NMR spectrum for compound **7f** in $CDCl_3$.

3. Catalytic hydroboration of aldehydes and ketones

General procedure for preparing stock solutions for the hydroboration of ketones or aldehydes: To a dried ampoule containing the pre-catalyst **4a** (27 mg) or **6a** (30 mg) and hexamethylcyclotrisiloxane (1.110 g) as an internal standard, was added 50 mL of C_6D_6. This gives rise to a stock solution containing 0.0005 mol·L^{-1} of catalyst and 0.1 mol·L^{-1} of hexamethylcyclotrisiloxane.

To the stock solution (500 µL, with 0.00025 mmol **4a** or **6a**), the neat aldehyde or ketone substrate (ca. 0.5 mmol) was added, and neat HBpin (80 µL, 0.55 mmol) was added using a (100 µL) gastight syringe.

3.1. Catalytic hydroboration of aldehydes

Table S3. Reaction conditions: To a stock solution (500 µL C_6D_6, with 0.00025 mmol **6a**) in an NMR tube with a J.Young NMR valve, was added neat aldehyde (ca. 0.5 mmol), then neat HBpin (80 µL, 0.55 mmol). The reaction was monitored by NMR spectroscopy until completed. All reagents were degassed and store under an inert atmosphere once received.

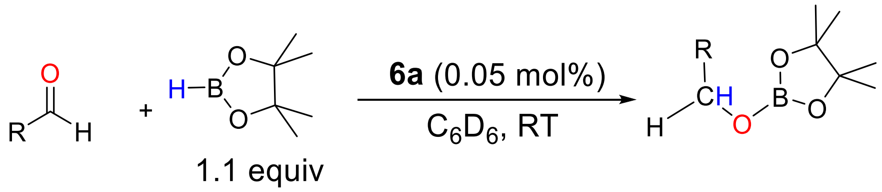

Entry	Aldehyde	Catalyst loading (mol %)	Time (h)	NMR yield (%)
1	Benzaldehyde	0.05	7	>99
2	4-Methoxybenzaldehyde	0.05	3.5	>99
3	4-Bromobenzaldehyde	0.05	12	>99
4	4-Fluorobenzaldehyde	0.05	1	>99
5	4-Methylbenzaldehyde	0.05	1	>99
6	Mesitaldehyde	0.05	2	>99
7	Isobutyraldehyde	0.05	1.5	>99

Reagents: 0.5 mmol aldehyde, 0.55 mmol HBpin (80µl), reaction was performed at room temperature, ^{1}H NMR yields relative to internal standard hexamethylcyclotrisiloxane.

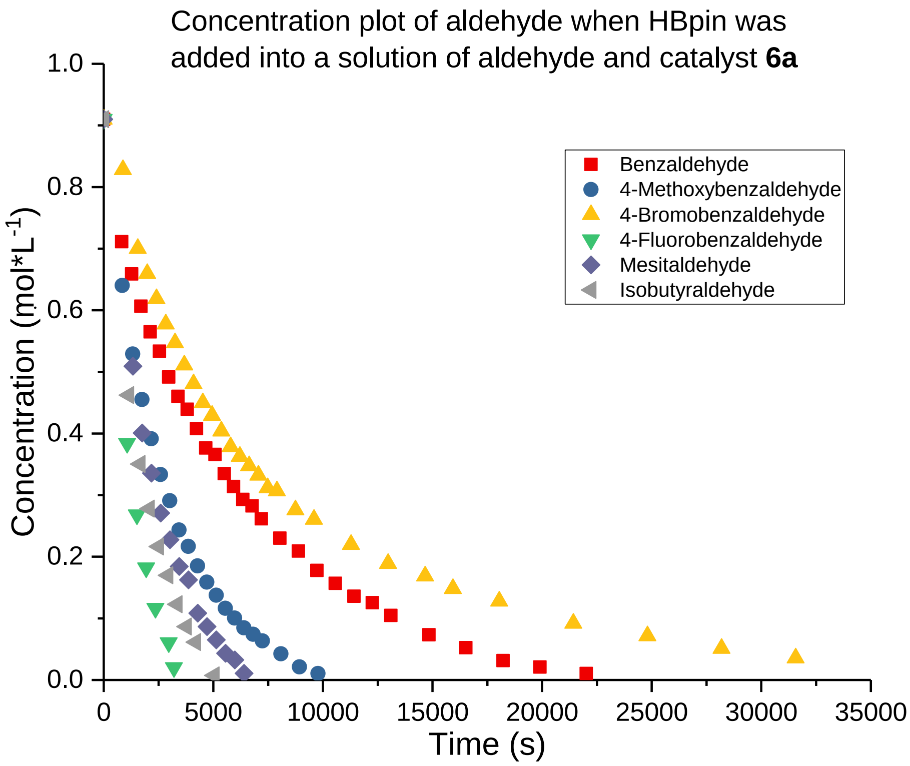

Figure S62. Concentration plot of aldehyde based on the relative integration to internal standard hexamethylcyclotrisiloxane (HMCTS) in C_6D_6. To a stock solution (500 µL, with 0.00025 mmol **6a**), the neat aldehyde (ca. 0.50 mmol) was added, then neat HBpin (80 µL, 0.55 mmol), the solution volume has been approximated to 550 µL.

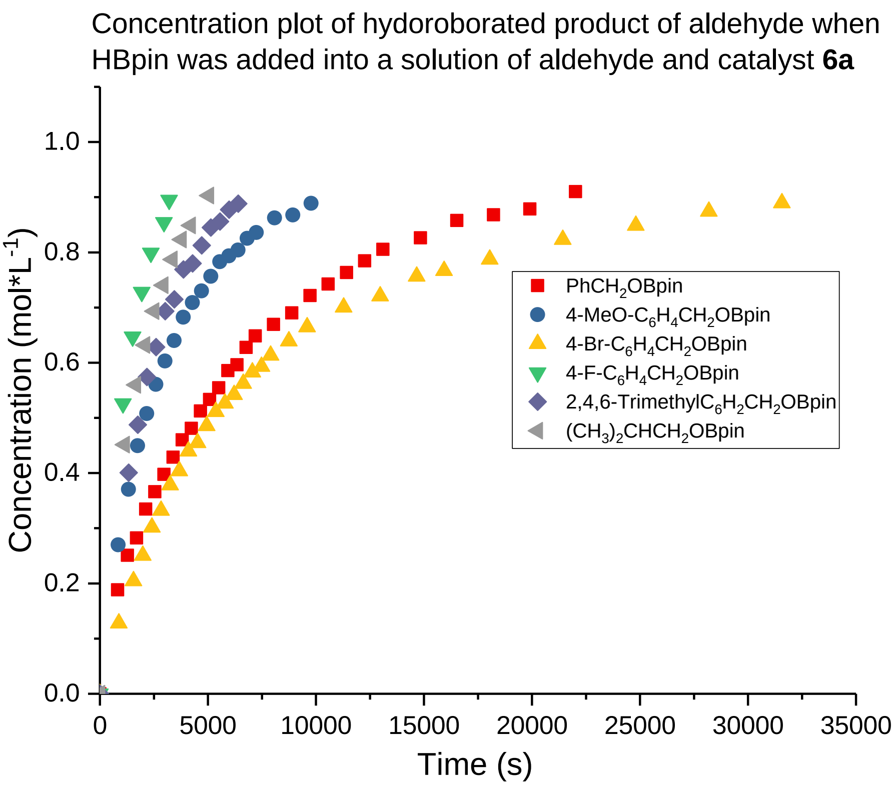

Figure S63. Concentration plot of hydroborated product of aldehyde based on the relative integration to internal standard hexamethylcyclotrisiloxane (HMCTS) in C_6D_6. To a stock solution (500 µL, with 0.00025 mmol **6a**), the neat aldehyde (ca. 0.50 mmol) was added, then neat HBpin (80 µL, 0.55 mmol), the solution volume has been approximated to 550 µL.

3.2. Catalytic hydroboration of ketones

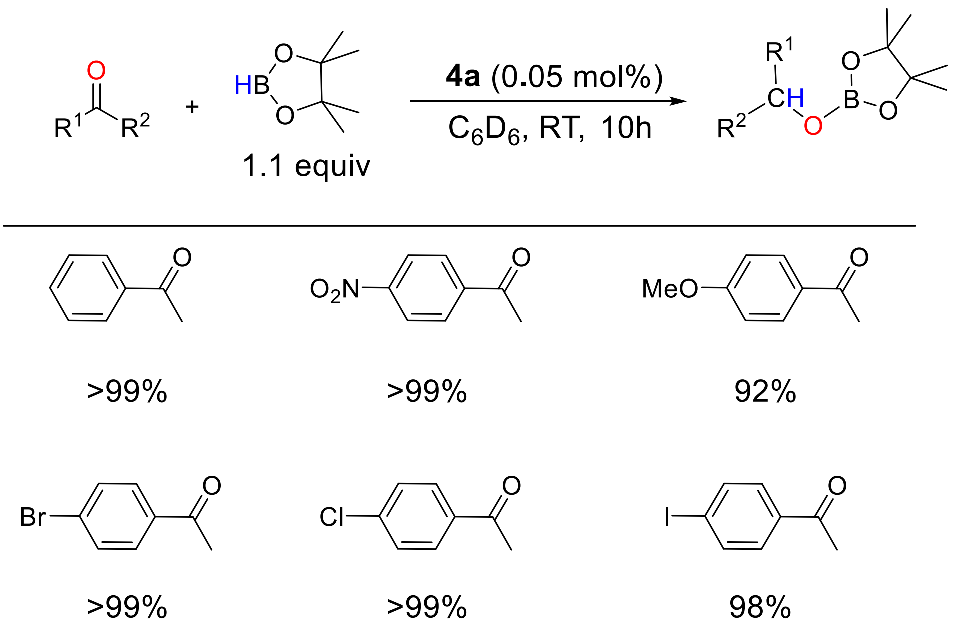

Figure S64. Reaction conditions: To a stock solution (500 µL C_6D_6, with 0.00025 mmol **4a**) was added neat ketone (ca. 0.5 mmol), then neat HBpin (80 µL, 0.55 mmol). The reaction was monitored by NMR spectroscopy after stirring for 10 hours. The conversion is referred to internal standard HMCTS.

3.2.1. Control experiment − hydroboration of ketones without catalyst

Table S4. Reaction conditions: To a solution of C_6D_6 (500 µL) in an NMR tube with a J. Young NMR valve, was added neat ketone (ca. 0.5 mmol), then neat HBpin (80 µL, 0.55 mmol). The reaction was monitored by NMR spectroscopy after 24 hours.

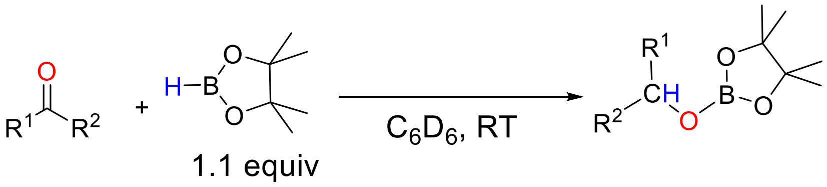

Entry	Ketone	Catalyst loading (mol %)	Time (h)	NMR conversion (%)
1	4′-Nitroacetophenone	0	24	<1
2	Acetophenone	0	24	<1
3	4′-Methoxyacetophenone	0	24	<1
4	4′-Bromoacetophenone	0	24	<1
5	4'-Chloroacetophenone	0	24	<1
6	4′-Iodoacetophenone	0	24	<1

Reagents: 0.5 mmol ketone, 0.55 mmol HBpin (80µl), reaction was performed at room temperature, ^{1}H NMR yields relative to internal standard hexamethylcyclotrisiloxane.

3.2.2. Selected NMR spectra for the catalytic hydroboration of 4′-nitroacetophenone

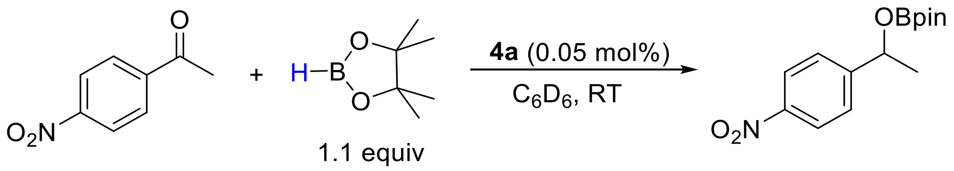

Scheme S5. Catalytic hydroboration of 4′-nitroacetophenone.

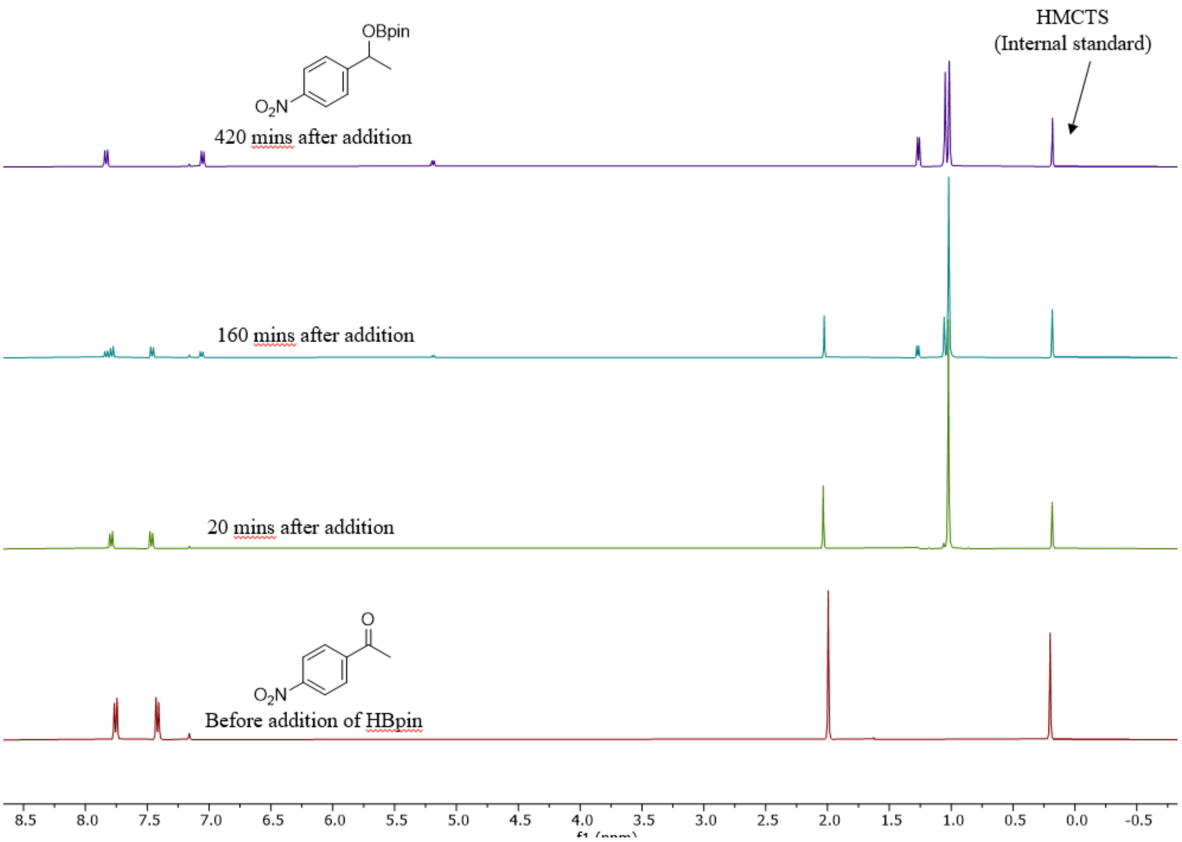

Figure S65. Monitoring the hydroboration of 4′-nitroacetophenone with HBpin in C_6D_6. Stacked ^{1}H NMR spectra of 0.5 mmol 4′-nitroacetophenone in 0.5 mL C_6D_6. The resonances at 2.00, 7.42 and 7.76 ppm correspond to the 4′-nitroacetophenone, the resonances 1.27, 5.19, 7.05 and 7.83 ppm correspond to the hydroborated product. The internal standard, hexamethylcyclotrisiloxane (HMCTS), exhibits a resonances at 0.18 ppm.

3.2.3. Concentration monitoring of hydroboration of 4′-nitroacetophenone with catalyst **4a**

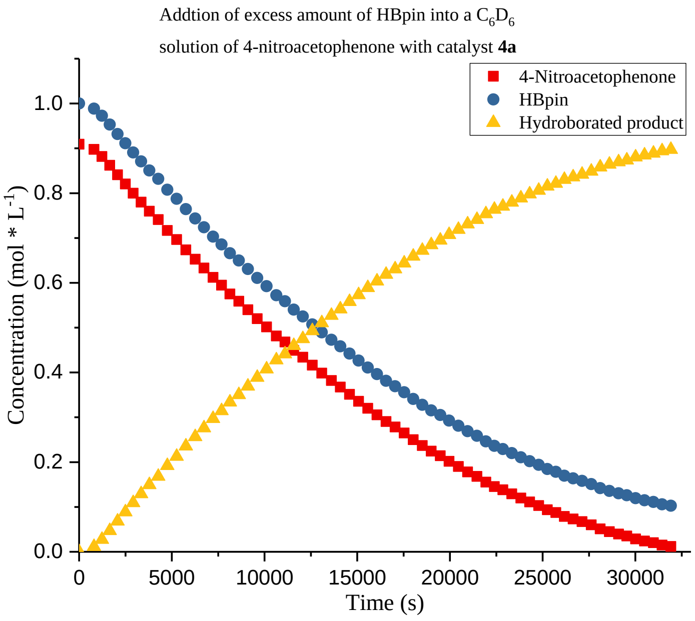

Figure S66. Concentration plot based on integration of the hydroboration of 4′-nitroaceto-phenone with HBpin in C_6D_6. To a stock solution (500 µL, with 0.00025 mmol **4a**), the neat 4′-nitroacetophenone (ca. 0.5 mmol) was added, then neat HBpin (80 µL, 0.55 mmol).

3.2.4. Concentration monitoring of hydroboration of 4′-nitroacetophenone with three different catalysts

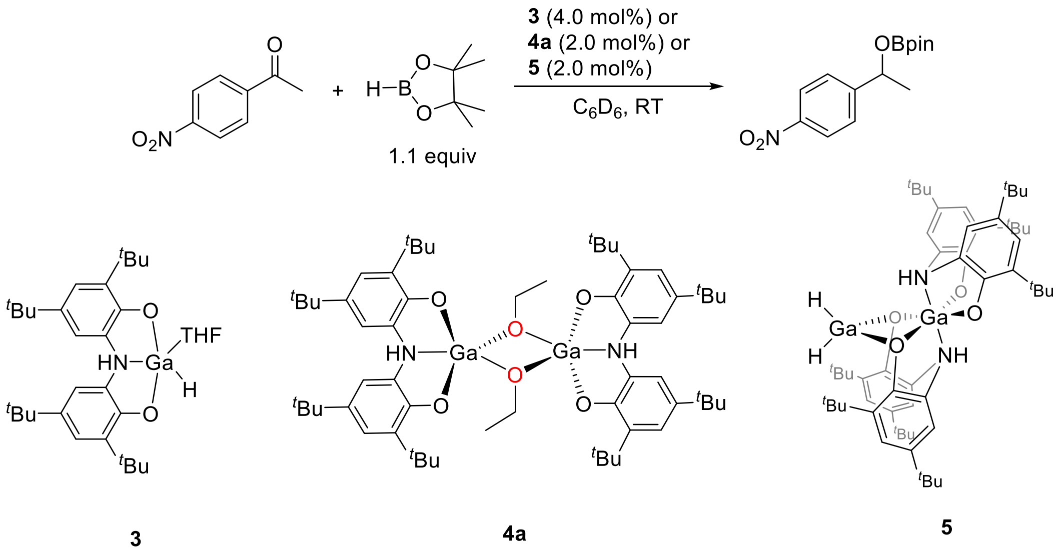

Scheme S6. Catalytic hydroboration of 4′-nitroacetophenone by three different catalysts

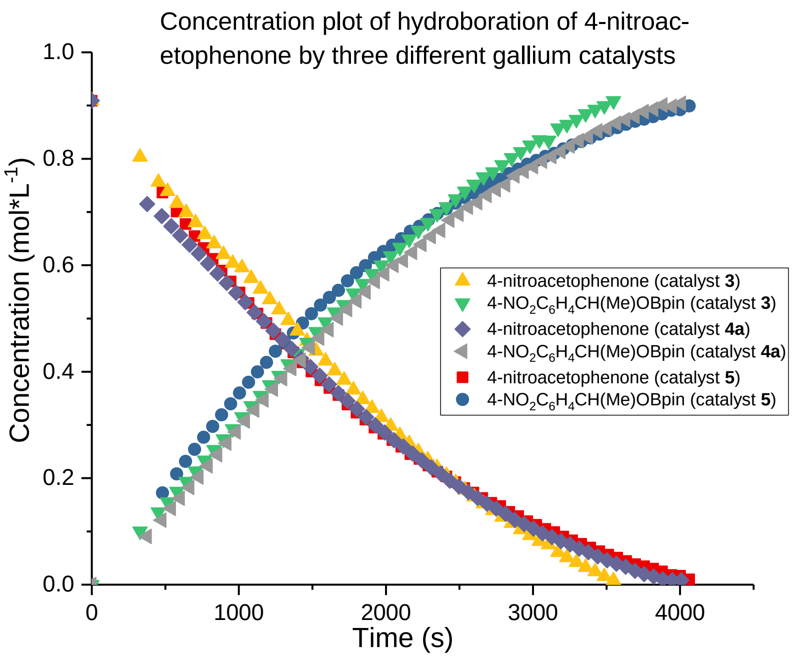

Figure S67. Concentration plot based on integration of the hydroboration of 4′-nitroaceto-phenone with HBpin in C_6D_6. C_6D_6 (500 µL) was added 0.02 mmol gallium catalyst **3** or 0.01 mmol gallium catalyst **4a** or **5** to give a colourless solution, the neat 4′-nitroacetophenone (*ca.* 0.50 mmol) was added together with neat HBpin (80 µL, 0.55 mmol). The reactions are monitored by ^{1}H NMR until the reaction completed (as all the 4′-nitroacetophenone are consumed).

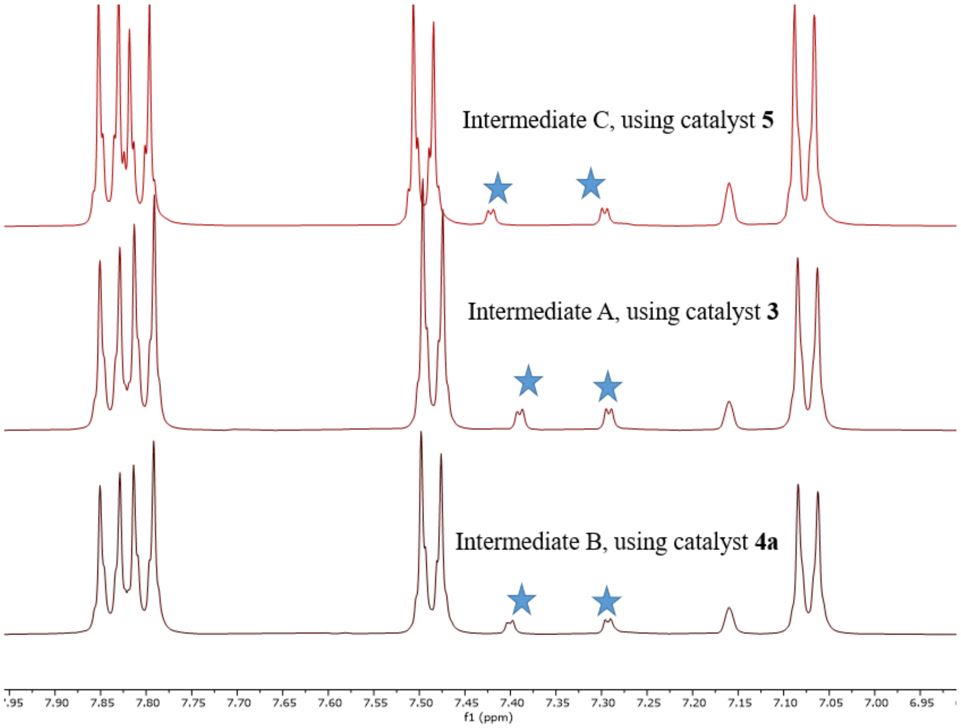

Figure S 68. Monitoring the hydroboration of 4′-nitroacetophenone with HBpin in C_6D_6. Stacked ^{1}H NMR spectra after 20 mins. The resonances at 7.48 and 7.80 ppm correspond to the 4′-nitroacetophenone, the resonances at 7.06 and 7.83 ppm correspond to the hydroborated product. The labelled resonances at approx. 7.30 and 7.40 ppm correspond to the catalyst resting state under catalytic conditions.

Table S5. ^{1}H NMR chemical shifts of gallium complexes

		^{1}H NMR shift in C_6D_6 solution					
Entry	Compound	Ga−H (ppm)	N−H (ppm)	Ar−H (ppm)		*tert*-butyl (ppm)	
1	**3**	5.65	3.92	7.35	7.15	1.59	1.35
2	**3**·BH_3py	5.67	4.01	7.35	7.20	1.59	1.35
3	**4a**	N/A	4.70	7.46	7.42	1.48	
4	**5**	5.55−5.94	4.02−5.11	6.81−7.55		1.18−1.84	
5	Intermediate A[a]	5.65	5.12	7.39	7.29	1.52	1.31
6	Intermediate B[b]	5.76	5.68	7.40	7.29	1.51	1.30
7	Intermediate C[c]	5.75	5.84	7.42	7.30	1.51	1.30
8	**7f**	N/A	3.90	7.26, 7.36	7.41, 7.86	1.56	1.31

Intermediates A, B, C were monitored by ^{1}H NMR spectroscopy through the process of hydroboration of 4′-nitroacetophenone using different catalysts. [a] Using catalyst **3**; [b] Using catalyst **4a**; [c] Using catalyst **5**.

3.2.5. Inhibition of ketone hydroboration by tetramethylethylenediamine (TMEDA)

Thomas et al. have proposed that nucleophiles can act as ‘fake’ catalysts for the hydroboration of alkenes and alkynes by reacting with HBpin to form BH_3, which acts as the catalytically active species.[5] To eliminate the possibility of the aforementioned mechanism being operative in our studies, the hydroboration of 4′-nitroacetophenone with 2.0 mol% catalyst **4a** was carried out in the presence of TMEDA. The result shows that on addition of 20 mol% of TMEDA to the reaction mixture, the conversion in the first hour decreases to 62% from 99%. We believe that this decrease in activity may be caused by the coordination of TMEDA to our catalyst.

As the addition of TMEDA prevents the possible involvement of BH_3 in the catalysis, the possibility that **4a** only acts as a nucleophile in this reaction can be ruled out.

Table S6. Gallium-catalysed hydroboration of 4′-nitroacetophenone with TMEDA [a]

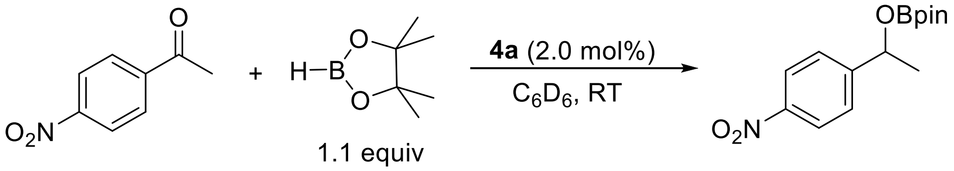

Entry	Ketone[a]	Catalyst loading (mol%)	Time (h)	NMR Conversion (%)[b]
1	4′-Nitroacetophenone	2.0	1	99
2	4′-Nitroacetophenone[c]	2.0	1	62

[a] 4′-Nitroacetophenone (0.5 mmol) and HBpin (0.55 mmol, 80 μL) in C_6D_6 (0.5 mL), HMCTS (0.05 mmol) as an internal standard, reaction was performed at room temperature, [b] ^{1}H NMR yield is calculated by integration versus HMCTS, [c] With TEMDA (0.1 mmol)

3.3. Selected data for aldehyde and ketone hydroboration products

3.3.1. $PhCH_2OBpin$: product from hydroboration of benzaldehyde.

In accordance with general procedure using benzaldehyde (53mg, 0.5 mmol) as the substrate gave the product. ^{1}H NMR (400 MHz, C_6D_6) δ (ppm) 7.41 (d, $^3J_{H–H}$ = 7.5 Hz, 2H; Ar-*H*), 7.31 – 7.22 (m, 2H; Ar-*H*), 7.21 – 7.14 (m, 1H; Ar-*H*), 5.05 (s, 2H; OCH_2), 1.16 (s, 12H; Bpin-CH_3).
^{11}B NMR (128 MHz, C_6D_6) δ (ppm) 22.76.

3.3.2. 4-Br-$C_6H_4CH_2OBpin$: product from hydroboration of 4-bromobenzaldehyde

In accordance with general procedure using 4-bromobenzaldehyde (92.5mg, 0.5 mmol) as the substrate gave the product. ^{1}H NMR (400 MHz, C_6D_6) δ (ppm) 7.27 – 7.18 (m, 2H; Ar-*H*), 6.94 (m, 2H; Ar-*H*), 4.72 (s, 2H; OCH_2), 1.03 (s, 12H; Bpin-CH_3).
^{11}B NMR (128 MHz, C_6D_6) δ (ppm) 22.66.

3.3.3. 4-MeO-$C_6H_4CH_2OBpin$: product from hydroboration of 4-methoxybenzaldehyde

^{1}H NMR (400 MHz, C_6D_6) δ (ppm) 7.23 (d, $^3J_{H–H}$ = 6.5 Hz, 2H; Ar-*H*), 6.82 – 6.60 (m, 2H; Ar-*H*), 4.90 (s, 2H; OCH_2), 3.30 (s, 3H; OCH_3), 1.04 (s, 12H; Bpin-CH_3).
^{11}B NMR (128 MHz, C_6D_6) δ (ppm) 22.77.

3.3.4. 2,4,6-$Me_3C_6H_2CH_2OBpin$: product from hydroboration of mesitaldehyde

^{1}H NMR (400 MHz, C_6D_6) δ (ppm) 6.71 (s, 2H; Ar*H*), 5.00 (s, 2H; OCH_2), 2.35 (s, 6H; $C_6H_2(CH_3)_2$), 2.11 (s, 3H; $C_6H_2CH_3$), 1.04 (s, 12H; Bpin-CH_3).
^{11}B NMR (128 MHz, C_6D_6) δ (ppm) 22.58.

3.3.5. 4-FC_6H_4-CH_2OBpin: product from hydroboration of 4-fluorobenzaldehyde

^{1}H NMR (400 MHz, C_6D_6) δ (ppm) 7.14 – 7.00 (m, 2H; Ar-*H*), 6.84 – 6.70 (m, 2H; Ar-*H*), 4.78 (s, 2H; OCH_2), 1.04 (s, 12H; Bpin-CH_3).

^{11}B NMR (128 MHz, C_6D_6) δ (ppm) 22.69.

3.3.6. 4-Me-$C_6H_4CH_2$OBpin: product from hydroboration of 4-methylbenzaldehyde

^{1}H NMR (500 MHz, C_6D_6) δ (ppm) 7.25 (d, $^{3}J_{H–H}$ = 7.9 Hz; Ar-*H*), 6.96 (d, $^{3}J_{H–H}$ = 7.8 Hz, 2H; Ar-*H*), 4.94 (s, 2H; OCH_2), 2.09 (s, 3H; C_6H_4CH_3), 1.05 (s, 12H; Bpin-CH_3).

^{11}B NMR (128 MHz, C_6D_6) δ (ppm) 22.76.

3.3.7. (4-Br-C_6H_4)(CH_3)C(H)OBpin: product from hydroboration of 4'-bromoacetophenone

^{1}H NMR (400 MHz, C_6D_6) δ (ppm) 7.47 – 7.16 (m, 2H; Ar-*H*), 7.06 – 6.90 (m, 2H; Ar-*H*), 5.20 (q, $^{3}J_{H–H}$ = 6.5 Hz, 1H; OC*H*), 1.32 (d, $^{3}J_{H–H}$ = 6.5 Hz, 3H; CH_3), 1.06 – 0.99 (s, 12H; Bpin-CH_3).

^{11}B NMR (128 MHz, C_6D_6) δ (ppm) 22.41.

3.3.8. (4-MeO-C_6H_4)(CH_3)C(H)OBpin: product from hydroboration of 4'-methoxy-acetophenone

^{1}H NMR (400 MHz, C_6D_6) δ (ppm) 7.50 – 7.20 (m, 2H; Ar-*H*), 6.89 – 6.60 (m, 2H; Ar-*H*), 5.37 (q, $^{3}J_{H–H}$ = 6.5 Hz, 1H; OC*H*), 3.32 (s, 3H; OCH_3), 1.46 (d, $^{3}J_{H–H}$ = 6.5 Hz, 3H; CHCH_3), 1.03 (s, 12H; Bpin-CH_3),

^{11}B NMR (128 MHz, C_6D_6) δ (ppm) 22.73.

3.3.9. $(CH_3)_2$C(H)OBpin: product from hydroboration of acetone

NMR data are identical to those previously reported.

^{1}H NMR (400 MHz, C_6D_6) δ (ppm) 4.43 (hept, $^{3}J_{H–H}$ = 6.2 Hz, 1H; OC*H*), 1.15 (d, $^{3}J_{H–H}$ = 6.1 Hz, 6H; CH(CH_3)$_2$), 1.06 (s, 12H; Bpin-CH_3).

^{11}B NMR (128 MHz, C_6D_6) δ (ppm) 22.46.

3.3.10. $(CH_3)_2C(H)CH_2OBpin$: product from hydroboration of isobutyraldehyde

In accordance with general procedure using isobutyraldehyde (36mg, 0.5 mmol) as the substrate gave product.

^{1}H NMR (400 MHz, C_6D_6) δ (ppm) 3.69 (d, $^3J_{H–H}$ = 6.5 Hz, 2H; OCH_2), 1.96 – 1.61 (m, 1H; CH), 1.06 (s, 12H; Bpin-CH_3), 0.83 (d, $^3J_{H–H}$ = 6.7 Hz, 6H; CH_3).

^{11}B NMR (128 MHz, C_6D_6) δ (ppm) 22.49.

3.3.11. (4-NO_2-C_6H_4)C(H)(CH_3)OBpin: product from hydroboration of 4'-nitroacetophenone

^{1}H NMR (400 MHz, C_6D_6) δ (ppm) 8.12 – 7.58 (m, 2H; Ar-H), 7.07 – 7.01 (m, 2H; Ar-H), 5.19 (q, $^3J_{H–H}$ = 6.5 Hz, 1H; OCH), 1.27 (d, $^3J_{H–H}$ = 6.5 Hz, 3H; CHCH_3), 1.03 (s, 12H; Bpin-CH_3).

^{11}B NMR (128 MHz, C_6D_6) δ (ppm) 22.40.

3.3.12. (4-I-C_6H_4)C(H)(CH_3)OBpin: product from hydroboration of 4'-iodoacetophenone

^{1}H NMR (400 MHz, C_6D_6) δ (ppm) 7.55 – 7.35 (m, 2H; Ar-H), 7.01 – 6.73 (m, 2H; Ar-H), 5.18 (q, $^3J_{H–H}$ = 6.5 Hz, 1H; OCH), 1.32 (d, $^3J_{H–H}$ = 6.5 Hz, 3H; CHCH_3), 1.04 – 0.95 (s, 12H; Bpin-CH_3).

^{11}B NMR (128 MHz, C_6D_6) δ (ppm) 22.47.

3.3.13. (4-Cl-C_6H_4)C(H)(CH_3)OBpin: product from hydroboration of 4'-chloroacetophenone

^{1}H NMR (400 MHz, C_6D_6) δ (ppm) 7.07 (m, 4H; Ar-H)), 5.22 (q, $^3J_{H–H}$ = 6.5 Hz, 1H; OCH), 1.33 (d, $^3J_{H–H}$ = 6.5 Hz, 3H; CHCH_3), 1.04 – 0.96 (s, 12H, CH_3; Bpin-CH_3).

^{11}B NMR (128 MHz, C_6D_6) δ (ppm) 22.42.

3.4. Kinetic studies of hydroboration of aldehydes and ketones

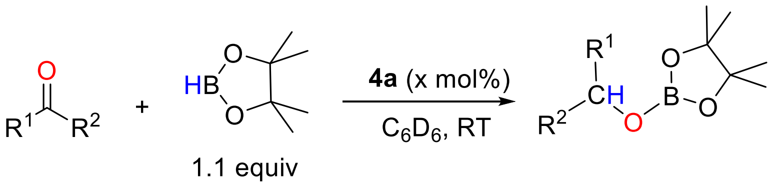

Scheme S7. Catalytic hydroboration of ketones with pre-catalyst **4a**

The kinetic studies were conducted as described by Burés.[6]

An expression could be used to represent the kinetic order of this reaction:

$$[P] = k_{obs} \int [Ketone]^{\alpha} \times [HBpin]^{\beta} \times [Ga]^{\gamma} dt$$

The reaction order can be obtained via a graphical representation.

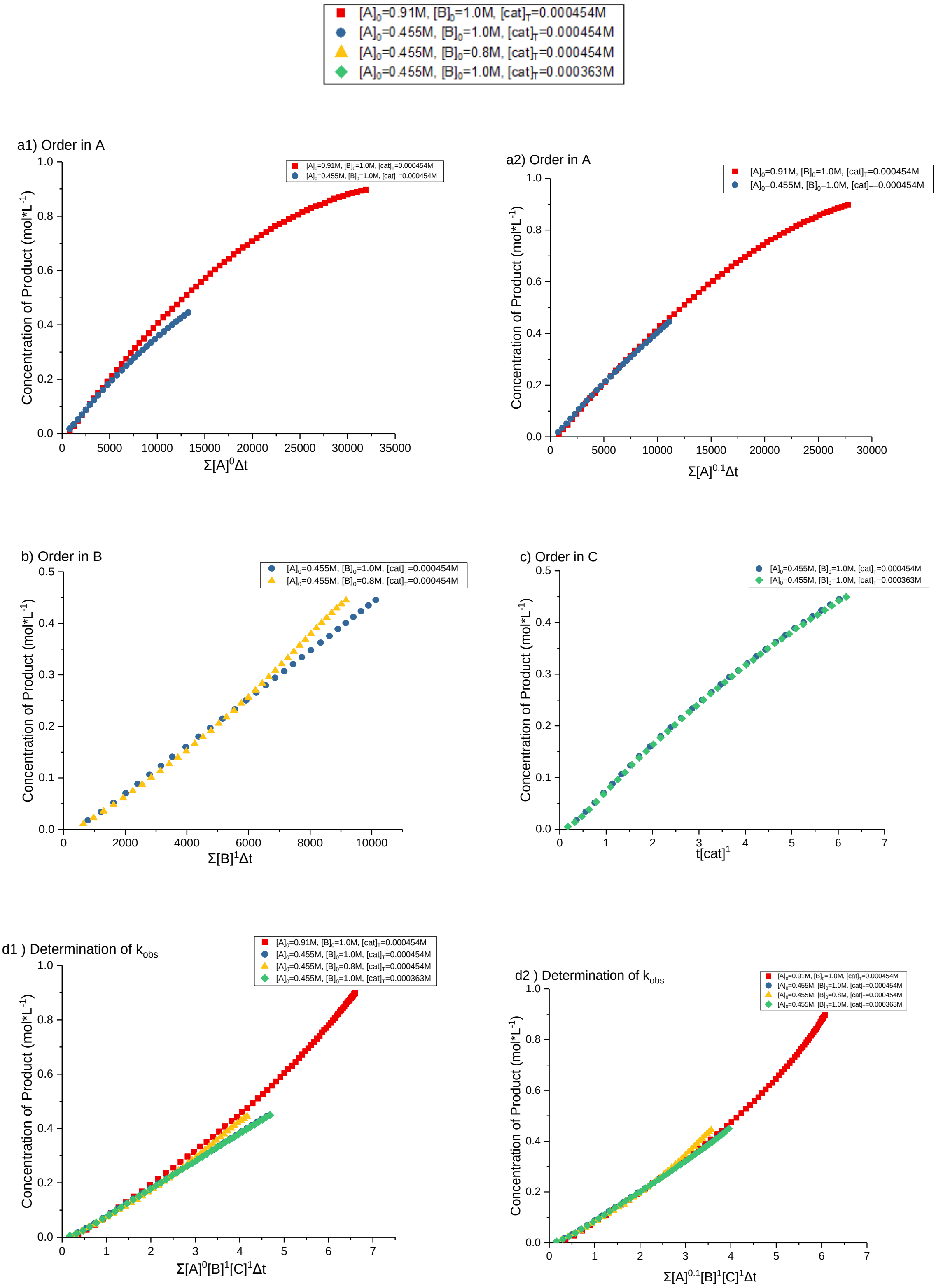

Figure S69. Variable time normalization analysis of the kinetic profiles, a1, b, c and d1 are the orders obtained from the analysis, while a2 and d2 are visual representations of the real values. [A] = concentration of 4′-nitroacetophenone, [B] = concentration of pinacolborane, $[cat]_T$ = concentration of pre-catalyst **4a**. Concentration were recorded via the NMR integration of the

product based on an internal standard. For accurate integration, relaxation delay has been set has 35s.

Herein, variable time normalization analysis method has been applied to determine the order of this reaction, by this method, the kinetic concentration effect of each product has been removed. The Figure S69 corresponds to a reaction with pseudo zero order in concentration of ketones, first order in catalyst and pinacol borane (first set). An optimised fitting can be obtained by adjusting the order of ketones to 0.1 (second set).

$$\text{First set of reaction order } [P] = k_{obs1} \int [Ketone]^{0} \times [HBpin]^{1} \times [Ga]^{1} dt$$

$$\text{Second set of reaction order } [P] = k_{obs2} \int [Ketone]^{0.1} \times [HBpin]^{1} \times [Ga]^{1} dt$$

Based on the kinetic studies, the reaction of **6** or **7** with HBpin is the rate determining step while the insertion of ketone into the gallium hydride of **5** to form **6** or **7** is a fast step.

3.5. A simplified proposed mechanism for hydroboration of aldehydes and ketones

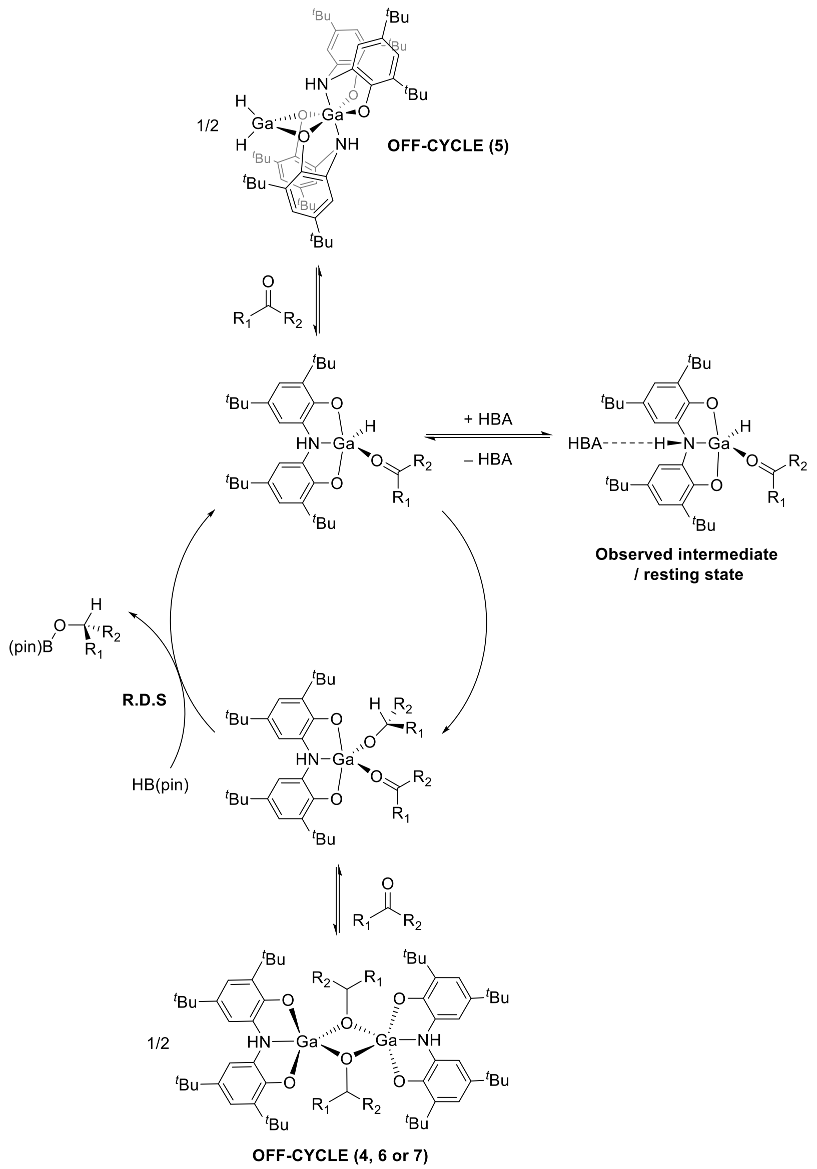

Figure S70. Proposed mechanism for hydroboration of aldehydes and ketones

3.6. Simulation studies of hydroboration of ketones

Using the experimental data from Figure S67 (using catalyst **3**), a simulation model for the proposed mechanism of ketone hydroboration was developed by COPASI with the Hooke & Jeeves' method.[7]

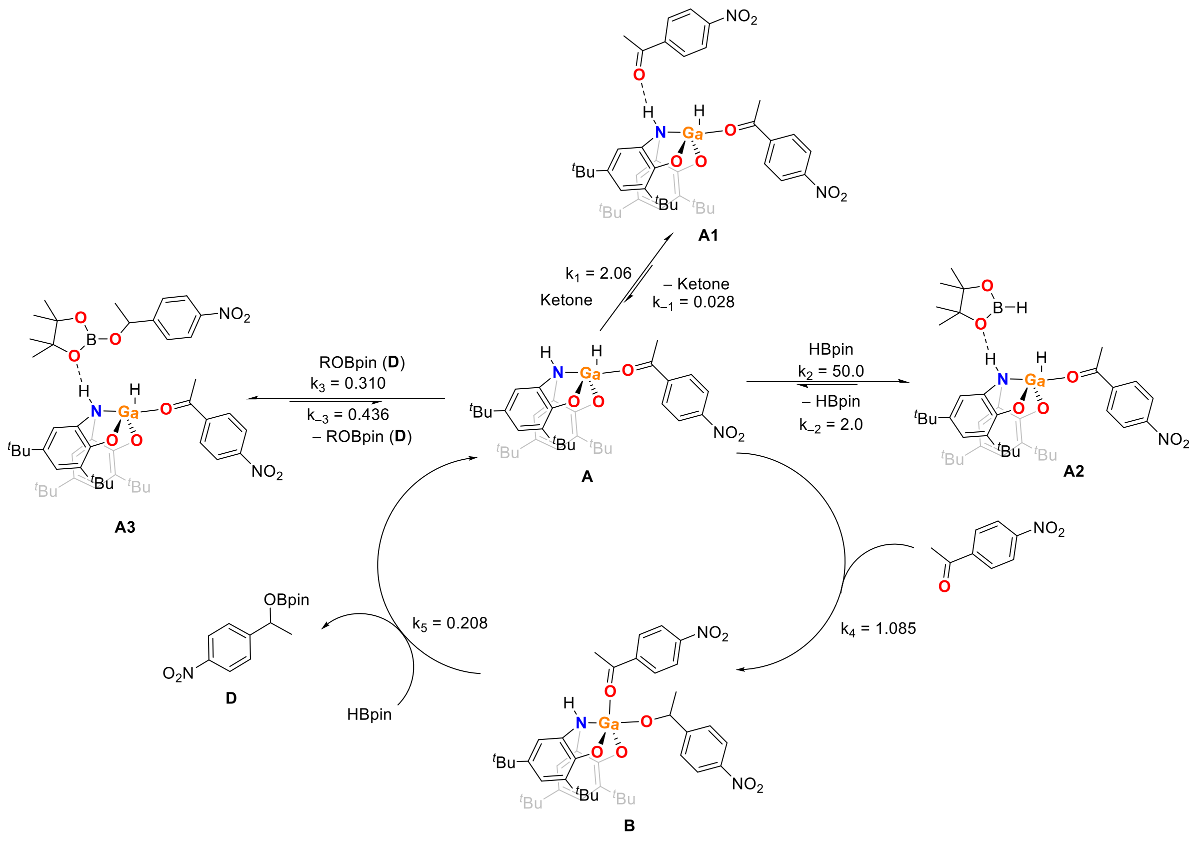

Figure S71. Simulation studies for hydroboration of ketone (4′-nitroacetophenone) using COPASI.

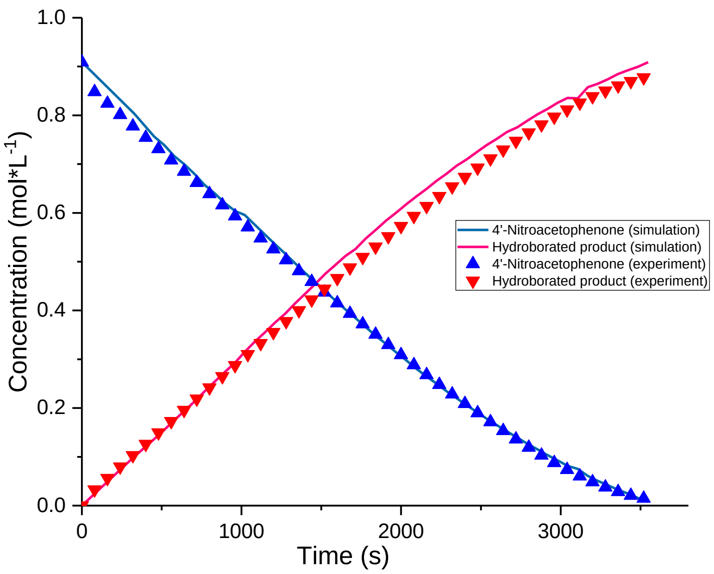

Figure S72. Fitting of the concentration plots for hydroboration of 4′-nitroacetophenone based on experimental results and simulation by COPASI.

We propose a mechanism with a gallium monohydride **A** acting as the catalyst. Complex **A** reacts with the ketone (4′-nitroaceotophenone) through an insertion step (with carbonyl insertion into gallium−hydride bond). The reaction of **A** with ketone results in the formation of gallium alkoxide **B**, which can react with HBpin to give a borate ester and regenerate the gallium monohydride **A**.

The amine (N−H) ligand backbone of complex **A** can potentially form hydrogen bonding with the oxygen atoms on HBpin or the ketone. **A** is in solution equilibria with **A1** (in the presence of ketone), **A2** (in the presence of HBpin) and **A3** (in the presence of borate ester).

The simulation studies suggests that the reaction of HBpin with **B** is the rate determining step in the catalytic cycle, with a reaction rate constant k_5 as 0.208 ($mL \cdot mmol^{-1} \cdot s^{-1}$), while the carbonyl insertion step has a rate constant k_4 as 1.085 ($mL \cdot mmol^{-1} \cdot s^{-1}$), which matches our finding of the observed rate law as $k[HBpin]^1[ketone]^0[catalyst]^1$ based on experiments.

4. Catalytic hydroboration of carbon dioxide

General procedure for preparing stock solutions for the hydroboration of carbon dioxide: To a dried ampoule containing the pre-catalyst **4a** (40.0 mg) and hexamethylcyclotrisiloxane (0.444 g) as an internal standard, was added 20 mL of C_6D_6. This gives rise to a stock solution containing 0.0019 $mol \cdot L^{-1}$ of catalyst **4a** and 0.1 $mol \cdot L^{-1}$ of hexamethylcyclotrisiloxane.

To the stock solution (500 μL, with 0.00093 mmol **4a**), neat HBpin (13.5 μL, 0.093 mmol) was added using a gastight syringe, then degassed via freeze-pump-thaw method for three times prior to exposure to ~2.0 bar pressure carbon dioxide at room temperature. The ampoule was sealed and kept stirring at room temperature, and NMR data were collected twice after 20 and 38 hours.

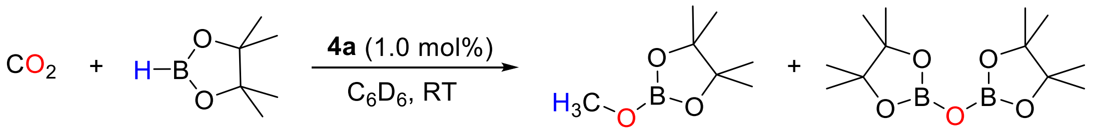

Scheme S8. Catalytic hydroboration of carbon dioxide.

4.1. NMR spectra for catalytic hydroboration of carbon dioxide

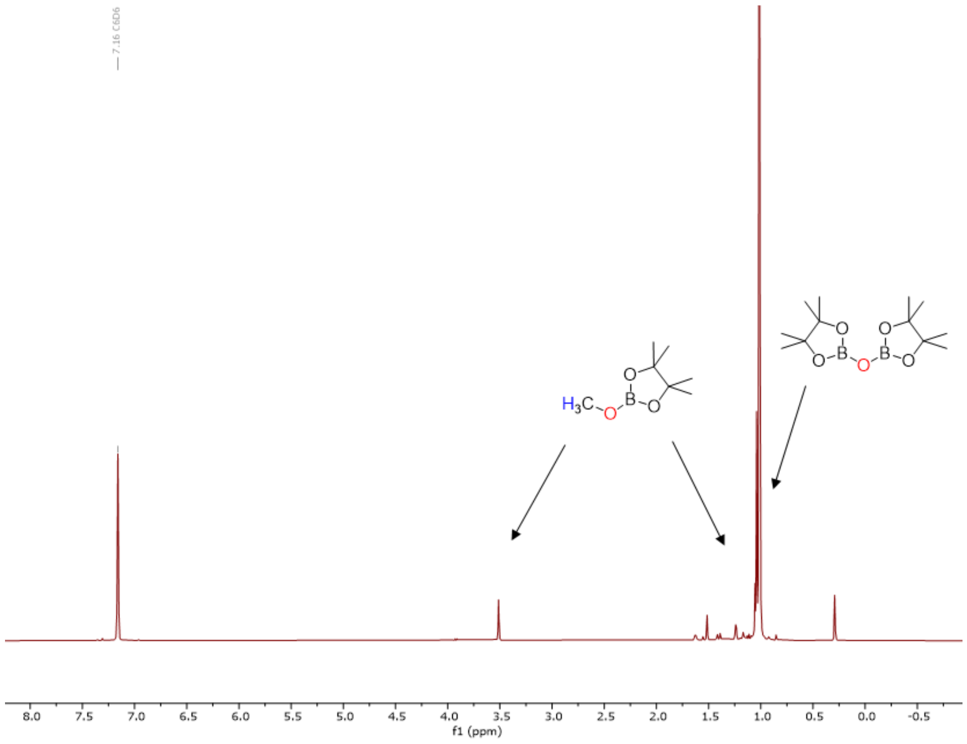

Figure S73. ^{1}H NMR spectrum of the hydroboration product of carbon dioxide. Singlet resonance at δ 1.04 ppm and δ 3.50 ppm correspond to CH_3OBpin, δ 1.01 ppm corresponds to $O(Bpin)_2$

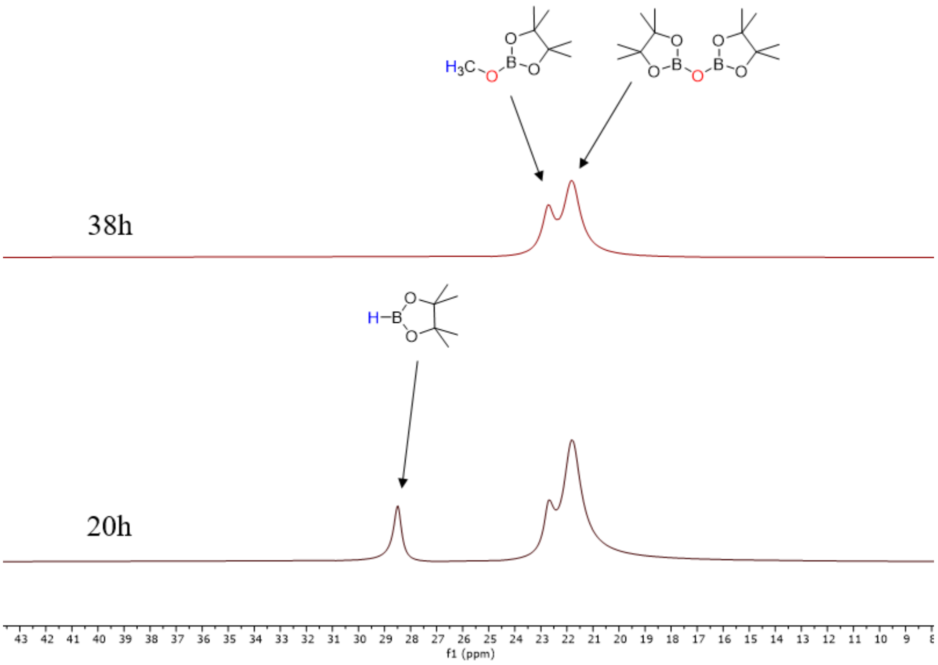

Figure S74. Monitoring the hydroboration of carbon dioxide with HBpin in C_6D_6. Stacked $^{11}B\{H\}$ NMR spectra of a 0.093 mmol HBpin in a stock solution of C_6D_6 (0.5mL) with 0.00093 mmol **4a** under 2 bar CO_2 at room temperature. Signal at δ 22.67 ppm corresponds to CH_3OBpin, δ 21.76 ppm corresponds to $O(Bpin)_2$.

5. X-ray crystallographic studies

Single-crystal X-ray diffraction data were collected using an Oxford Diffraction Supernova dual-source diffractometer equipped with a 135 mm Atlas CCD area detector. Crystals were selected under Paratone-N oil, mounted on micromount loops and quench-cooled using an Oxford Cryosystems open flow N_2 cooling device. Data were collected at 150 K using mirror monochromated Cu K_α radiation (λ = 1.5418 Å; Oxford Diffraction Supernova) and processed using the CrysAlisPro package, including unit cell parameter refinement and inter-frame scaling (which was carried out using SCALE3 ABSPACK within CrysAlisPro).[8] Structures were subsequently solved using direct methods and refined on F^2 using the SHELXL package.[9]

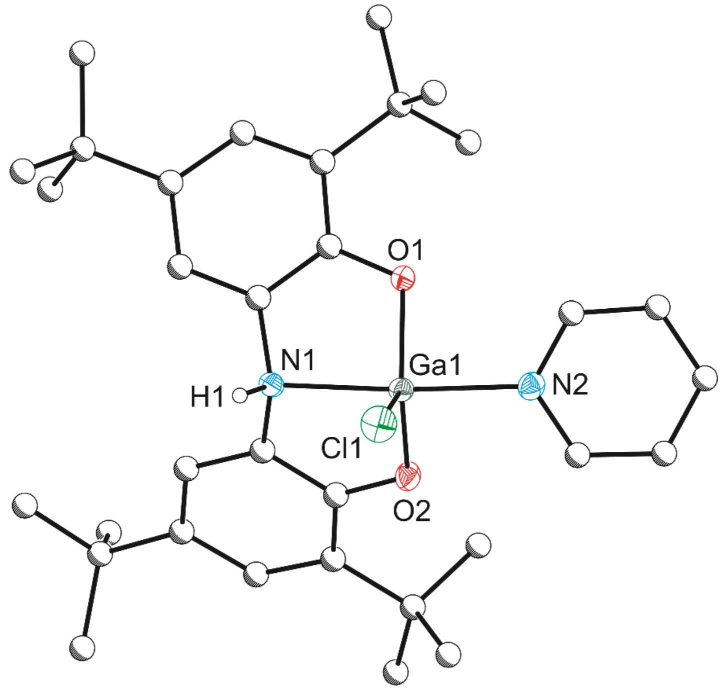

Figure S 75. Molecular structure of **1**. Anisotropic displacement ellipsoids set at 50% probability. Hydrogen atoms (with the exception of H1) have been omitted for clarity. All carbon atoms are pictured as spheres of arbitrary radius. Selected interatomic distances [Å] and angles [°]: Ga1–O1 1.8456(9), Ga1–O2 1.8461(9), Ga1–N1 2.1418(11), Ga1–N2 2.0803(11), Ga1–Cl 2.2073(4); O1-Ga1-O2 122.34(4), O1-Ga1-N2 88.59(4), O2-Ga1-N2 90.15(4), O1-Ga1-N1 84.72(4), O2-Ga1-N1 85.10(4), N2-Ga1-N1 168.03(4), O1-Ga1-Cl1 120.25(3), O2-Ga1-Cl1 117.16(3), N2-Ga1-Cl1 96.35(3), N1-Ga1-Cl1 95.59(3). Symmetry operation ′: 1–*x*, 2–*y* –*z*.

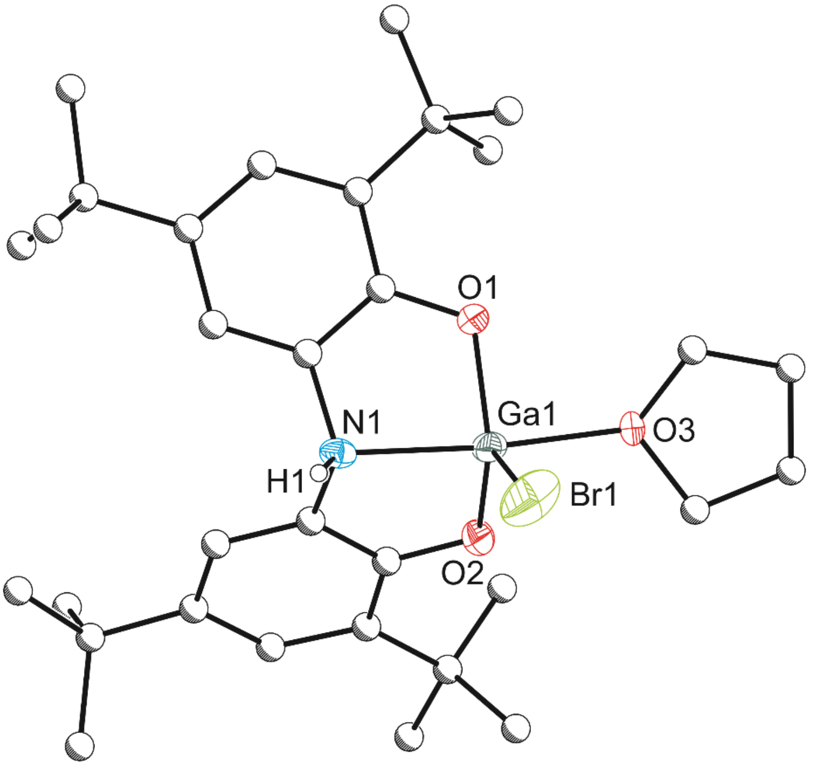

Figure S 76. Molecular structure of **2**. Anisotropic displacement ellipsoids set at 50% probability. Hydrogen atoms (with the exception of H1) have been omitted for clarity. All carbon atoms are pictured as spheres of arbitrary radius. Selected interatomic distances [Å] and angles [°]: Ga1–O1 1.837(2), Ga1–O2 1.833(2), Ga1–N1 2.120(3), Ga1–O3 2.153(7), Ga1–Br1 2.3319(6); O2-Ga1-O1 120.81(10), O2-Ga1-N1 86.26(10), O1-Ga1-N1 85.87(10), O2-Ga1-O3 84.51(19), O1-Ga1-O3 85.72(19), N1-Ga1-O3 162.1(2), O2-Ga1-Br1 118.34(7), O1-Ga1-Br1 120.82(8), N1-Ga1-Br1 99.63(7), O3-Ga1-Br1 98.3(2)

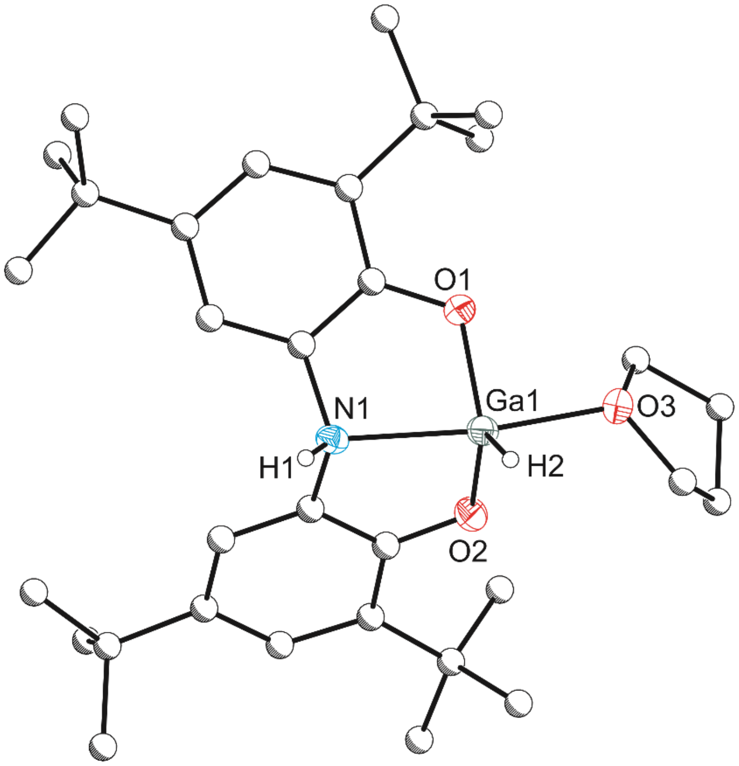

Figure S 77. Molecular structure of **3**. Anisotropic displacement ellipsoids set at 50% probability. Hydrogen atoms (with the exception of H1 and H2) have been omitted for clarity. All carbon atoms are pictured as spheres of arbitrary radius. Selected interatomic distances [Å] and angles [°]: Ga1–O1 1.857(2), Ga1–O2 1.865(2), Ga1–O3 2.111(2), Ga1–N1 2.191(2); O1-Ga1-O2 113.54(9), O1-Ga1-O3 85.54(9), O2-Ga1-O3 87.35(9), O1-Ga1-N1 83.10(9), O2-Ga1-N1 83.35(9), O3-Ga1-N1 161.03(9).

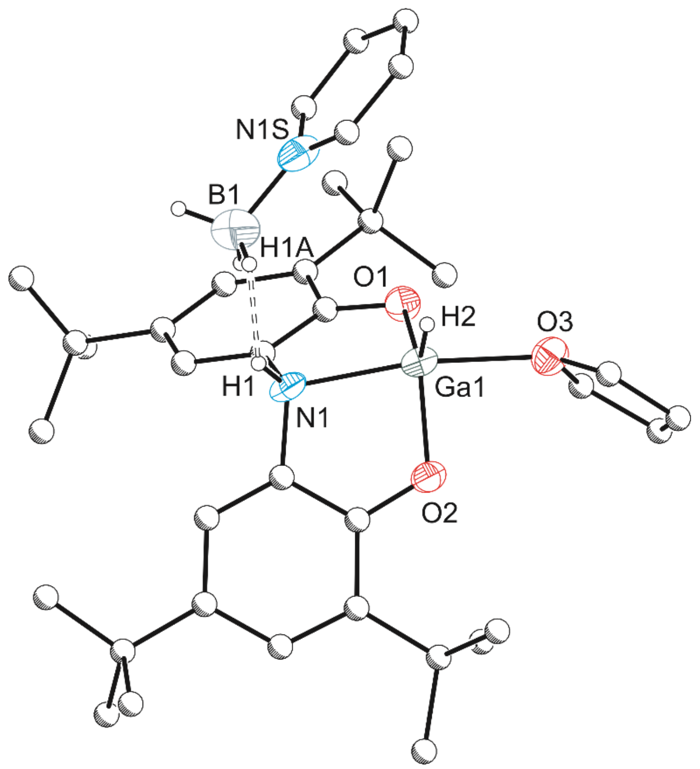

Figure S78. Molecular structure of **3**·1.5(BH_3:py). Anisotropic displacement ellipsoids set at 50% probability. Hydrogen atoms (with the exception of H1) have been omitted for clarity. All carbon atoms are pictured as spheres of arbitrary radius. Selected interatomic distances [Å] and angles [°]: Ga1–O1 1.853(2), Ga1–O2 1.870(2), Ga1–O3 2.145(2), Ga1–N1 2.151(3); O1-Ga1-O2 121.24(10), O1-Ga1-N1 85.05(8), O1-Ga1-O2 113.37(10), O1-Ga1-O3 86.30(10), O2-Ga1-O3 86.37(9), O1-Ga1-N1 84.59(10), O2-Ga1-N1 83.27(9), O3-Ga1-N1 162.21(9).

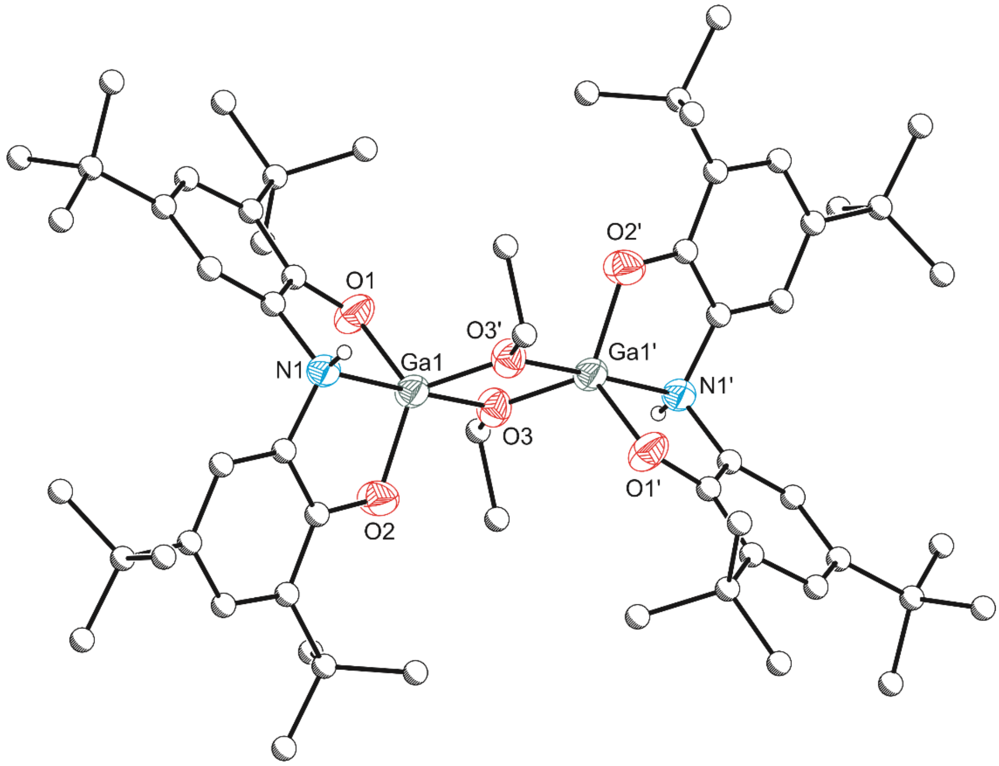

Figure S 79. Molecular structure of **4a**. Anisotropic displacement ellipsoids set at 50% probability. Hydrogen atoms (with the exception of H1) have been omitted for clarity. All carbon atoms are pictured as spheres of arbitrary radius. Selected interatomic distances [Å] and angles [°]: Ga1–O1 1.838(2), Ga1–O2 1.836(2), Ga1–N1 2.149(2), Ga1–O3 1.960(2), Ga1–O3′ 1.878(2); O1-Ga1-O2 121.24(10), O1-Ga1-N1 85.05(8), O1-Ga1-O3 94.43(8), O1-Ga1-O3′ 117.99(9), O2-Ga1-N1 85.20(8), O2-Ga1-O3 96.15(8), O2-Ga1-O3′ 120.76(9), N1-Ga1-O3 178.63(7), N1-Ga1-O3′ 100.89(8), O3-Ga1-O3′ 78.22(8). Symmetry operation ′: 1–*x*, 2–*y* –*z*.

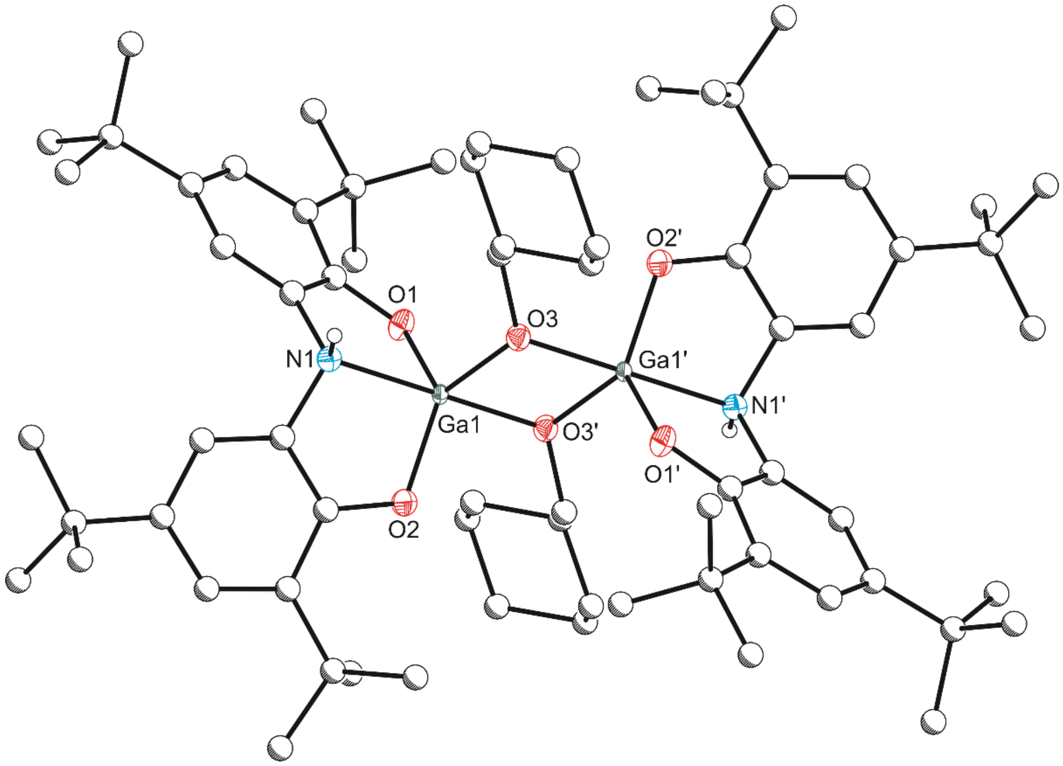

Figure S 80. Molecular structure of **4b**. Anisotropic displacement ellipsoids set at 50% probability. Hydrogen atoms (with the exception of H1) have been omitted for clarity. All carbon atoms are pictured as spheres of arbitrary radius. Selected interatomic distances [Å] and angles [°]: Ga1–O1 1.8417(14), Ga1–O2 1.8466(14), Ga1–O3 1.9006(13), Ga1–O3′ 1.9612(13), Ga1–N1 2.1366(16); O1-Ga1-O2 125.64(6), O1-Ga1-O3 110.83(6), O2-Ga1-O3 123.40(6), O1-Ga1-O3′ 98.24(6), O2-Ga1-O3′ 94.69(6), O3-Ga1-O3′ 79.69(6), O1-Ga1-N1 85.30(6), O2-Ga1-N1 84.65(6), O3-Ga1-N1 97.38(6), O3′ -Ga1-N1 176.02(6), O1-Ga1-Ga1′ 108.83(4), O2-Ga1-Ga1′ 114.01(4), O3-Ga1-Ga1′ 40.60(4), O3′ -Ga1-Ga1′ 39.09(4), N1-Ga1-Ga1′ 137.92(5). Symmetry operation ′: 1–*x*, 2–*y* –*z*.

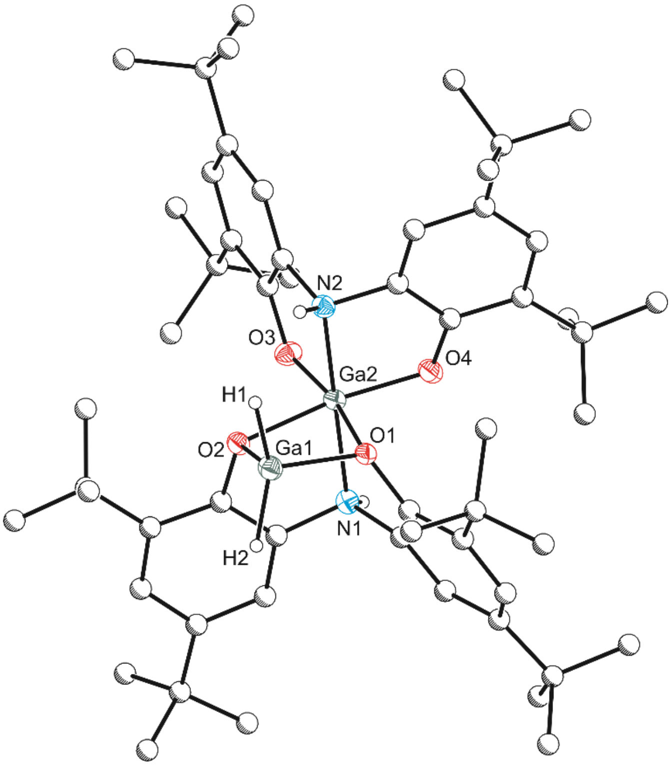

Figure S 81. Molecular structure of **5**. Anisotropic displacement ellipsoids set at 50% probability. Hydrogen atoms (with the exception of H1, H2) have been omitted for clarity. All carbon atoms are pictured as spheres of arbitrary radius. Selected interatomic distances [Å] and angles [°]: Ga1–O1 1.9278(19), Ga1–O2 1.951(2), Ga2–O3 1.872(2), Ga2–O4 1.883(2), Ga2–N1 2.045(2), Ga2–N2 2.053(2), Ga2–O2 2.0852(19), Ga2–O1 2.1311(19); O1-Ga1-O2 84.88(8), O3-Ga2-O4 101.22(9), O3-Ga2-N1 102.18(9), O4-Ga2-N1 95.63(9), O3-Ga2-N2 87.97(9), O4-Ga2-N2 86.35(9), N1-Ga2-N2 169.02(10), O3-Ga2-O2 92.02(8), O4-Ga2-O2 166.76(8), N1-Ga2-O2 81.71(9), N2-Ga2-O2 93.89(9), O3-Ga2-O1 168.18(8), O4-Ga2-O1 90.04(8), N1-Ga2-O1 80.06(9), N2-Ga2-O1 89.16(8), O2-Ga2-O1 76.73(7).

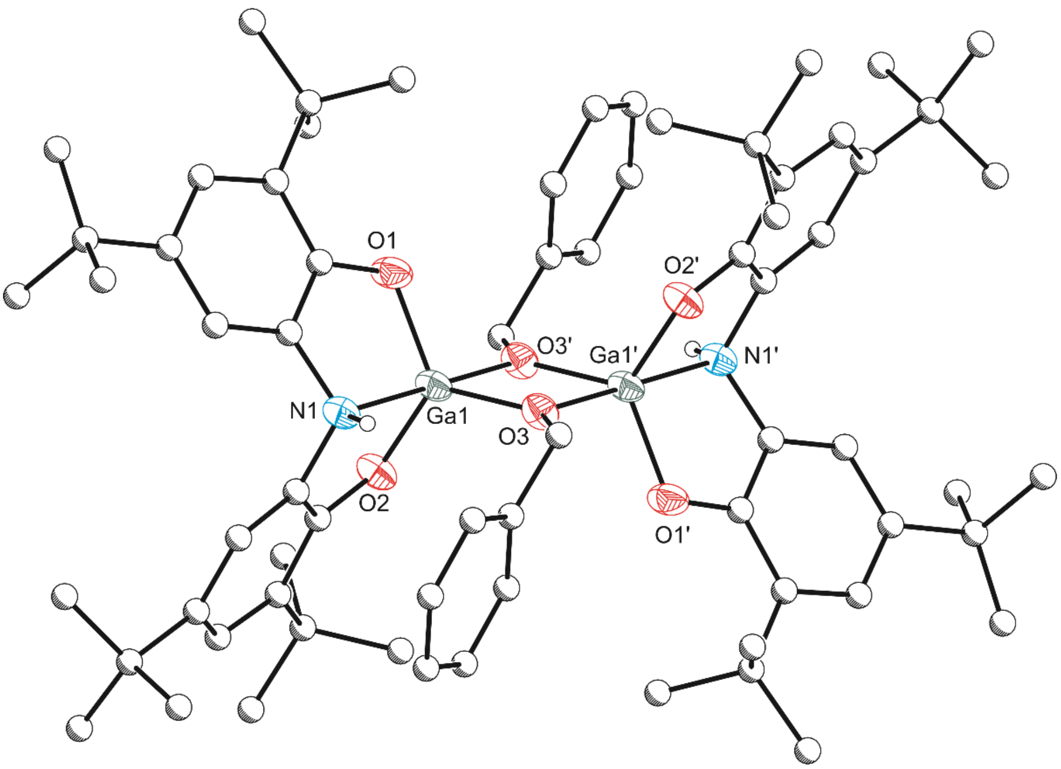

Figure S 82. Molecular structure of **6a**. Anisotropic displacement ellipsoids set at 50% probability. Hydrogen atoms (with the exception of H1) have been omitted for clarity. All carbon atoms are pictured as spheres of arbitrary radius. Selected interatomic distances [Å] and angles [°]: Ga1–O1 1.842(2), Ga1–O2 1.835(2), Ga1–O3 1.874(2), Ga1–O3′ 1.959(2), Ga1–N1 2.132(3); O2-Ga1-O1 121.63(10), O2-Ga1-O3 119.38(9), O1-Ga1-O3 118.96(10), O2-Ga1-O3′ 95.51(9), O1-Ga1-O3′ 94.60(9), O3-Ga1-O3′ 77.70(9), O2-Ga1-N1 84.90(9), O1-Ga1-N1 85.50(10), O3-Ga1-N1 101.77(9), O3′ -Ga1-N1 179.44(10).

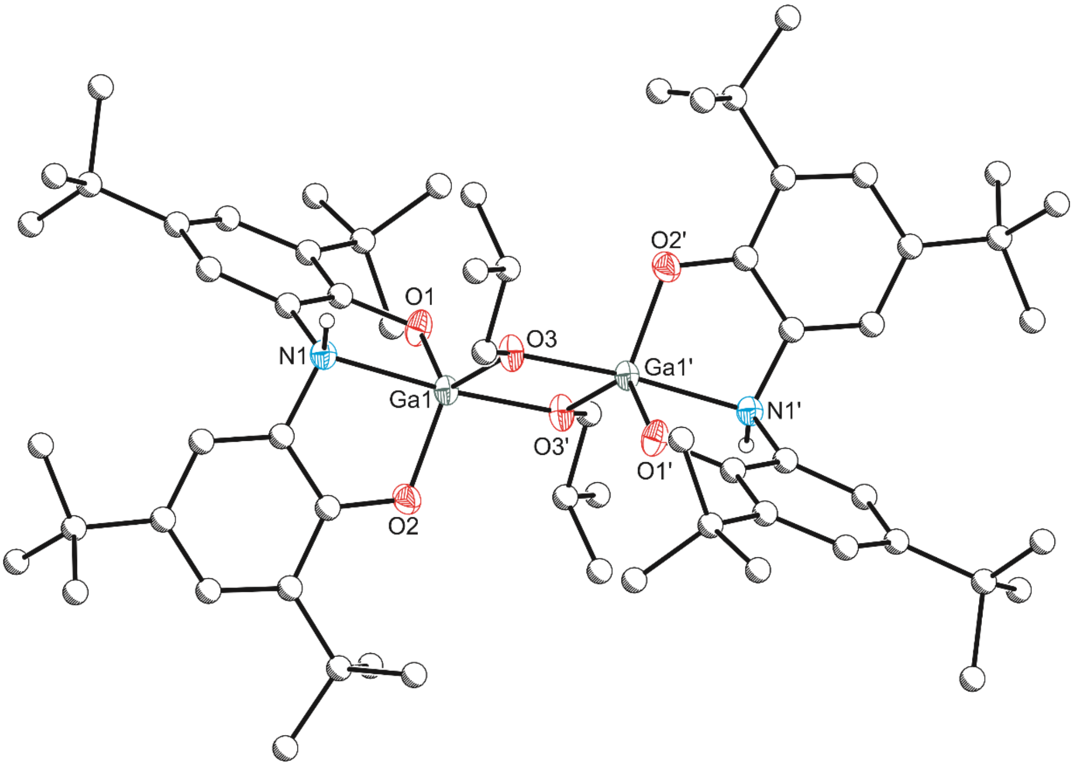

Figure S 83. Molecular structure of **6b**. Anisotropic displacement ellipsoids set at 50% probability. Hydrogen atoms (with the exception of H1) have been omitted for clarity. All carbon atoms are pictured as spheres of arbitrary radius. Selected interatomic distances [Å] and angles [°]: Ga1–O1 1.8445(19), Ga1–O2 1.8396(19), Ga1–O3 1.8847(18), Ga1–O3′ 1.9634(18), Ga1–N1 2.152(2); O2-Ga1-O1 117.84(9), O2-Ga1-O3 115.96(9), O1-Ga1-O3 126.20(9), O2-Ga1-O3′ 99.50(8), O1-Ga1-O3′ 93.88(8), O3-Ga1-O3′ 77.68(8), O2-Ga1-N1 85.33(8), O1-Ga1-N1 84.96(8), O3-Ga1-N1 99.06(8), O3′-Ga1-N1 174.99(8), Ga1-O3-Ga1′ 102.32(8).

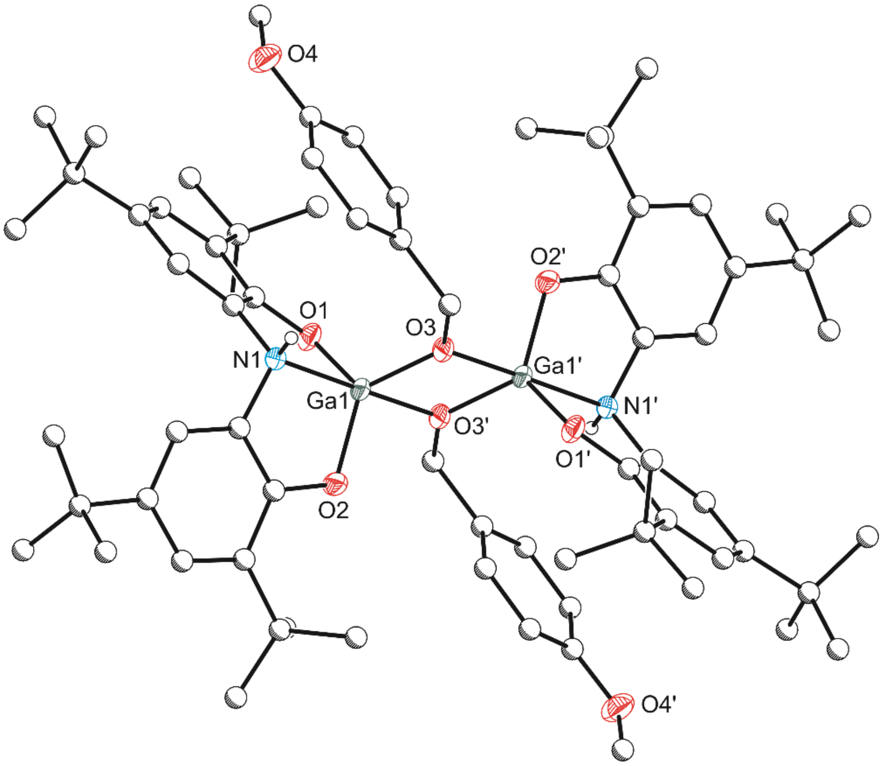

Figure S 84. Molecular structure of **6c**. Anisotropic displacement ellipsoids set at 50% probability. Hydrogen atoms (with the exception of H1) have been omitted for clarity. All carbon atoms are pictured as spheres of arbitrary radius. Selected interatomic distances [Å] and angles [°]: Ga1–O1 1.8397(10), Ga1–O2 1.8459(10), Ga1–O3 1.8813(10), Ga1–O3′ 1.9666(9), Ga1–N1 2.1308(11); O1-Ga1-O2 121.16(5), O1-Ga1-O3 120.50(4), O2-Ga1-O3 118.27(4), O1-Ga1-O3′ 94.77(4), O2-Ga1-O3′ 95.08(4), O3-Ga1-O3′ 77.31(4), O1-Ga1-N1 85.10(4), O2-Ga1-N1 85.59(4), O3-Ga1-N1 102.14(4), O3′-Ga1-N1 179.28(4), Ga1-O3-Ga1′ 102.69(4).

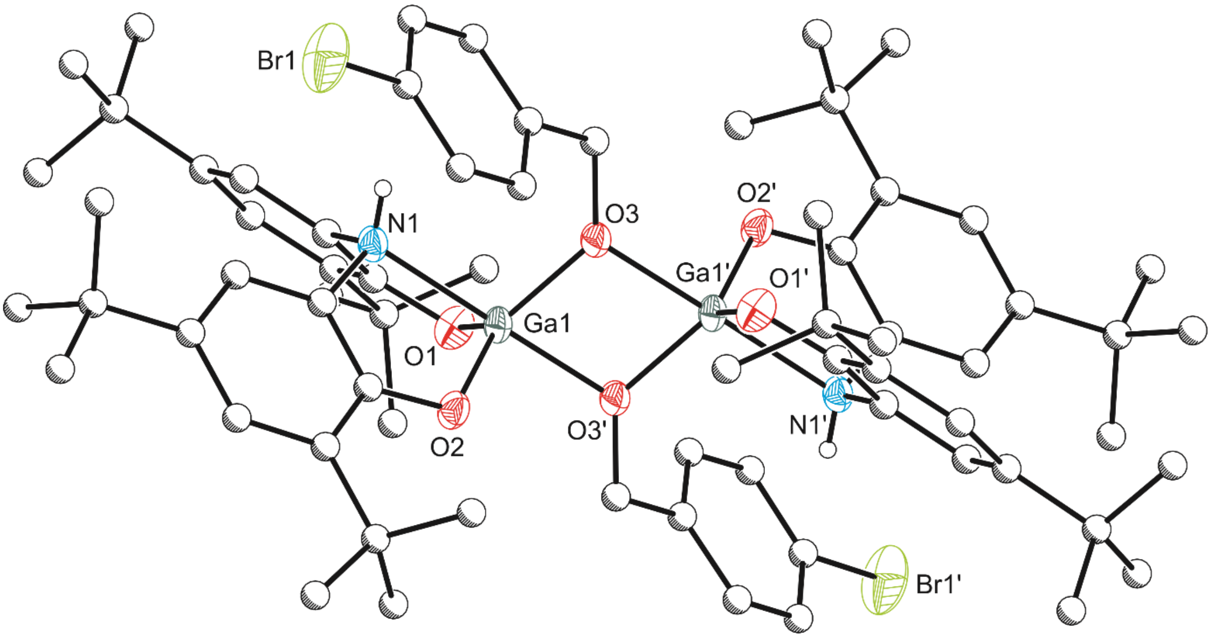

Figure S 85. Molecular structure of **6d**. Anisotropic displacement ellipsoids set at 50% probability. Hydrogen atoms (with the exception of H1) have been omitted for clarity. All carbon atoms are pictured as spheres of arbitrary radius. Selected interatomic distances [Å] and angles [°]: Ga1-O2 1.8324(12), Ga1-O1 1.8409(13), Ga1-O3 1.8775(12), Ga1-O3′ 1.9603(11), Ga1-N1 2.1211(13); O2-Ga1-O1 121.19(6), O2-Ga1-O3 118.26(6), O1-Ga1-O3 120.40(6), O2-Ga1-O3′ 93.99(5), O1-Ga1-O3′ 94.95(5), O3-Ga1-O3′ 76.99(5), O2-Ga1-N1 86.18(5), O1-Ga1-N1 85.76(6), O3-Ga1-N1 102.11(5), O3′-Ga1-N1 179.06(6)

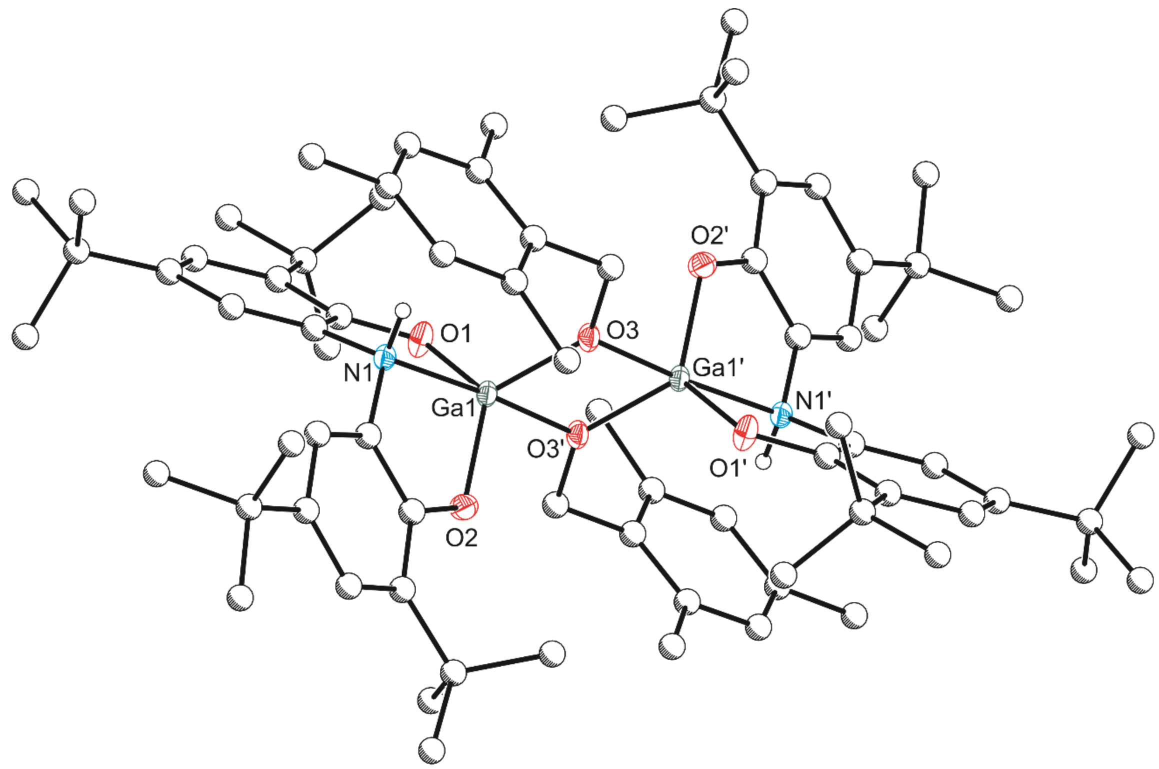

Figure S 86. Molecular structure of **6e**. Anisotropic displacement ellipsoids set at 50% probability. Hydrogen atoms (with the exception of H1) have been omitted for clarity. All carbon atoms are pictured as spheres of arbitrary radius. Selected interatomic distances [Å] and angles [°]: Ga1–O1 1.8486(16), Ga1–O2 1.8609(16), Ga1–O3 1.8836(16), Ga1–O3′ 1.9684(15), Ga1–N1 2.1131(19); O1-Ga1-O2 122.96(8), O1-Ga1-O3 119.01(7), O2-Ga1-O3 117.78(7), O1-Ga1-O3′ 94.00(7), O2-Ga1-O3′ 92.93(7), O3-Ga1-O3′ 77.59(7), O1-Ga1-N1 85.64(7), O2-Ga1-N1 84.15(7), O3-Ga1-N1 105.86(7), O3′-Ga1-N1 176.24(7), Ga1-O3-Ga1′ 102.41(7).

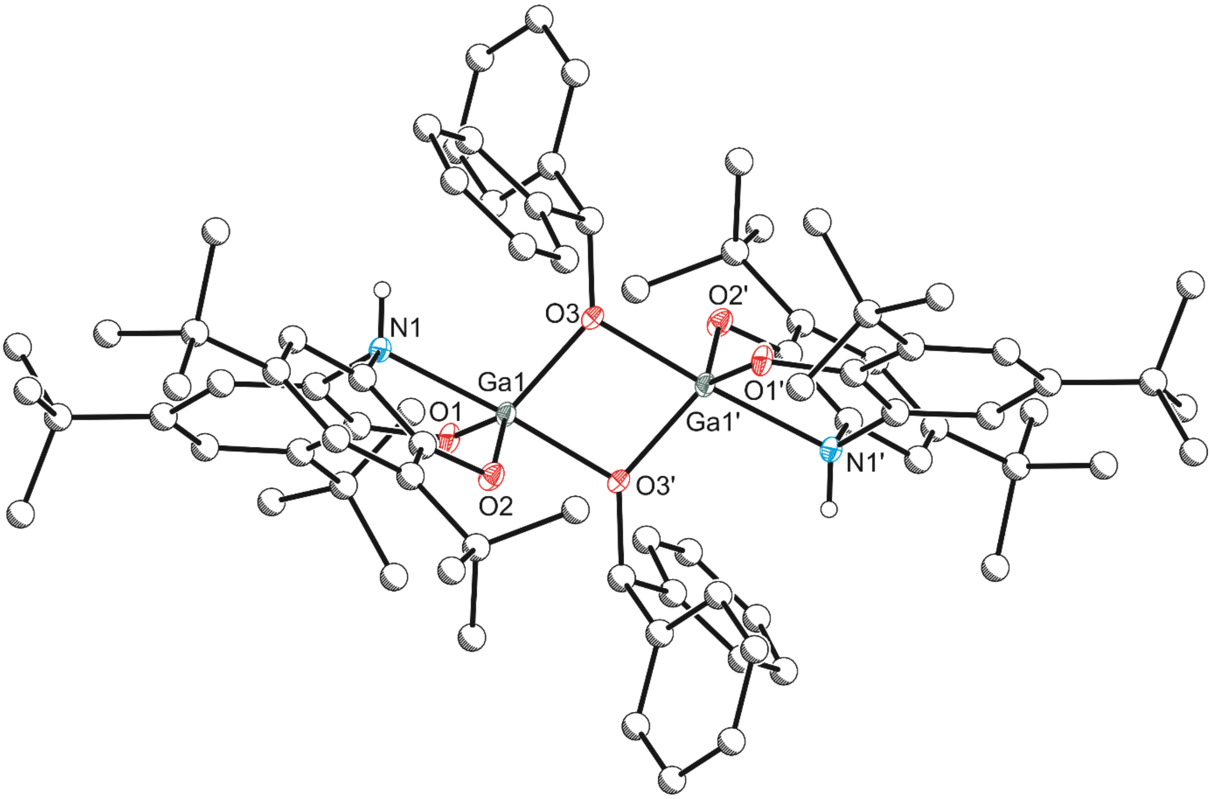

Figure S 87. Molecular structure of **7a**. Anisotropic displacement ellipsoids set at 50% probability. Hydrogen atoms (with the exception of H1) have been omitted for clarity. All carbon atoms are pictured as spheres of arbitrary radius. Selected interatomic distances [Å] and angles [°]: Ga1–O1 1.8610(14), Ga1–O2 1.8431(14), Ga1–O3 1.8899(14), Ga1–O3′ 1.9700(14), Ga1–N1 2.1164(17); O2-Ga1-O1 128.07(7), O2-Ga1-O3 121.18(6), O1-Ga1-O3 110.63(6), O2-Ga1-O3′ 94.73(6), O1-Ga1-O3′ 92.60(6), O3-Ga1-O3′ 77.89(6), O2-Ga1-N1 84.65(6), O1-Ga1-N1 85.06(6), O3-Ga1-N1 105.43(6), O3′-Ga1-N1 176.45(6), Ga1-O3-Ga1′ - 102.12(6).

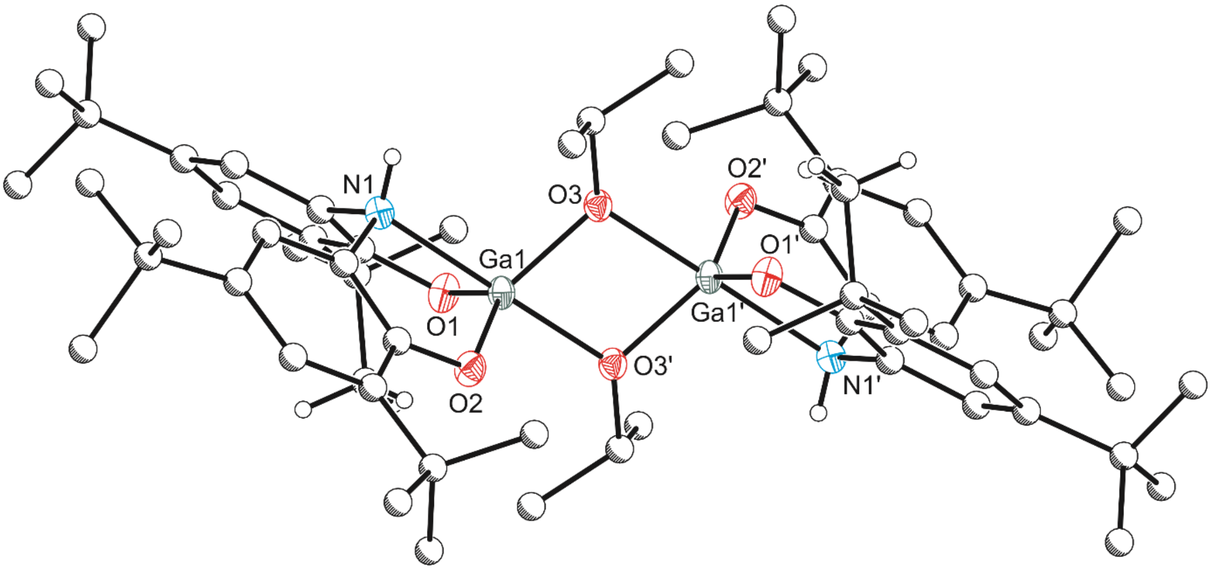

Figure S 88. Molecular structure of **7b**. Anisotropic displacement ellipsoids set at 50% probability. Hydrogen atoms (with the exception of H1) have been omitted for clarity. All carbon atoms are pictured as spheres of arbitrary radius. Selected interatomic distances [Å] and angles [°]: Ga1–O1 1.8431(10), Ga1–O2 1.8423(10), Ga1–O3 1.8834(10), Ga1–O3′ 1.9738(10), Ga1–N1 2.1720(12); O2-Ga1-O1 125.50(5), O2-Ga1-O3 116.60(5), O1-Ga1-O3 117.88(5), O2-Ga1-O3′ 96.55(4), O1-Ga1-O3′ 95.77(4), O3-Ga1-O3′ 78.18(5), O2-Ga1-N1 84.17(5), O1-Ga1-N1 83.96(5),O3-Ga1-N1 101.33(5), O3′-Ga1-N1 179.26(5), Ga1-O3-Ga1′ 101.82(5).

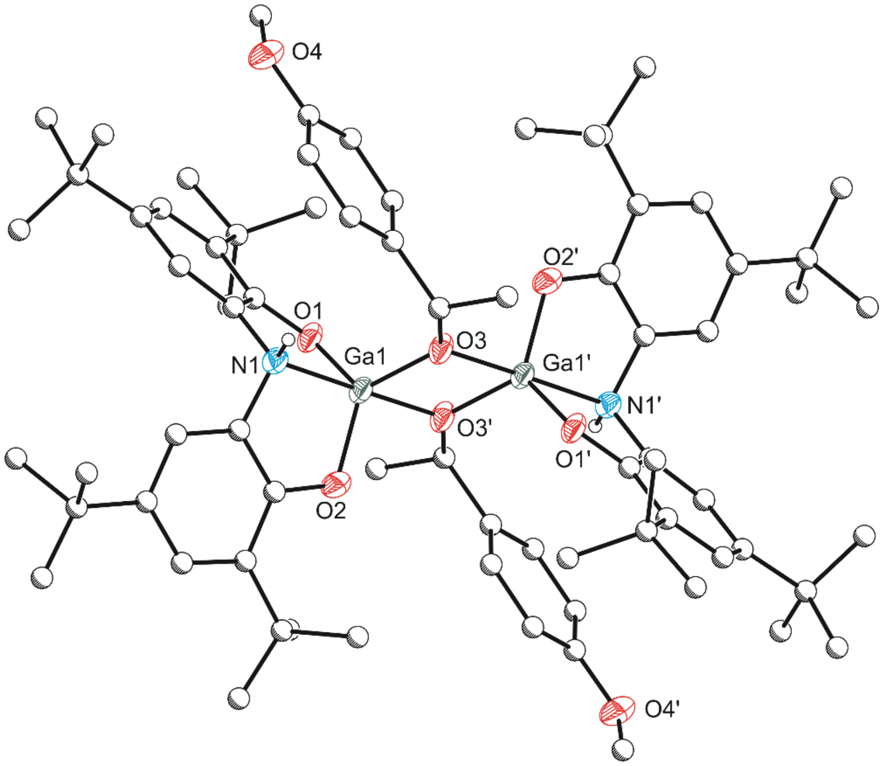

Figure S 89. Molecular structure of **7c**. Anisotropic displacement ellipsoids set at 50% probability. Hydrogen atoms (with the exception of H1) have been omitted for clarity. All carbon atoms are pictured as spheres of arbitrary radius. Selected interatomic distances [Å] and angles [°]: Ga1–O1 1.843(2), Ga1–O2 1.850(2), Ga1–O3 1.8826(19), Ga1–O3′ 1.9684(19), Ga1–N1 2.141(2); O1-Ga1-O2 121.64(9), O1-Ga1-O3 120.19(9), O2-Ga1-O3 118.17(10), O1-Ga1-O3′ 97.01(9), O2-Ga1-O3′ 95.32(9), O3-Ga1-O3′ 77.22(9), O1-Ga1-N1 84.66(9), O2-Ga1-N1 84.95(9), O3-Ga1-N1 100.74(9), O3′-Ga1-N1 177.82(8), Ga1-O3-Ga1′ 102.78(9).

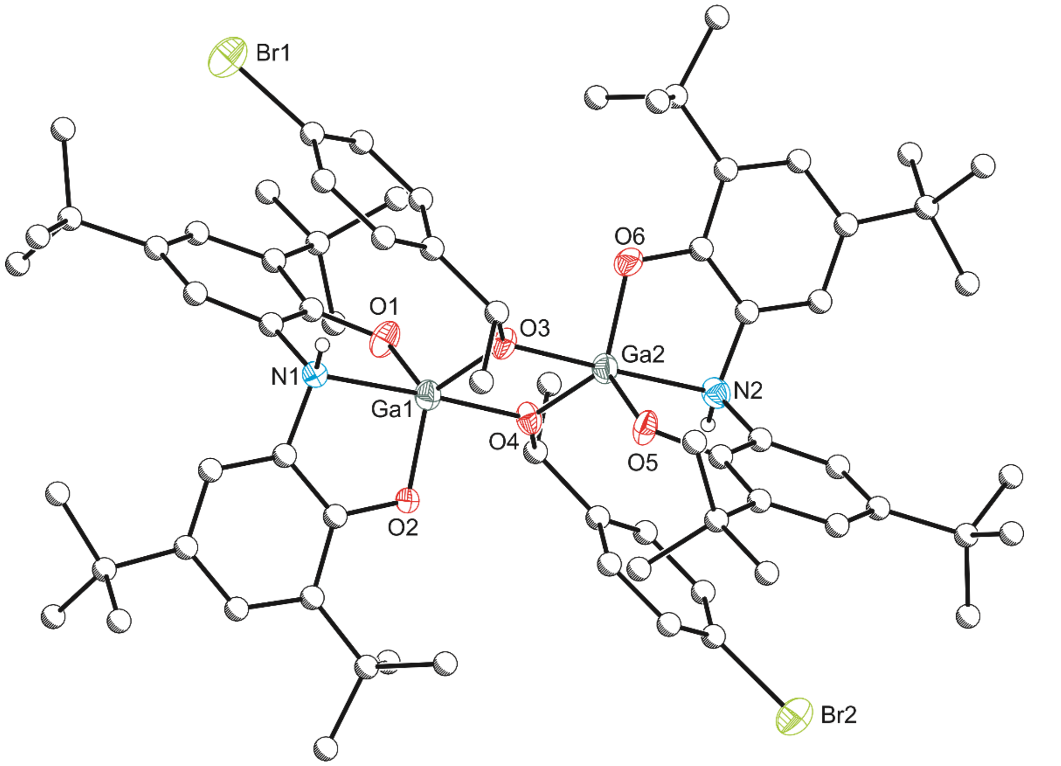

Figure S 90. Molecular structure of **7d**. Anisotropic displacement ellipsoids set at 50% probability. Hydrogen atoms (with the exception of H1) have been omitted for clarity. All carbon atoms are pictured as spheres of arbitrary radius. Selected interatomic distances [Å] and angles [°]: Ga1–O1 1.823(8), Ga1–O2 1.819(7), Ga1–O3 1.876(7), Ga1–O4 1.977(7), Ga1–N1 2.144(8), Ga2-O5 1.838(7), Ga2-O6 1.842(8), Ga2-O4 1.874(7), Ga2-O3 1.976(7), Ga2-N2 2.134(9); O2-Ga1-O1 123.6(4), O2-Ga1-O3 118.1(3), O1-Ga1-O3 118.1(3), O2-Ga1-O4 93.0(3), O1-Ga1-O4 93.9(3), O3-Ga1-O4 77.9(3), O2-Ga1-N1 85.0(3), O1-Ga1-N1 84.9(3), O3-Ga1-N1 105.3(3), O4-Ga1-N1 176.7(3).

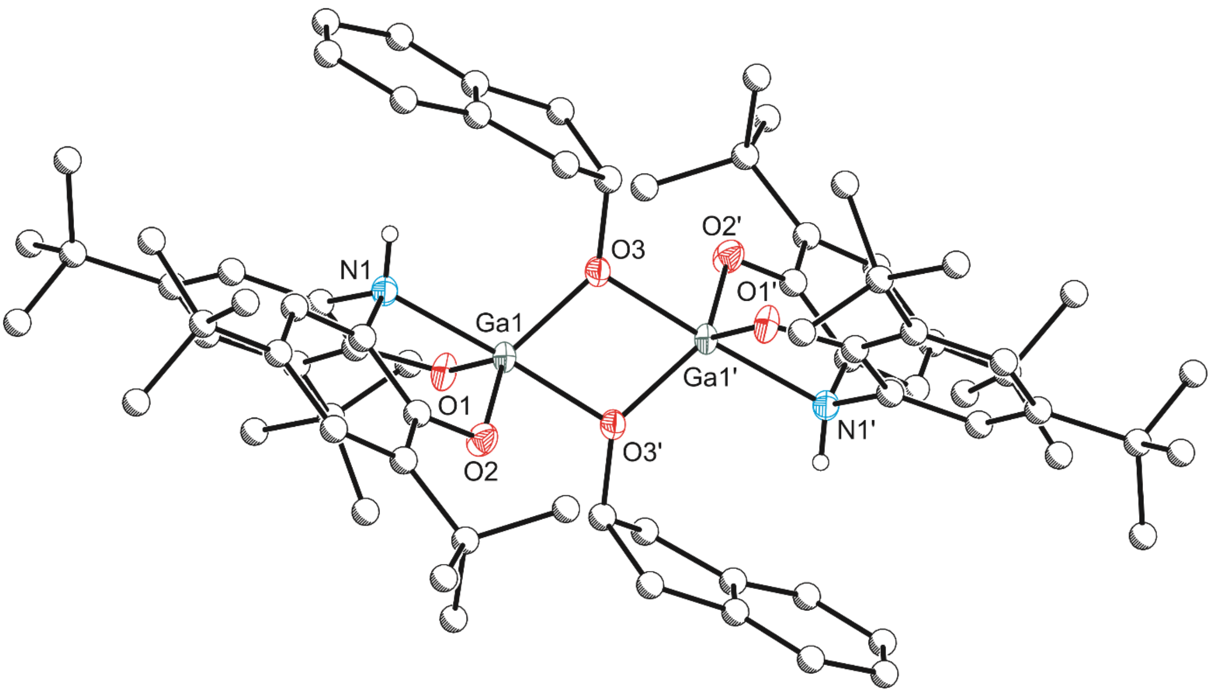

Figure S 91. Molecular structure of **7e**. Anisotropic displacement ellipsoids set at 50% probability. Hydrogen atoms (with the exception of H1) have been omitted for clarity. All carbon atoms are pictured as spheres of arbitrary radius. Selected interatomic distances [Å] and angles [°]: Ga1–O1 1.8464(16), Ga1–O2 1.8413(17), Ga1–O3 1.8814(15), Ga1–O3′ 1.9660(15), Ga1–N1 2.1147(18); O2-Ga1-O1 118.54(8), O2-Ga1-O3 116.55(7), O1-Ga1-O3 124.54(8), O2-Ga1-O3′ 92.32(7), O1-Ga1-O3′ 93.57(7), O3-Ga1-O31 78.24(7), O2-Ga1-N1 85.55(7), O1-Ga1-N1 86.03(7), O3-Ga1-N1 104.18(7), O31-Ga1-N1 177.31(7).

Table S7. Selected X-ray data collection and refinement parameters for **1**, **2**, and **3**.

**Identification code**	**1**	**2**	**3**
Empirical formula	$C_{33}H_{46}ClGaN_2O_2$	$C_{32}H_{49}NO_3GaBr$	$C_{32}H_{50}NO_3Ga$
CCDC deposition number	2103522	2103523	2103524
Formula weight	607.89	645.35	566.45
Temperature/K	150(2)	150(2)	150(2)
Crystal system	monoclinic	monoclinic	monoclinic
Space group	$P2_1/n$	$P2_1/n$	$P2_1/n$
a/Å	10.3777(3)	10.2875(4)	10.5108(4)
b/Å	11.3696(3)	11.3761(4)	11.7221(3)
c/Å	27.6167(8)	27.8336(9)	25.7484(8)
α/°	90	90	90
β/°	100.707(3)	98.959(4)	94.700(3)
γ/°	90	90	90
Volume/Å^3	3201.77(16)	3217.7(2)	3161.76(18)
Z	4	4	4
ρ_{calc}/g cm^{-3}	1.261	1.332	1.19
μ/mm^{-1}	2.171	2.857	1.415
F(000)	1288	1352	1216
Crystal size/mm^3	0.140 × 0.080 × 0.050	0.170 × 0.070 × 0.030	0.180 × 0.080 × 0.040
Radiation	Cu Kα (λ = 1.54184)	Cu Kα (λ = 1.54178)	Cu Kα (λ = 1.54184)
Θ range for data collection/°	4.216 to 76.299	4.206 to 76.191	3.445 to 77.200
Index ranges	$-12 \le h \le 13$, $-14 \le k \le 13$, $-34 \le l \le 24$	$-12 \le h \le 12$, $-14 \le k \le 9$, $-32 \le l \le 34$	$-13 \le h \le 13$, $-10 \le k \le 14$, $-32 \le l \le 32$
Reflections collected	22572	17644	26500
Independent reflections	6641 [R_{int} = 0.0245, R_{sigma} = 0.0215]	6659 [R_{int} = 0.0274, R_{sigma} = 0.0320]	6623 [R_{int} = 0.0563, R_{sigma} = 0.0476]
Data/restraints/parameters	6641/0/356	6659/10/404	6623/0/354
Goodness-of-fit on F^2	1.017	0.988	1.084
Final R indexes [I≥2σ (I)]	R_1 = 0.0259, wR_2 = 0.0642	R_1 = 0.0479, wR_2 = 0.1034	R_1 = 0.0537, wR_2 = 0.1415
Final R indexes [all data]	R_1 = 0.0294, wR_2 = 0.0668	R_1 = 0.0595, wR_2 = 0.1107	R_1 = 0.0639, wR_2 = 0.1483
Largest diff. peak/hole / e Å^{-3}	0.302/−0.328	2.261/−2.592	0.914/−0.615

Table S8. Selected X-ray data collection and refinement parameters for **3**·1.5(BH_3:py), **4a**·4C_6D_6 and **4b**·4tol.

**Identification code**	**3**·1.5(BH_3:py)	**4a**·4C_6D_6	**4b**·4tol
Empirical formula	$C_{39.5}H_{62}B_{1.5}GaN_{2.5}O_3$	$C_{84}H_{116}Ga_2N_2O_6$	$C_{84}H_{124}N_2O_6Ga_2$
CCDC deposition number	2103525	2103526	2103527
Formula weight	705.85	1389.22	1397.28
Temperature/K	150(2)	250(2)	150(2)
Crystal system	monoclinic	triclinic	monoclinic
Space group	*C*2/*c*	*P*−1	$P2_1/c$
a/Å	36.8096(17)	9.9279(4)	14.1156(4)
b/Å	12.9162(5)	14.2823(6)	25.4954(5)
c/Å	17.8272(7)	15.1222(8)	11.7947(3)
α/°	90	73.095(4)	90
β/°	108.325(4)	88.710(4)	113.696(3)
γ/°	90	86.042(3)	90
Volume/$Å^3$	8045.9(6)	2046.67(17)	3886.84(19)
Z	8	1	2
ρ_{calc}/g cm^{-3}	1.165	1.127	1.194
μ/mm^{-1}	1.212	1.181	1.244
F(000)	3032	744	1504
Crystal size/mm^3	0.120 × 0.060 × 0.060	0.120 × 0.120 × 0.060	0.050 × 0.050 × 0.050
Radiation	Cu Kα (λ = 1.54178)	Cu Kα (λ = 1.54184)	Cu Kα (λ = 1.54184)
Θ range for data collection/°	3.648 to 76.097	3.755 to 76.592	7.205 to 89.393
Index ranges	$-45 \le h \le 36$, $-13 \le k \le 15$, $-22 \le l \le 21$	$-12 \le h \le 12$, $-17 \le k \le 17$, $-19 \le l \le 18$	$-18 \le h \le 18$, $-33 \le k \le 33$, $-15 \le l \le 15$
Reflections collected	23137	33243	67190
Independent reflections	8256 [R_{int} = 0.0689, R_{sigma} = 0.0783]	8495 [R_{int} = 0.0616, R_{sigma} = 0.0481]	8876 [R_{int} = 0.0558, R_{sigma} = 0.0374]
Data/restraints/parameters	8256/12/507	8495/138/496	8876/0/440
Goodness-of-fit on F^2	1.034	1.051	1.061
Final R indexes [I≥2σ (I)]	R_1 = 0.0476, wR_2 = 0.1147	R_1 = 0.0621, wR_2 = 0.1563	R_1 = 0.0400, wR_2 = 0.0975
Final R indexes [all data]	R_1 = 0.0922, wR_2 = 0.1323	R_1 = 0.0734, wR_2 = 0.1720	R_1 = 0.0527, wR_2 = 0.1032
Largest diff. peak/hole / e $Å^{-3}$	0.586/−0.509	1.129/−0.531	0.612/−0.724

Table S9. Selected X-ray data collection and refinement parameters for **5**·1.5tol, **6a**·3tol and **6b**·3tol.

**Identification code**	**5**·1.5tol	**6a**·3tol	**6b**·3tol
Empirical formula	$C_{66.5}H_{96}Ga_2N_2O_4$	$C_{91}H_{120}Ga_2N_2O_6$	$C_{85}H_{124}Ga_2N_2O_6$
CCDC deposition number	2103528	2103529	2103530
Formula weight	1126.89	1477.32	1409.29
Temperature/K	150(2)	150(2)	150(2)
Crystal system	triclinic	triclinic	triclinic
Space group	*P*−1	*P*−1	*P*−1
a/Å	13.2508(6)	10.1748(4)	10.9400(4)
b/Å	16.1097(7)	13.5915(7)	10.9575(4)
c/Å	16.2894(4)	16.6911(11)	19.5421(8)
α/°	100.939(3)	73.425(5)	83.885(4)
β/°	95.296(3)	87.080(4)	85.067(3)
γ/°	110.593(4)	71.718(4)	61.298(4)
Volume/$Å^3$	3147.7(2)	2098.8(2)	2041.45(15)
Z	2	1	1
ρ_{calc}/g cm^{-3}	1.189	1.169	1.146
μ/mm^{-1}	1.397	1.182	1.189
F(000)	1206	790	758
Crystal size/mm^3	0.140 × 0.100 × 0.080	0.130 × 0.070 × 0.030	0.230 × 0.130 × 0.100
Radiation	Cu Kα (λ = 1.54184)	Cu Kα (λ = 1.54178)	Cu Kα (λ = 1.54178)
Θ range for data collection/°	2.805 to 66.489	3.572 to 66.486	4.555 to 76.187
Index ranges	$-15 \leq h \leq 15$, $-19 \leq k \leq 19$, $-19 \leq l \leq 16$	$-12 \leq h \leq 9$, $-15 \leq k \leq 16$, $-19 \leq l \leq 19$	$-13 \leq h \leq 8$, $-13 \leq k \leq 11$, $-24 \leq l \leq 24$
Reflections collected	24362	18902	19231
Independent reflections	11087 [R_{int} = 0.0509, R_{sigma} = 0.0703]	7381 [R_{int} = 0.0650, R_{sigma} = 0.0825]	8342 [R_{int} = 0.0304, R_{sigma} = 0.0392]
Data/restraints/parameters	11087/134/743	7381/51/570	8342/55/535
Goodness-of-fit on F^2	1.057	0.853	1.084
Final R indexes [I≥2σ (I)]	R_1 = 0.0487, wR_2 = 0.1178	R_1 = 0.0518, wR_2 = 0.1149	R_1 = 0.0559, wR_2 = 0.1480
Final R indexes [all data]	R_1 = 0.0676, wR_2 = 0.1283	R_1 = 0.0730, wR_2 = 0.1246	R_1 = 0.0618, wR_2 = 0.1523
Largest diff. peak/hole / e $Å^{-3}$	1.072/−0.743	0.516/−0.692	1.972/−0.875

Table S10. Selected X-ray data collection and refinement parameters for **6c**·$2C_6D_6$, **6d**·$2C_6D_6$ and **6e**·$2CDCl_3$.

**Identification code**	**6c**·$2C_6D_6$	**6d**·$2C_6D_6$	**6e**·$2CDCl_3$
Empirical formula	$C_{84}H_{112}Ga_2N_2O_8$	$C_{82}H_{106}Br_2Ga_2N_2O_6$	$C_{78}H_{110}N_2O_6Ga_2Cl_6$
CCDC deposition number	2103531	2103532	2103533
Formula weight	1417.19	1514.94	1523.81
Temperature/K	150(2)	150(2)	150(2)
Crystal system	triclinic	triclinic	triclinic
Space group	*P*−1	*P*−1	*P*−1
a/Å	11.7351(3)	10.2092(5)	10.0474(6)
b/Å	13.6580(3)	14.1435(5)	13.9132(8)
c/Å	13.7914(4)	14.8323(4)	15.0745(7)
α/°	118.346(3)	71.491(3)	73.849(5)
β/°	91.183(2)	86.381(3)	89.590(4)
γ/°	98.843(2)	81.607(3)	82.391(5)
Volume/$Å^3$	1911.12(10)	2008.84(14)	2005.3(2)
Z	1	1	1
ρ_{calc}/g cm^{-3}	1.231	1.252	1.262
μ/mm^{-1}	1.297	2.369	3.046
F(000)	756	792	804
Crystal size/mm^3	0.110 × 0.070 × 0.040	0.180 × 0.160 × 0.140	0.120 × 0.080 × 0.040
Radiation	Cu Kα (λ = 1.54184)	Cu Kα (λ = 1.54184)	Cu Kα (λ = 1.54184)
Θ range for data collection/°	3.663 to 76.719	3.143 to 76.981	3.843 to 66.495
Index ranges	$-14 \le h \le 14$, $-17 \le k \le 17$, $-17 \le l \le 17$	$-12 \le h \le 12$, $-16 \le k \le 17$, $-18 \le l \le 14$	$-11 \le h \le 11$, $-11 \le k \le 16$, $-17 \le l \le 17$
Reflections collected	46696	16435	17880
Independent reflections	7940 [R_{int} = 0.0318, R_{sigma} = 0.0196]	8349 [R_{int} = 0.0244, R_{sigma} = 0.0387]	7048 [R_{int} = 0.0423, R_{sigma} = 0.0542]
Data/restraints/parameters	7940/0/506	8349/0/429	7048/0/443
Goodness-of-fit on F^2	1.034	1.024	1.031
Final R indexes [I≥2σ (I)]	R_1 = 0.0299, wR_2 = 0.0777	R_1 = 0.0323, wR_2 = 0.0778	R_1 = 0.0421, wR_2 = 0.1041
Final R indexes [all data]	R_1 = 0.0326, wR_2 = 0.0798	R_1 = 0.0404, wR_2 = 0.0828	R_1 = 0.0537, wR_2 = 0.1120
Largest diff. peak/hole / e $Å^{-3}$	0.376/−0.356	0.410/−0.552	0.967/−0.584

Table S11. Selected X-ray data collection and refinement parameters for **7a**·4$CDCl_3$, **7b**·3C_6D_6 and **7c**·2C_6D_6.

**Identification code**	**7a**·4$CDCl_3$	**7b**·3C_6D_6	**7c**·2C_6D_6
Empirical formula	$C_{86}H_{108}Cl_{12}Ga_2N_2O_6$	$C_{80}H_{114}Ga_2N_2O_6$	$C_{86}H_{116}Ga_2N_2O_8$
CCDC deposition number	2103534	2103535	2103536
Formula weight	1830.58	1339.17	1445.24
Temperature/K	150(2)	150(2)	150(2)
Crystal system	monoclinic	monoclinic	triclinic
Space group	$P2_1/n$	$P2_1/c$	$P-1$
a/Å	13.2386(2)	13.4385(2)	11.9141(4)
b/Å	23.2866(4)	25.7074(3)	13.7371(9)
c/Å	14.7290(3)	11.6672(2)	13.9224(9)
α/°	90	90	60.696(6)
β/°	98.551(2)	110.313(2)	80.943(4)
γ/°	90	90	80.598(4)
Volume/Å^3	4490.21(14)	3779.99(11)	1952.4(2)
Z	2	2	1
ρ_{calc}/g cm^{-3}	1.354	1.177	1.229
μ/mm^{-1}	4.420	1.260	1.279
F(000)	1904	1436	772
Crystal size/mm^3	0.200 × 0.200 × 0.200	0.200 × 0.160 × 0.060	0.200 × 0.100 × 0.100
Radiation	Cu Kα (λ = 1.54184)	Cu Kα (λ = 1.54184)	Cu Kα (λ = 1.54184)
Θ range for data collection/°	3.579 to 76.138	3.438 to 76.720	3.655 to 76.491
Index ranges	$-15 \le h \le 16$, $-29 \le k \le 23$, $-17 \le l \le 18$	$-16 \le h \le 16$, $-32 \le k \le 32$, $-14 \le l \le 13$	$-14 \le h \le 10$, $-16 \le k \le 17$, $-17 \le l \le 17$
Reflections collected	26576	39859	17832
Independent reflections	9303 [R_{int} = 0.0423, R_{sigma} = 0.0411]	7923 [R_{int} = 0.0464, R_{sigma} = 0.0317]	8068 [R_{int} = 0.0270, R_{sigma} = 0.0331]
Data/restraints/parameters	9303/0/217	7923/114/481	8068/0/460
Goodness-of-fit on F^2	1.035	1.017	0.945
Final R indexes [I≥2σ (I)]	R_1 = 0.0401, wR_2 = 0.0942	R_1 = 0.0316, wR_2 = 0.0791	R_1 = 0.0585, wR_2 = 0.1718
Final R indexes [all data]	R_1 = 0.0512, wR_2 = 0.1034	R_1 = 0.0395, wR_2 = 0.0846	R_1 = 0.0657, wR_2 = 0.1818
Largest diff. peak/hole / e Å^{-3}	0.883/−0.722	0.343/−0.336	2.721/−0.804

Table S12. Selected X-ray data collection and refinement parameters for **7d**·$2C_6D_6$ and **7e**·$2CDCl_3$.

**Identification code**	**7d**·$2C_6D_6$	**7e**·$2CDCl_3$
Empirical formula	$C_{84}H_{110}Br_2Ga_2N_2O_6$	$C_{76}H_{102}Cl_6Ga_2N_2O_6$
CCDC deposition number	2103537	2103538
Formula weight	1542.99	1491.73
Temperature/K	150(2)	150(2)
Crystal system	orthorhombic	monoclinic
Space group	$Pca2_1$	$P2_1/c$
a/Å	12.3203(6)	17.7872(2)
b/Å	17.7668(9)	18.4325(2)
c/Å	37.0079(14)	12.10850(10)
α/°	90	90
β/°	90	104.5250(10)
γ/°	90	90
Volume/Å^3	8100.7(6)	3843.04(7)
Z	4	2
ρ_{calc}/g cm^{-3}	1.265	1.289
μ/mm^{-1}	1.702	3.169
F(000)	3232	1568
Crystal size/mm^3	0.300 × 0.080 × 0.030	0.200 × 0.160 × 0.060
Radiation	Mo Kα (λ = 0.71073)	Cu Kα (λ = 1.54184)
Θ range for data collection/°	3.303 to 24.998	3.513 to 76.186
Index ranges	$-14 \le h \le 13$, $-21 \le k \le 17$, $-41 \le l \le 44$	$-22 \le h \le 22$, $-23 \le k \le 21$, $-15 \le l \le 12$
Reflections collected	33972	22979
Independent reflections	13206 [R_{int} = 0.1107, R_{sigma} = 0.1629]	7948 [R_{int} = 0.0348, R_{sigma} = 0.0391]
Data/restraints/parameters	13206/1/891	7948/0/419
Goodness-of-fit on F^2	0.911	1.037
Final R indexes [I≥2σ (I)]	R_1 = 0.0625, wR_2 = 0.0932	R_1 = 0.0443, wR_2 = 0.1160
Final R indexes [all data]	R_1 = 0.1366, wR_2 = 0.1145	R_1 = 0.0562, wR_2 = 0.1266
Largest diff. peak/hole / e Å^{-3}	0.566/−0.503	1.185/−0.598

6. References

[1] P. Chaudhuri, M. Hess, T. Weyhermüller, K. Wieghardt, *Angew.Chem. Int. Ed.* **1999**, *38*, 1095–1098.

[2] G. R. Fulmer, A. J. M. Miller, N. H. Sherden, H. E. Gottlieb, A. Nudelman, B. M. Stoltz, J. E. Bercaw, K. I. Goldberg, *Organometallics* **2010**, *29*, 2176–2179.

[3] R. Evans, Z. Deng, A. K. Rogerson, A. S. McLachlan, J. J. Richards, M. Nilsson, G. A. Morris, *Angew.Chem. Int. Ed.* **2013**, *52*, 3199–3202.

[4] R. Evans, G. Dal Poggetto, M. Nilsson, G. A. Morris, *Anal. Chem.* **2018**, *90*, 3987–3994.

[5] A. D. Bage, T. A. Hunt, S. P. Thomas, *Org. Lett.* **2020**, *22,* 4107–4112.

[6] J. Burés, *Angew.Chem. Int. Ed.* **2016**, *55*, 16084–16087.

[7] R. Hooke and T. A. Jeeves. *J. ACM*. **1961**, *8*, 212–229.

[8] *CrysAlisPro,* Agilent Technologies, Version 1.171.35.8.

[9] (a) G. M. Sheldrick in SHELXL97, *Programs for Crystal Structure Analysis (Release 97-2),* Institut für Anorganische Chemie der Universität, Tammanstrasse 4, D-3400 Göttingen, Germany, **1998**; (b) G. M. Sheldrick, *Acta Crystallogr. Sect. A.* **1990**, *46*, 467−473; (c) G. M. Sheldrick, *Acta Crystallogr. Sect. A.* **2008**, *64*, 112−122.
